# Nanoscale Assembly of Functional Peptides with Divergent Programming Elements

**DOI:** 10.1021/acsnano.0c09386

**Published:** 2021-02-12

**Authors:** Ana M. Garcia, Michele Melchionna, Ottavia Bellotto, Slavko Kralj, Sabrina Semeraro, Evelina Parisi, Daniel Iglesias, Paola D’Andrea, Rita De Zorzi, Attilio V. Vargiu, Silvia Marchesan

**Affiliations:** † Chemical and Pharmaceutical Sciences Department, University of Trieste, Via Giorgieri 1, 34127 Trieste, Italy; ‡ INSTM, University of Trieste, Via Giorgieri 1, 34127 Trieste, Italy; § Materials Synthesis Department, Jožef Stefan Institute, Jamova 39, 1000 Ljubljana, Slovenia; ∥ Life Sciences Department, University of Trieste, Via Giorgieri 1, 34127 Trieste, Italy; ⊥ Physics Department, University of Cagliari, S.P. 8, km. 0.700, 09042 Monserrato, Italy

**Keywords:** peptide, self-assembly, chirality, amyloid, hydrogels, proline, d-amino acids

## Abstract

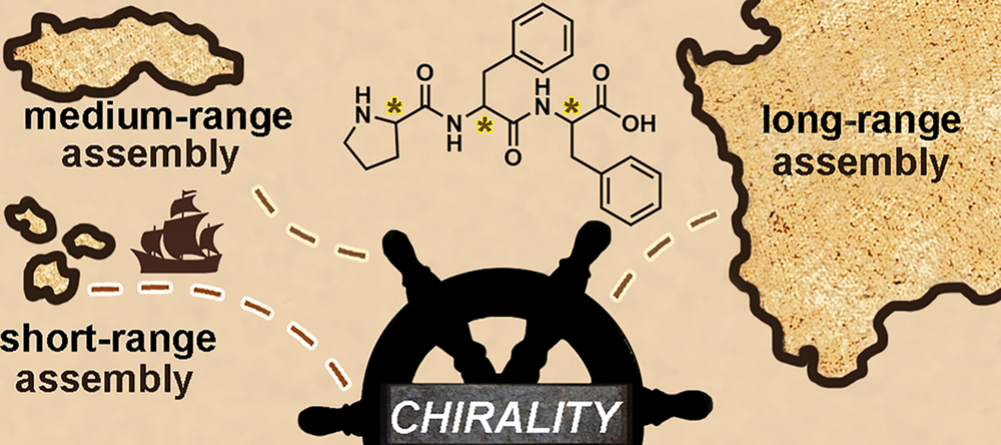

Self-assembling peptides are being applied both in the biomedical area and as building blocks in nanotechnology. Their applications are closely linked to their modes of self-assembly, which determine the functional nanostructures that they form. This work brings together two structural elements that direct nanoscale self-association in divergent directions: proline as a β-breaker and the β-structure-associated diphenylalanine motif, into a single tripeptide sequence. Amino acid chirality was found to resolve the tension inherent to these conflicting self-assembly instructions. Stereoconfiguration determined the ability of each of the eight possible Pro-Phe-Phe stereoisomers to self-associate into diverse nanostructures, including nanoparticles, nanotapes, or fibrils, which yielded hydrogels with gel-to-sol transition at a physiologically relevant temperature. Three single-crystal structures and all-atom molecular dynamics simulations elucidated the ability of each peptide to establish key interactions to form long-range assemblies (*i,e.*, stacks leading to gelling fibrils), medium-range assemblies (*i.e.*, stacks yielding nanotapes), or short-range assemblies (*i.e.*, dimers or trimers that further associated into nanoparticles). Importantly, diphenylalanine is known to serve as a binding site for pathological amyloids, potentially allowing these heterochiral systems to influence the fibrillization of other biologically relevant peptides. To probe this hypothesis, all eight Pro-Phe-Phe stereoisomers were tested *in vitro* on the Alzheimer’s disease-associated Aβ(1–42) peptide. Indeed, one nonfibril-forming stereoisomer effectively inhibited Aβ fibrillization through multivalent binding between diphenylalanine motifs. This work thus defined heterochirality as a useful feature to strategically develop future therapeutics to interfere with pathological processes, with the additional value of resistance to protease-mediated degradation and biocompatibility.

Simple peptides that self-assemble into functional nanostructures have been attracting great interest for their use in medicine and nanotechnology.^1−4^ They are extremely versatile building blocks capable of generating diverse nanomorphologies that are often based on β-sheets.^5,6^ However, the uncontrolled growth of β-sheets and, more generally, peptide assemblies, remains an unsolved general challenge,^7^ and approaches are continuously developed to address it. The vast majority of these exploit solvent-induced effects,^8−10^ or chemical modification of the building blocks to attain morphological diversity^9,11^ or to control supramolecular behavior by means of chemical switches.^12,13^

Proline (Pro) is a well-known β-breaker moiety that interferes with β-sheet formation and may be used to limit β-sheet growth,^14^ which instead can be favored by the incorporation of the diphenylalanine (Phe-Phe) motif.^15,16^ The incorporation of both Pro and Phe in short self-assembling sequences^17,18^ thus encodes two conflicting sets of self-assembly instructions, leading to outcomes that are difficult to predict *a priori*. An elegant computational study by Tuttle and colleagues identified Pro-Phe-Phe as the most aggregating-prone sequence among all possible 8000 combinations of 20 l-natural amino acids in tripeptides.^19^ Their simulations were based on hydrophobicity-related parameters, and subsequent experimental data evidenced a β-sheet-like spectroscopic signature for l-Pro-Phe-Phe aggregates suspended in water, despite the presence of Pro.^19^ Bera *et al.* noted the surprising rearrangement, over the course of several days, of l-Pro-Phe-Phe aggregates into nanofibers with a distinctive α-helix spectroscopic signature.^18^ It is worth noting that the l-Ala-Phe-Phe analog displayed instead spectra reminiscent of β-sheets, confirming the β-breaker role played by Pro.^18^

The incorporation of d-amino acids into l-peptides can lead to unexpected effects on conformation, self-assembly, and even therapeutic activity, as recently reviewed.^20^ Research is revealing the use of heterochirality to fine-tune peptide self-assembly into a variety of nanostructures, including twisted nanofibrils,^21,22^ nanotapes,^23,24^ nanotubes,^25,26^ 2D-sheets,^27^ or gels.^28,29^d-amino acids can inhibit pathological amyloid aggregation, as with d-Phe^30^ on l-Phe fibrillization linked to phenylketonuria,^31^ or d-Trp-Aib (Aib = aminoisobutyrric acid) on Alzheimer’s disease-related Aβ peptide.^32^ Peptide inhibitors that combine a recognition motif with β-breaking Pro demonstrated promising activity *in vitro* and *in vivo*.^14^ Yet, their use as therapeutics has been hampered by their susceptibility to enzymatic hydrolysis and, consequently, short half-lives.^33^ In addition, peptides longer than two-to-three amino acids are costly to produce on a large scale, and it is thus preferable to shorten the sequence as much as possible for applications.

This study explores the introduction of d- and l-amino acids in the sequence Pro-Phe-Phe (Chart 1) to resolve the conflict between the contradictory assembly directing effects of Pro as β-breaker and Phe-Phe as β-sheet-associated motif, enabling the emergence of functional nanostructures. To this end, the self-organizing behavior of the eight distinct stereoisomers of Pro-Phe-Phe was probed, and they were tested *in vitro* as inhibitors of aggregation of the Aβ(1–42) peptide, which is associated with Alzheimer’s disease. The most promising compound was also probed for resistance against protease-mediated hydrolysis and cytocompatibility in cell culture.

**Chart 1:**
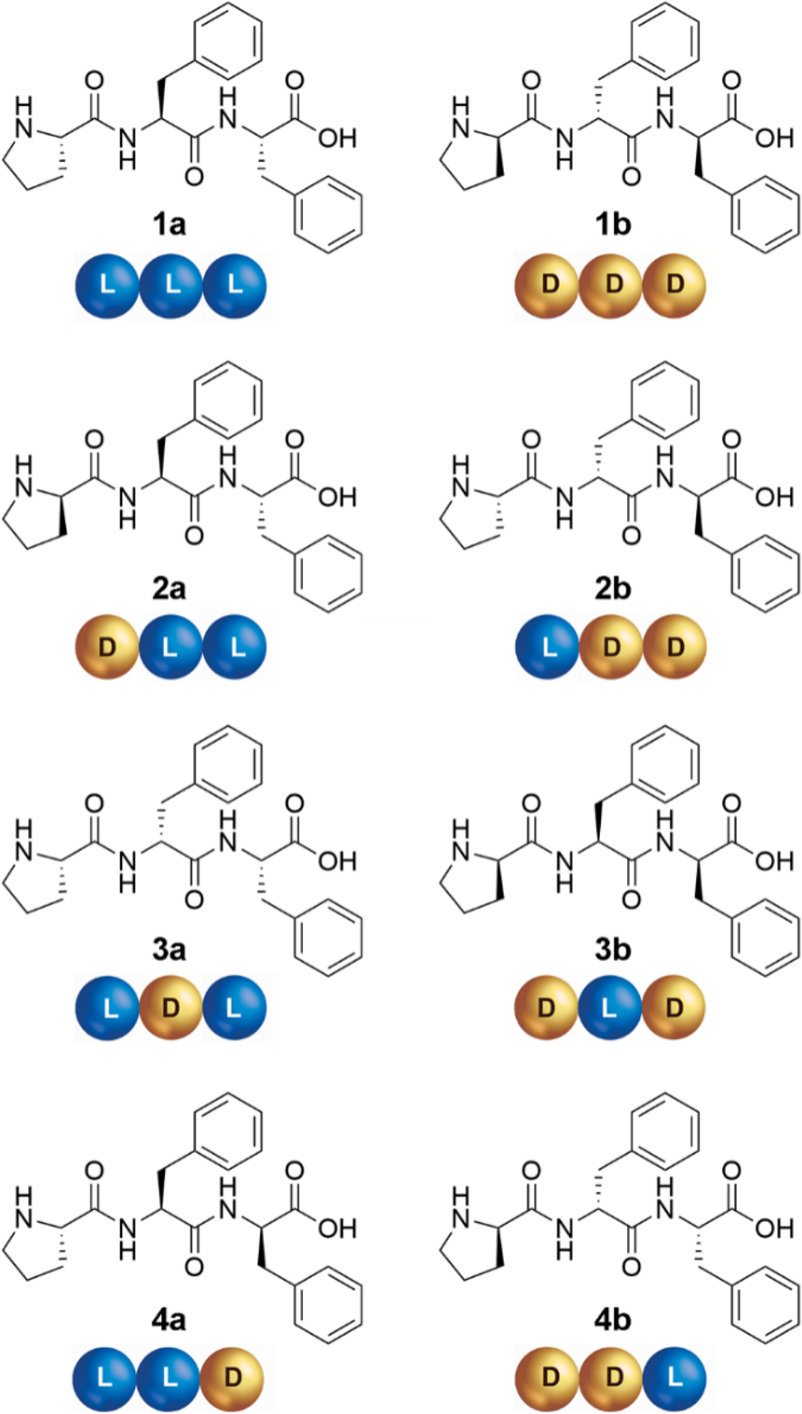
Pro-Phe-Phe Stereoisomers by Enantiomer Pairs (**a**/**b**) and Their Cartoon Keycode (Each Amino Acid is Represented as Either a Blue (l) or Gold (d) Sphere)

## Results and Discussion

### Self-Assembly of Pro-Phe-Phe Stereoisomers into Hydrogels

**Figure 1:**
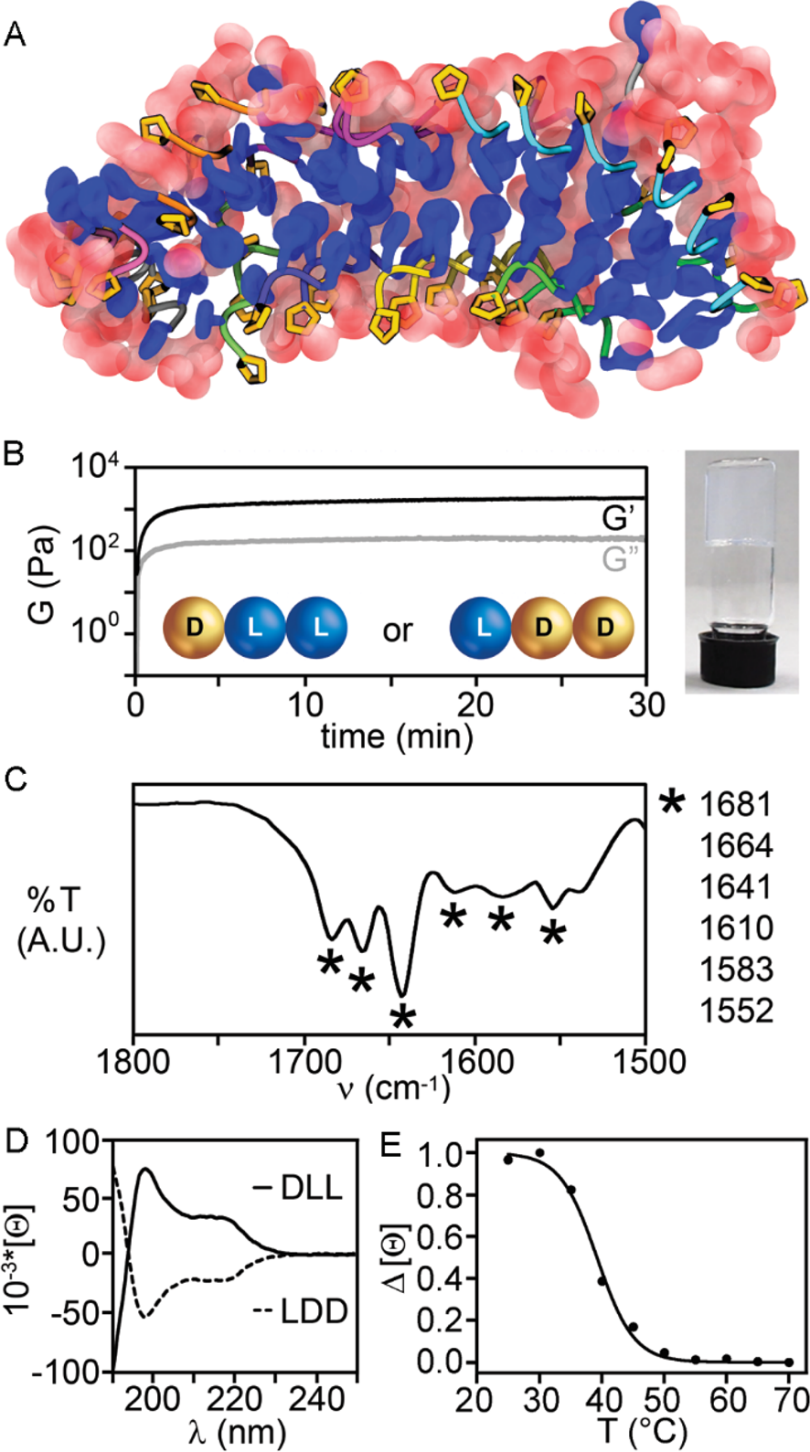
(A) All-atom molecular dynamics (MD) revealing the formation of stacks of kinked peptides for **2a** with Phe residues (blue) facing each other to define water-excluding hydrophobic surfaces, and Pro residues (gold) facing the outer surface to interact with water (red spheres). (B) Rheology time sweep and photograph of **2a** hydrogel. (C) ATR-IR of the gel of **2a**. (D) CD spectra of **2a** and **2b**. (E) CD heating ramp for **2a**.

The eight stereoisomers of Pro-Phe-Phe were prepared by solid-phase peptide synthesis.^34^ Self-assembly studies were performed in phosphate buffer, following a pH switch from alkaline to neutral. Since enantiomeric pairs display the same supramolecular behavior in achiral environments, we report below only the data for one compound (**a**) for each pair (**a**/**b**). Interestingly, among the four stereoisomers **1**–**4**, only **2a** was observed to stack (Figure 1A) so as to form self-supportive hydrogels (Figure 1B). All-atom molecular dynamics (MD, Figure 1A) simulations in explicit water solvent confirmed a kinked backbone for the peptides when assembled, and their ability to give rise to parallel stacks reminiscent of β-sheets (see Movie S1 and Section S6 of the Supporting Information). The amphipathic character of the assembly, with the net segregation between hydrophilic and hydrophobic components, allowed the formation of dry surfaces involving Phe aromatic residues (Figure 1A). Oscillatory rheometry time sweeps at the minimum gelling concentration (mgc, 24 mM) revealed immediate gelation leading to a *G*′ of 4 kPa at plateau (Figure 1B). The hydrogel network was rather fragile, requiring only a stress of 6 Pa to break it (see Supporting Information, Section S3). The higher mgc and the lower elastic modulus *G*′, relative to the analogous Val-Phe-Phe DLL/LDD enantiomers,^24^ suggested self-assembly was partially hindered by the β-breaker proline. This hypothesis was also supported by Thioflavin T (ThT) fluorescence, which was reduced in the case of **2a** relative to its DLL analogue Val-Phe-Phe (see Supporting Information, Section S11). ThT staining is a standard method to quantify amyloids, as this dye is known to bind laterally to fibrils composed of at least four consecutive β-strands of l-peptides, leading to fluorescence.^35^

Attenuated total reflection Fourier transformed infrared (ATR-FT-IR) spectroscopy on the assembled gel (Figure 1C) revealed amide I band frequencies reminiscent of β-structure signatures, such as sheets and turns, at 1610 and 1681 cm^–1^, as well as disordered structures at 1641 cm^–1^. The insertion of d-Pro in l-peptides is a well-known strategy to promote turns.^36^ However, an analogous effect in a sequence as short as a tripeptide is far from sure, particularly as the term “turn” implies longer sequences.^37^ Circular dichroism (CD) spectra (Figure 1D) of the enantiomers were mirror images, as expected. Two peaks were observed at 198 and 219 nm, with an overall signature analogous to those of heterochiral tripeptides Phe-Xaa-Phe (Xaa = Ala, Val, Leu, *etc.*).^38^ In those cases, *in silico* and experimental data indicated that the signal corresponded to populations of different conformations in solution. The most stable ones, which were amplified during assembly, had a backbone conformation similar to that observed for β-structures, with torsion angles of their central residue falling in the β-sheet region of the Ramachandran plot.^38^ We inferred an analogous case here, where the peptides adopted a variety of conformations in solution, yet it was those leading to self-assembly that were predominant in the FT-IR spectra of the gel. Reversible gel-to-sol transition occurred at the physiologically relevant onset temperature of 39 °C, which is convenient for potential use in drug delivery, and these data were confirmed by CD heating ramps (Figure 1E). Hyperthermia-induced drug release is a hot area of research for the topical delivery of chemotherapeutics to treat a variety of tumors, while diminishing the side effects normally associated with systemic use.^39,40^ In particular, if a drug was embedded in a supramolecular hydrogel, its release could be controlled and sustained over time upon thermo-induced hydrogel disassembly by local heating.^41^ The gelator concentration could also be used as a convenient variable to control release kinetics; for instance, when immersed in a hot bath at 40 °C, hydrogels formed by **2a** at 24 and 30 mM disassembled into a clear solution in 25 and 90 min, respectively.

### Nanostructure Morphology

**Figure 2:**
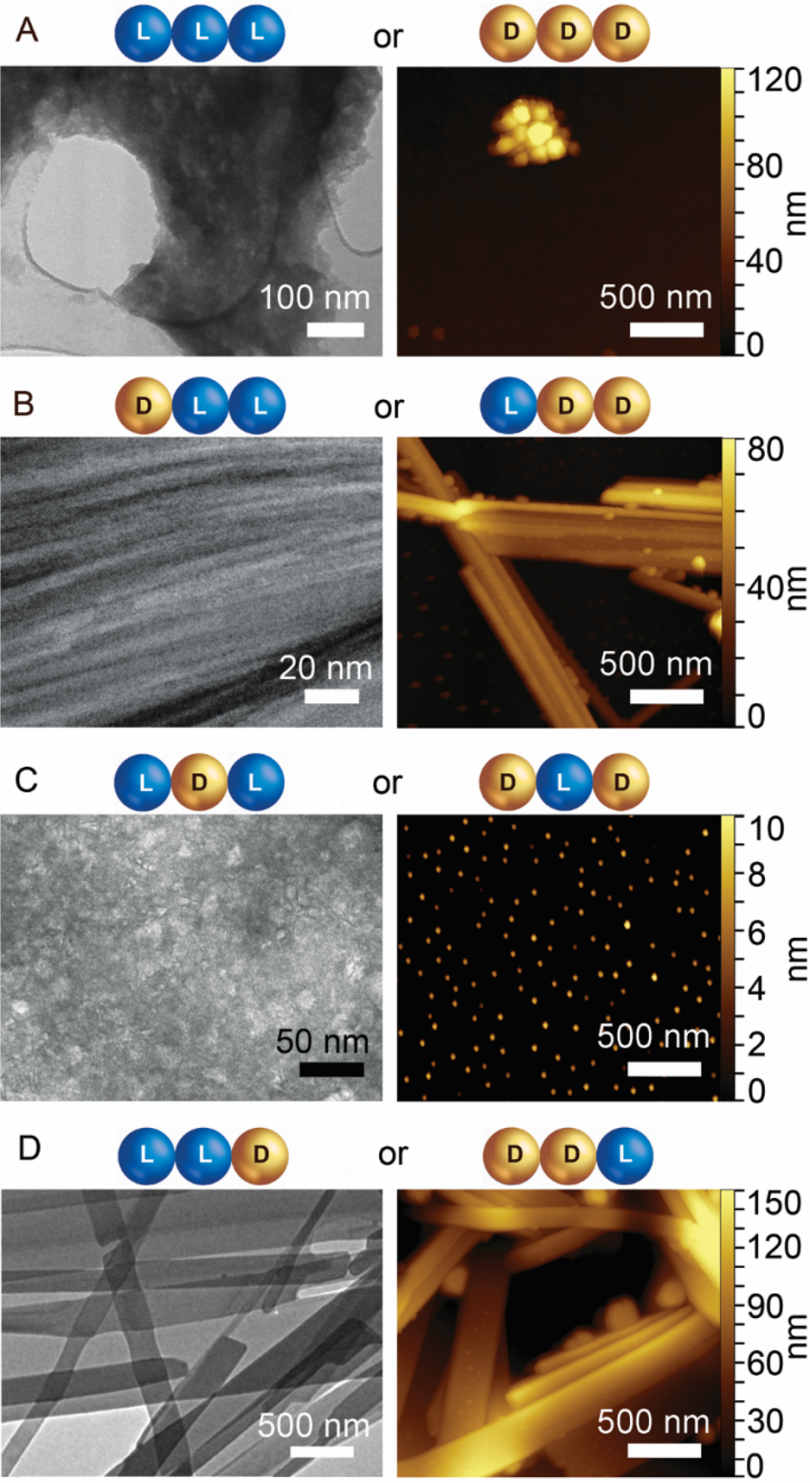
TEM (left) and AFM (right) images. (A) **1a** aggregates; (B) **2a** bundles of nanofibrils; (C) **3a** nanoparticles; (D) **4a** nanotapes. Full-size images and further details can be found in the Supporting Information Sections S4 and S5.

Transmission electron microscopy (TEM) and atomic force microscopy (AFM) images revealed diverse nanostructures formed by the four enantiomeric pairs (Figure 2 and Supporting Information, Sections S4 and S5), thus confirming a directing effect of stereoconfiguration on nanoscale assembly. The homochiral sequence **1a** (Figure 2A) formed mainly amorphous aggregates, and occasionally microcrystals, in agreement with recent reports.^18,19^ Both the gelling **2a** (DLL, Figure 2B) and the nongelling **4a** (LLD, Figure 2D) assembled into rigid nanotapes, which arose from the alignment of individual nanofibrils with a mean diameter of 1.6 ± 0.2 nm (see Supporting Information, Sections S4 and S5), which was compatible with two or more layers of peptides as shown in Figure 1A for **2a**). Multicopy all-atom MD simulations of DLL **2a** peptides in water indicated the formation of fibrils, thus shedding further light upon the assembly process as described above (see Supporting Information, Section S6).

Notably, **3a** (LDL, Figure 2C) formed spherical nanoparticles that AFM height profiles revealed to be 8 ± 3 nm in diameter (*counts* = 100), with only rare instances of short fibrils of analogous size as **2a**, compatible with two or more layers of peptides (see the Supporting Information, Sections S4 and S5). For comparison, Val-Phe-Phe (LDL stereoisomer) under analogous conditions formed gelling nanotapes that displayed ThT fluorescence.^24^ By contrast, in the case of **3a**, the total absence of ThT fluorescence, which normally arises from the binding of the dye onto fibrils composed of at least four consecutive β-strands, confirmed the disruptive role exerted by Pro (see the Supporting Information, Section S11).^35^

Dynamic light scattering (DLS) was used to investigate the corresponding size distribution of **3a** nanoparticles in solution (see the Supporting Information, Section S7). The hydrodynamic size increased over time; freshly prepared samples consisted of a mixture of smaller (∼18 nm) and larger (∼98 nm) nanoparticles that over 3 h further developed into a monodisperse distribution of 146 nm sized particles (PDI = 0.13).

Time-dependent growth of spherical nanostructures was formerly observed for self-assembling tetrapeptides composed of Pro and Phe.^17^ The discrepancy between DLS and AFM data reflected the differences between wet and dry samples; overall the *in silico* and experimental analyses agreed on the tendency of **3a** toward the formation of stable spherical nanoparticles with uniform distribution of size that could be changed by controlling the hydration level (*i.e.*, wet or dry).

### MD Simulations Elucidated the Divergent Supramolecular Behavior

**Figure 3:**
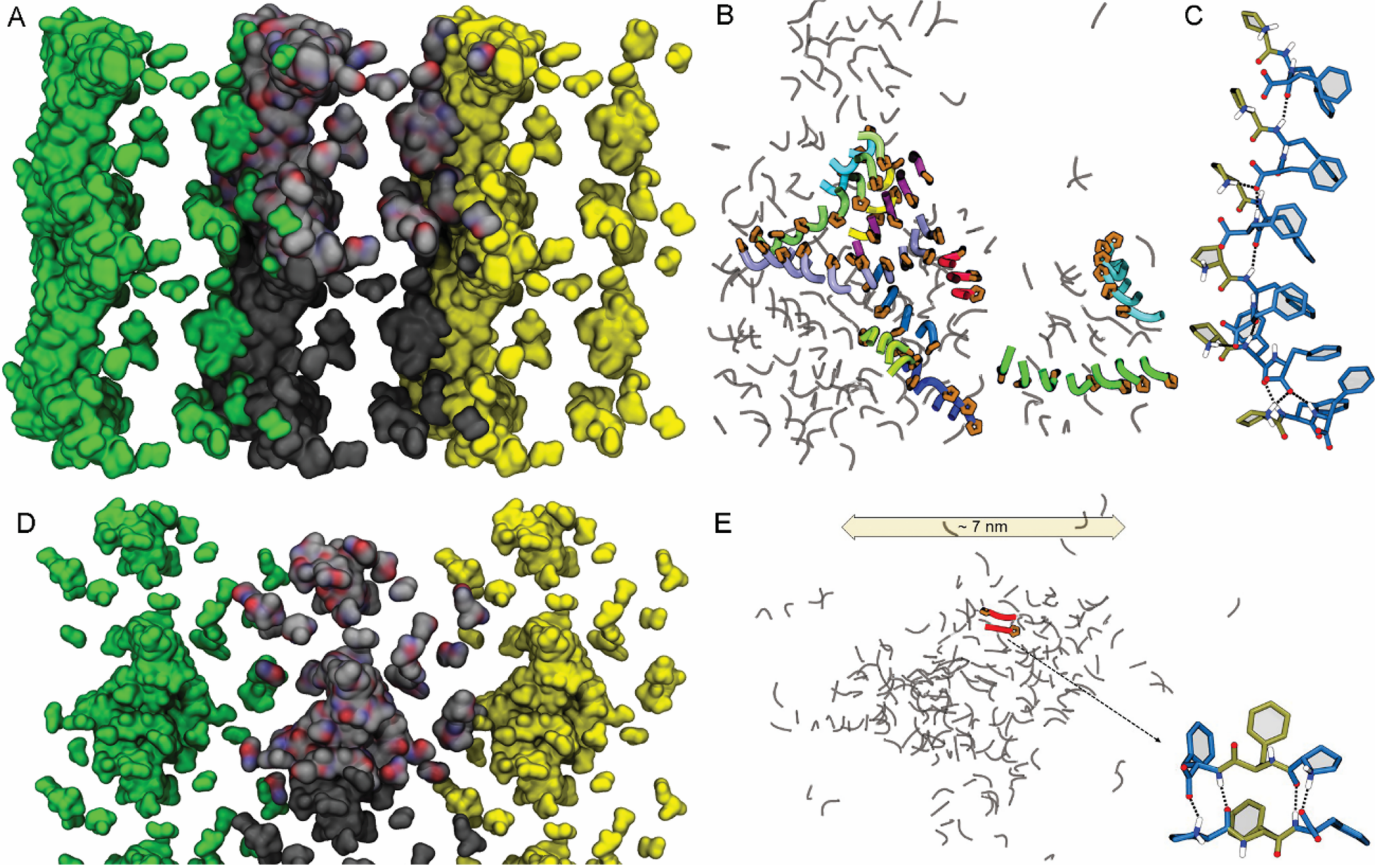
Representative conformations of morphologies of hundreds of **2a** (A–C) and **3a** (D–E) peptides as extracted from multiple all-atom MD simulations extending over 1 μs. (A, D) Assembly of 216 DLL **2a** (A) and **3a** (D) peptides. The surface colored by atom type (C, N, and O in gray, blue, and red, respectively) is the one in the primary simulation box. That in gray extends along the *x* direction, while the two other in yellow or green represent adjacent images. (B) Compound **2a** (DLL) formed fibrils from the association of parallel stacks (colored ribbons with Pro in orange). (C) Amphiphilic parallel stacks of **2a** displaying a continuous network of hydrogen bonds (dashed black lines) between peptide amide groups. (E) **3a** (LDL) self-associated into disordered clusters of ∼7 nm nanoparticles, where hydrogen bonding stabilized mainly antiparallel dimers and trimers.

Multiple all-atom MD simulations of hundreds to 1000 peptides were carried out to shed light on the structural basis of the divergent supramolecular behavior, noted especially for the gelator **2a** and the nanoparticle-forming **3a** (Figure 3). In the case of **2a**, nanofibrils (Figure 3A and the Supporting Information, Section S6) arose from several instances of parallel stacks of peptides. Figure 3B shows a snapshot from the last part of the MD simulation, when sheet-like structures formed by up to ten peptides were clearly visible (shown as ribbons ending with proline in orange, and colored differently). For **2a**, the assembling nature of Phe prevailed over Pro β-breaker, as a continuous network of hydrogen bonds between amides held together parallel stacks (Figure 3C), involving mainly Phe residues. This organization allowed a net segregation between hydrophobic (Phe) and hydrophilic (Pro) residues, so that an amphiphilic supramolecular architecture could arise to allow for higher-order assemblies.^38^ In this manner, Phe residues could face each other to establish dry, steric zippers (as shown in Figure 1A), while Pro residues and the hydrophilic peptide backbones could interact with water to stabilize the hydrogel.

In the case of **3a** (Figure 3D,E), the β-breaker nature of Pro prevailed over the assembling tendency of Phe, as most interactions were limited to antiparallel pairs or triplets of peptides, and no elongated and stable nanostructure arose during the simulations. In the antiparallel arrangement, more hydrogen bonds and salt bridges could be established, often involving Pro residues (Figure 3E). However, as a result, no net segregation between hydrophilic and hydrophobic components persisted, and this could explain the impossibility to establish higher-order assemblies, in contrast with gelling **2a**. Indeed, **3a** dimers and trimers associated disorderly into ∼7 nm wide nanoparticles, in agreement with the absence of ThT fluorescence, and the microscopy and DLS data described above.

MD simulations revealed how and why the fibrillization of Pro-Phe-Phe peptides occurred. When the stereoconfiguration allowed segregation of assembling Phe and β-breaker Pro residues into amphiphilic architectures, the former could zip into higher-order assemblies, whereas the latter could interact with water to gel. On the contrary, when stereoconfiguration impeded the establishment of such supramolecular order, only short-range self-organization persisted, leading to the formation of discrete nanostructures. We anticipate a similar behavior also in the case of the scrambled sequence Phe-Pro-Phe, which displayed an amyloid-like behavior in the case of the homochiral peptide.^18^

### Conformation of Nongelling Tripeptides

**Figure 4:**
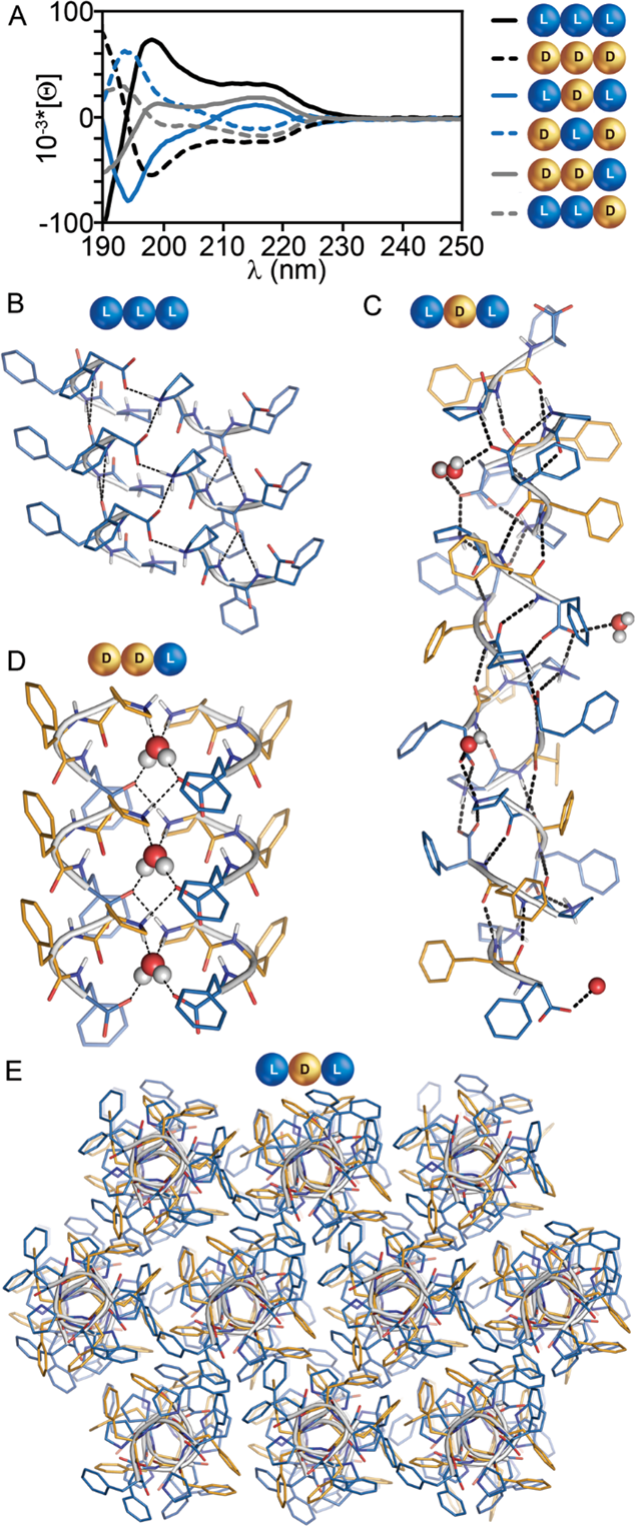
(A) Mirror-image CD spectra of enantiomer pairs **1a**/**1b**, **3a**/**3b**, and **4a**/**4b**. (B–E) Single-crystal XRD structures of **1a** (B), CCDC 2021319, revealing parallel stacks (side view); **3a** ((C) stack side view, (E) stacks top view), CCDC 2026055, revealing a superhelical structure; **4b** (D), CCDC 2021318, revealing parallel stacks of peptide pairs enclosing one hydrogen-bound water molecule.

Enantiomeric pairs displayed mirror-image CD spectra, as expected (Figures 1D and 4A). In particular, the CD signature of homochiral Pro-Phe-Phe (**1a**/**1b**) was very similar to that of heterochiral **2a**/**2b**; we thus inferred an analogous mixture of conformations in solution, of which those with a spectroscopic signature typical of β-structures were the most stable in fresh samples, in agreement with the literature,^38^ and also confirmed by IR spectra (see Supporting Information Section S8).^19^ The CD spectra displayed the same features also in the case of **4a**/**4b**, albeit with lower signal intensity, especially at 198 nm. FT-IR spectra of **4a** were dominated by two maxima at 1624 and 1680 cm^–1^, which typically arise from amyloids and turns, respectively. The presence of ThT fluorescence both in the case of **2a** and **4a** (see Supporting Information, Section S11) also suggested a degree of similarity in the conformations adopted by the two isomers.

Interestingly, the nanoparticle-forming enantiomeric pair **3a**/**3b** showed a more complex CD signal, with sign inversion at 209 nm, reminiscent of β-turns.^42^ The amide I region of the FT-IR spectra was dominated by two maxima at 1687 and 1655 cm^–1^, corresponding to a region in which the characteristic bands of turns appear.^43^ Overall, for all tripeptides the sign of the CD signal in the 210–220 nm range appeared to be dictated by the chirality of the C-terminal amino acid, in agreement with other,^44^ but not all,^24^ reports on short peptides, suggesting the amino acid sequence also played a role.^45^

### Single-Crystal X-ray Diffraction

X-ray diffraction (XRD) analyses of **1a** (CCDC 2021319) and **3a** (CCDC 2026055) revealed that both stereoisomers crystallized in the *P*2_1_ space group. Whereas the crystal structure of **1a** had only one crystallographically independent peptide molecule, **3a** showed a much more complex architecture, with 10 independent molecules in the unit cell. Analysis of the backbone torsion angles of the central residue highlighted a different conformation for the peptides **1a** and **3a**, with a pair of angles analogous to those of α-helices^18^ for **1a** and to those of inverted β-sheets for **3a**, owing to the non-natural chirality of the central residue (see Supporting Information, Section S9). As expected from their different conformation, hydrogen bonding patterns in the crystals were significantly different.

In **1a**, a single three-centered hydrogen bonding interaction connected parallel peptides, forming a stack along the *a* crystallographic direction (Figure 4B). Stacks were held together in the crystal through further hydrophilic interactions, *i.e.*, salt bridges involving the charged C- and N-termini of peptides belonging to different stacks. No solvent molecules were present in the unit cell of the crystal. We infer that the tendency of **1a** to form dry stacks devoid of water drove its precipitation from solution, as described above in the microscopy section.

Interestingly, **3a** revealed an unusual superhelical arrangement responsible for holding tripeptides in amphipathic stacks (side view in Figure 4C), along the *ac* diagonal of the unit cell. In the crystal packing, multiple stacks bundled thanks to hydrophobic interactions between Phe residues at their interface (top view in Figure 4E). In crystals of **3a**, backbone amide groups of each peptide interacted with two neighboring molecules, forming two stronger hydrogen bonds with one of them (O···N distances between 2.8 and 2.9 Å) and two weaker hydrogen bonds with the other (O···N distances between 2.9 and 3.1 Å, Figure 4C). Additional salt-bridge interactions (distances lower than 2.8 Å) were present between neighboring peptides but also between alternate peptides. A careful analysis of the electron density on the N-terminal proline residues confirmed the zwitterionic form for the peptide in the crystal and revealed the presence of water molecules interacting with the charged termini. Thus, water-mediated interactions stabilized peptide–peptide interactions throughout the network (see the Supporting Information, Section S9). The crystallization of **3a** under different experimental conditions (*i.e.*, methanol/water or phosphate buffer) suggested a structural role for these solvent molecules in the formation of the supramolecular organization, as their position was superimposable in the isomorphic structures. We infer that the presence of ordered water molecules every three to four peptides along the stack played a key role in stabilizing long-range assembly, as observed in the crystalline state where water was not mobile. In contrast, in solution, only rare instances occurred of short fibrils as shown by TEM, whose size was compatible with a double layer of peptides that could self-associate through the hydrophobic face (as shown in the view down the stacks in Figure 4E).

Removal of structural water from the crystal would thus destabilize elongated structures that extend beyond three to four peptide molecules, so that mainly short-range assemblies in the form of dimers and trimers would remain, as shown by all-atom MD simulations (Figure 3E). Hydrophobicity-driven self-association into nanoparticles would then proceed in a disordered manner, thus impeding the establishment of long-range assembly as required to form superstructures, which span a length scale that goes well beyond that of their building blocks.

Ordered water molecules served to organize the solid-state structure of **4b** (CCDC 2021318), which crystallized in the *C*2 space group. In this case, one solvent molecule was held between two peptide molecules by strong H-bonds with donor–acceptor distances between 2.65 and 2.75 Å (Figure 4D). Hydrophobic interactions between peptide molecules facilitated the stacking of dimers to generate the observed crystal packing. As in the case of **3a**, the crystal packing was generated by the bundling of stacks only through hydrophobic interactions of the aromatic moieties. However, in contrast with **3a**, water was confined in a hydrophilic region defined by the peptide backbones, thus allowing for a higher level of segregation between hydrophilic and hydrophobic components. We hypothesized that the observed **4a** nanotapes in solution arose from the lateral association of peptide stacks, with an intermediate-range supramolecular assembly relative to **2a** and **3a**. Overall, the three X-ray structures revealed how different amino acid chiralities could be used to fine-tune supramolecular organization.

### Inhibition of Aβ(1–42) Fibrillization

Given the recognized β-breaker character of Pro and the therapeutic avenues offered by the incorporation of d-amino acids in short sequences, the inhibitory activity of the eight stereoisomers toward fibrillization of the amyloid-forming Aβ(1–42) peptide was assessed starting with a ThT fluorescence assay.^46^ On the basis of the structural analysis described above, stereoisomers **3a**/**3b** are inferred to be the best inhibitors, as their stereoconfiguration directed noncovalent interactions so that the disassembling nature of Pro prevailed over the assembling nature of Phe.

Among the eight stereoisomers, **3a** was the best inhibitor, with activity analogous to d-Trp-Aib^32^ that served as a positive control (see Supporting Information Section S10). This dipeptide inhibited the ability of Aβ(1–42) to form toxic assemblies especially when incubated in a large molar excess of 40:1 or more *in vitro*.^32^ When tested on PC12 cells, the toxic effects induced by Aβ(1–42) were reduced with a maximum effect when d-Trp-Aib was used in 10-fold excess relative to Aβ(1–42) over a range of experiments that varied the Aβ(1–42):d-Trp-Aib ratio from 10:1 to 1:10.^32^ The design of this dipeptide aimed at overcoming the limited bioavailability of natural l-peptide inhibitors, such as Leu-Pro-Phe-Phe-Asp that was previously identified as effective inhibitor of Aβ(1–42) fibrillization when used in a 20-fold molar excess, in pioneering work by Soto and colleagues.^47^ Aromatic residues were demonstrated to play a key role in amyloid hydrophobic association and were identified as an effective target to impede formation of toxic oligomers and higher-order assemblies.^48−51^

**Figure 5:**
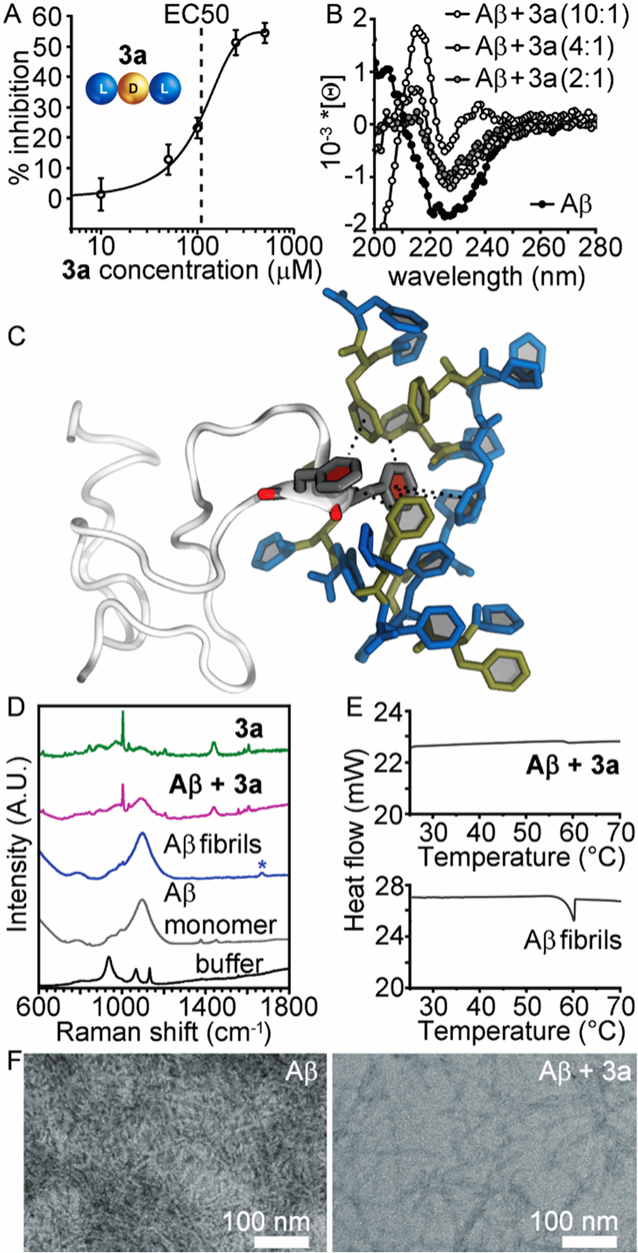
Aβ(1–42) fibrillization inhibition by **3a**. (A) ThT assay dose–response curve (Aβ(1–42) at 10 μM). (B) CD spectra using Aβ(1–42) at 15 μM. (C) Snapshot extracted from a 250 ns all-atom MD simulation of LDL **3a** (l-/d-amino acids in blue/gold) and Aβ(1–42) in gray revealing binding on the Phe-Phe motif as expected by design (interactions shown as dotted black lines). (D) Raman spectra using Aβ(1–42) at 10 μM (* denotes the amyloid diagnostic signal). (E) DSC data (Aβ(1–42) at 100 μM). (F) TEM micrographs.

Dose–response assays revealed that 11:1 **3a** tripeptide molecules per Aβ(1–42) were required to achieve the EC50, and further **3a** concentration increase led to a maximum of 56% inhibition (Figure 5A). It is worth noting that the inhibitor high concentrations were relevant to the experimental protocol for ease of detection, rather than to the physiological levels of Aβ(1–42), or the envisioned therapeutic levels.^32^ These data, which fall within the range of inhibitor:Aβ concentration ratios that proved effective for similar short peptides,^32,47,52^ suggested multiple binding of the inhibitor to Aβ(1–42), as confirmed by CD spectra that showed a reduced β-sheet signature for Aβ(1–42) when **3a** was present in a molar excess (Figure 5B and Supporting Information Section S10). A 250 ns all-atom MD simulation of Aβ(1–42) and 20 molecules of **3a** in water solution revealed up to seven molecules of **3a** binding to the Phe-Phe motif of Aβ(1–42), as expected by design (Figure 5C), and up to 16 binding to a single Aβ(1–42) peptide.

Fibrillization inhibition was also confirmed by Raman spectroscopy, as the diagnostic amyloid signal^53,54^ in the amide I region at 1670 cm^–1^ (denoted with a blue star in Figure 5D) rose with fibrillization but it was absent when Aβ(1–42) was incubated with **3a**. Under analogous conditions, differential scanning calorimetry (DSC, Figure 5E) qualitatively confirmed inhibition,^55^ as the exothermic peak in the thermogram centered at 57.3 ± 2.1 °C was significantly reduced in the presence of **3a**. Nanomorphological analysis supported these findings as TEM micrographs of Aβ(1–42) showed a dense network of amyloid fibrils with widths of 8.6 ± 1.8 nm, in agreement with previous reports,^56,57^ whereas incubation with **3a** led to fewer and shorter fibrils (Figure 5F).

### Enzyme Stability and Biocompatibility of LDL **3a**

**Figure 6:**
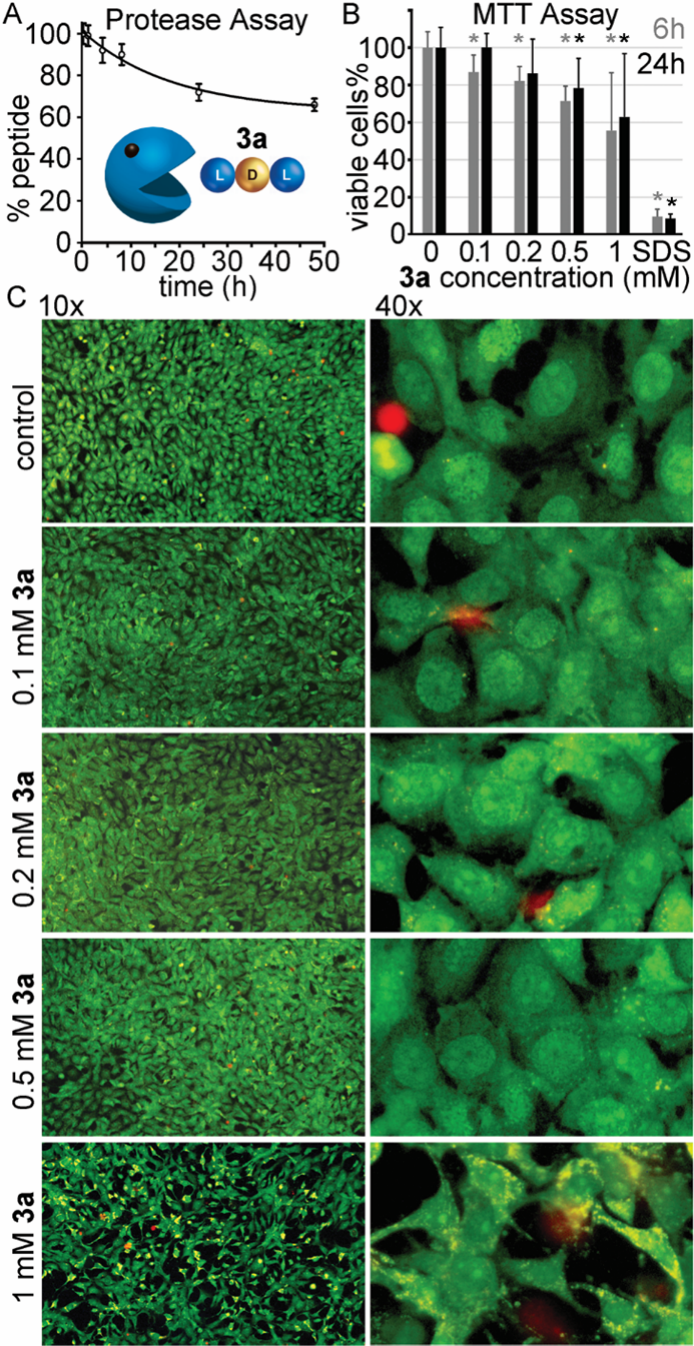
(A) Protease-digestion assay for **3a**. (B) MTT assay for **3a** on keratinocytes. * denotes *p* < 0.05 (*t* test). (C) Live–dead fluorescence microscopy images of fibroblasts cultured in the presence of increasing concentrations of **3a**.

Another critical aspect for the development of amyloidosis inhibitors is the rapid enzymatic processing *in vivo* undergone by natural l-peptides. Therefore, we tested the stability of **3a** when incubated with a large excess of proteinase K, with l-homochiral **1a** serving as a control. The latter was quantitatively hydrolyzed already after 1 h of incubation while only 34% of LDL **3a** was digested after 48 h (Figure 6A). These results confirmed the stabilizing effect against proteases of even one d-amino acid strategically positioned at the center of the tripeptide sequence, thus allowing us to address one of the main limitations of peptide-based therapeutics. However, the limited enzyme-processability of heterochiral sequences may also raise concerns over their biocompatibility. To allay these concerns, keratinocytes or fibroblast cells were cultured in the presence of increasing concentrations of **3a**, and cell viability was assessed by MTT or live/dead assays (Figure 6B,C). Only at the highest concentration tested were evident signs of cytotoxicity noted, with low cell viability, density, and altered morphology, as cells appeared more elongated with reduced volume. All amyloidosis inhibition tests were performed at lower concentrations in the micromolar range, however, at which no toxicity was detected in cell culture. Furthermore, the vast majority of xenobiotics are expected to exhibit side effects at millimolar concentrations. Overall, these data suggest that heterochiral short peptides are a promising starting point for the development of amyloid inhibitors.

## Conclusions

The presence of proline affects, but does not impede, assembly of unprotected tripeptides based on the Phe-Phe motif. With the additional variable of amino acid stereoconfiguration, it is indeed possible to direct the self-organization of Pro-Phe-Phe peptides that contain conflicting assembly instructions, so as to achieve a diversity of discrete nanostructures, such as nanotapes or nanoparticles, or even to obtain a self-supporting hydrogel. Furthermore, this soft material undergoes a gel-to-sol transition at a physiologically relevant temperature, which is convenient for the future development of smart biomaterials. Single-crystal XRD data and all-atom MD simulations revealed fine details of supramolecular packing for all of the peptides studied and were in agreement with spectroscopy and microscopy data, thus significantly advancing the understanding of these systems for their future application in biomedical nanotechnology.

The presence of divergent programming elements in a peptide sequence as short as three amino acids thus offers the opportunity to direct supramolecular behavior, based on the strategic use of heterochirality. When self-assembly prevails, long-range assembly can be achieved for fibrillization to occur and superstructures can organize effectively into a network holding together a macroscopic hydrogel, as demonstrated with peptides **2a**/**2b**. When disassembly prevails, only short-range assembly is attained, and the disruptive nature of Pro can be exploited to extend it on fibrillating biological targets, as demonstrated on Alzheimer’s related Aβ(1–42) *in vitro* and shown effectively for **3a**. Finally, a balance between the divergent effects can stabilize intermediate-range assembly, as noted for the nanotape-forming **4a**/**4b**.

Homochirality thus represents a fundamental principle that governs the structure and function of living organisms, allowing heterochirality to serve as a strategic tool for the design of orthogonal systems to achieve structures and functions at the interface with living beings. This work has shown how the combination of simple elements with divergent supramolecular behaviors can be an effective approach to achieve structural and functional diversity for the design of biocompatible soft materials and therapeutics, allowing avenues to be envisaged for a broader scope of applications, spanning from artificial enzymes to innovative approaches directing protein-mediated cell fate.

## Materials and Methods

### Chemical Synthesis

2-Chlorotrytil and Fmoc-phenylalanine preloaded resin, *O*-benzotriazole-*N*,*N*,*N*,*N*′-tetramethyluronium hexafluorophosphate (HBTU), 1-hydroxy-7-azabenzotriazole (HOAt), and Fmoc-d-phenylalanine were purchased from GL Biochem (Shanghai) Ltd. All of the other chemicals and solvents of analytical grade were purchased from Merck. Tripeptides were synthesized on solid phase according to established procedures.^34^ The crude oils were purified by reverse-phase HPLC on an Agilent 1260 Infinity system equipped with a gradient pump, C-18 column (Kinetex, 5 μm, 100 Å, 250 × 10 mm, Phenomenex), autosampler, and photodiode array detector. The gradient used consisted of acetonitrile (MeCN)/water with 0.1% TFA with the following program: *t* = 0–2 min, 25% MeCN; *t* = 16 min, 95% MeCN; *t* = 16–18 min, 95% MeCN (*R*_*t*_ = 10–12 min). The compounds were then freeze-dried and characterized by ESI-MS, ^1^H NMR, and ^13^C NMR analyses. ^1^H NMR spectra were recorded at 400 MHz and ^13^C NMR spectra were recorded at 100 MHz on a Varian Innova Instrument with chemical shift reported as ppm (in DMSO or acetonitrile with tetramethylsilane as internal standard). LC-MS data were acquired on an Agilent 6120 LC-MS system with a C-18 analytical column (Zorbax SB-C18 Rapid Resolution HT 2.1 mm × 50 mm, 1.8 μm) with a single quadrupole ESI system. Flow was 0.5 mL/min. The gradient used consisted of acetonitrile (MeCN)/water with 0.1% formic acid with the following program: *t* = 0–2 min, 5% MeCN; *t* = 20 min, 95% MeCN (+0.1% formic acid). Spectroscopic data and HPLC retention times are reported in the Supporting Information, Sections S1 and S2.

### Self-Assembly of Tripeptides

Each peptide was dissolved in phosphate buffer (sodium phosphate, 0.1 M; pH ∼ 11.9) by sonication at room temperature; then the solution was diluted with an equal volume of phosphate buffer (0.1 M sodium phosphate; pH ∼ 5.9) to reach pH neutrality. Self-assembly of peptide zwitterions thus occurred at physiological pH 7.4 and a concentration of 20 mM (except for the hydrogel at 24 mM). *Note: complete dissolution of the peptide and fine pH control are important to achieve correct hydrogelation.*

### TEM

TEM micrographs were acquired on a Jeol instrument, JEM 2100, Japan, at 100 kV. TEM grids (copper-grid-supported lacey carbon film) were first rendered hydrophilic by a 20 min treatment in a UV–ozone cleaner (UV-Ozone Procleaner Plus). After 1 h of self-assembly, sample aliquots (∼20 μL) were deposited on top of a copper grid for 30 s (200 mesh, copper, carbon only or lacey carbon). Next, water was drawn from the sample and the grid was placed for 30 s in contact onto a drop of 2% aqueous potassium phosphotungstate at pH 7.2. Finally, the grid was dried first at room temperature and then *in vacuo*. Image analysis was performed using ImageJ software.

### AFM

Samples were spread on a 1 cm^2^ mica substrate, and after 1 h of self-assembly, they were dried *in vacuo* for 24 h. AFM analysis was carried out on a Nanoscope V microscope (Digital Instruments Metrology Group, model MMAFMLN) in tapping mode at room temperature in air, using a standard μmasch SPM probe (HQ:NSC15/AIBS) with tip height of 12–18 μm and a cone angle < 40° (resonant frequency, 320 kHz; force constant ∼ 40 N m^–1^). Gwyddion software was used for image analysis.

### DLS

The hydrodynamic size distributions were measured on a Fritsch, Analysette 12 DynaSizer (Germany) supported with NanoQ V2.5 software (Cordouan Technologies). DLS is equipped with variable laser power (from 1 to 65 mW) operating at 657 nm and a scattering detector at 135°. The peptide powder was dissolved in corresponding buffers immediately before measurements. A quantity of 50 μL of nondiluted sample was measured in an ultrathin layer (20 μm) to avoid multiple scattering events. All measurements were performed in triplicate at room temperature (25 °C).

### Rheology

Hydrogel **2a** was measured on a Malvern Kinexus Ultra Plus Rheometer (Alfatest) with a 2 cm steel parallel plate at 25 °C (Peltier, Alfatest). Samples were prepared *in situ* and analyzed with a gap of 1 mm. Time sweeps were recorded for 1 h at 1 Hz and 1 Pa; frequency sweeps were recorded at 1 Pa and stress sweeps at 1 Hz. Each analysis was repeated at least 3 times, and representative measures are reported in the Supporting Information, Section S3.

### Circular Dichroism

Analyses were carried out in a 0.1 mm quartz cell using a Jasco J-815 spectropolarimeter, with 1 s integrations, step size of 1 nm, bandwidth of 1 nm, from 185 to 280 nm at 25 °C. Samples were prepared directly in the cuvette. For the hydrogel tripeptide **2a**, a temperature ramp up to 85 °C was applied after 1 h of gelation. Aβ(1–42) was monomerized for 2 h in 1,1,1,3,3,3-hexafluoro-2-propanol (HFIP, 500 μM); then, HFIP was evaporated under an argon flow and Aβ left overnight under vacuum. Each sample contained 15 μM Aβ(1–42) and the tripeptide **3a** at different concentrations (2:1, 4:1, or 10:1 relative to Aβ) in PBS 20 mM (pH 7.4). Controls containing only 15 μM Aβ(1–42) and tripeptide **3a** at different concentrations were also measured. Samples were incubated for 48 h in a thermomixer (150 rpm) at 37 °C; spectra were recorded for freshly prepared samples and after 24 and 48 h of incubation. Two independent experiments were performed. The data shown are the average of the five accumulations. Further data are shown in the Supporting Information, Section S10.

### FT-IR

Spectra were recorded on a Varian 660-IR spectrometer in ATR mode (Ge crystal) with 80 scans, 4 cm^–1^ resolution. Each sample was spread onto a clean piece (∼1 cm^2^) of silicon wafer, and after 1 h of self-assembly it was dried *in vacuo* for 24 h.

### Crystallization

Single crystals were grown by dissolving each peptide in 0.8 mL of MeOH inside a reservoir with 3 mL of a mixture of methanol and water. Peptides **1a**/**1b** and **3a**/**3b** were dissolved at 2.5 mM in methanol and the reservoir contained methanol/water (40:60). Peptides **4a**/**4b** were dissolved at 1 mM methanol, and the reservoir contained methanol/water (50:50). Peptides **3a**/**3b** also crystallized, dissolved at 10 mM in 10 mM PBS over a month.

### Single-Crystal X-ray Diffraction

Single crystals of a single enantiomer for each crystallized pair were analyzed by X-ray diffraction at the XRD1 beamline of the Elettra synchrotron (Trieste, Italy). Crystals were collected from their mother solution using a nylon loop and stored in liquid nitrogen, with poly(ethylene glycol) (for **1a** and **3a**) or glycerol (for **4b**) as cryoprotectant. Data collections were performed on the frozen crystals, kept at 100 K by a stream of nitrogen, with the rotating crystal technique. Data were indexed and integrated using either the software Mosflm^58^ (for **1a** and **3a**) or the XDS package^59^ (for **4b**), and scaled using AIMLESS software.^60^ For all structures the phase problem was solved using direct methods, with software SHELX-T^61^ (for **1a**), SIR 2014^62^ (for **3a**), or SHELXS^63^ (for **4b**). Refinement cycles were conducted with SHELXL-14,^64^ by full matrix least-squares methods on F^2^.

### Thioflavin T Test

ThT assay on the tripeptides was performed as previously described, using a concentration for each peptide of 20 mM, or 24 mM for **2a**/**2b**, and 6 and 24 h as time points for self-assembly.^26^ For the Aβ(1–42) fibrillization inhibition studies, Aβ(1–42) was monomerized for 2 h in HFIP (0.5 mM); then HFIP was evaporated under an argon flow and left overnight under vacuum. Then, it was dissolved in DMSO at 0.3 mM and stored at −20 °C. Tripeptide solutions were prepared at 10 mM in DMSO and diluted to 2 mM with PBS 10 mM, being the final concentration in the plates, 0.2 mM. Thioflavin T solution was prepared at 25 μM in glycine–NaOH buffer (pH 8.5). The experiment was performed in a 96-well flat black clear-bottom plate (final Aβ(1–42) concentration of 10 μM and final volume per well = 150 μL). The plate was incubated at 37 °C with a shaking speed of 150 rpm, covered with aluminum foil. The bottom fluorescence was measured using a Tecan Infinite M1000 Pro, excitation 446 nm and emission 490 nm, respectively, for freshly prepared samples and after 24 and 48 h of incubation. Controls contained only Aβ(1–42), only tripeptides, or d-Trp-Aib. A sample containing only ThT was used as reference. Three independent experiments were carried out in triplicate.

### Raman Spectroscopy

Raman spectra were recorded with an Invia Renishaw microspectrometer (50) equipped with He–Ne laser at 532 nm. At least 25 spectra (resolution 1 cm^–1^) per sample were collected to assess their homogeneity. Compound **3a** (0.2 mM) was added 20:1 to Aβ(1–42) for 24 h, placed atop a 1 cm^2^ piece of a silicon wafer, and then dried *in vacuo* prior to analysis.

### DSC

Aβ(1–42) was dissolved in 0.1 M aqueous ammonia solution at 1 mg/mL. Then, it was diluted with PBS 10 mM to 100 μM, with or without **3a** (2 mM in PBS 10 mM), and samples were incubated at 37 °C for 48 h. Data were acquired on a Q100 (TA Instruments). For every measurement, 50 μL of each sample was transferred to the DSC pan and they were hermetically closed with their lids. DSC scans were performed using the following program: (1) isotherm at 20 °C for 1 min; (2) heating ramp from 20 to 80 °C at 5 °C/min; (c) isotherm at 80 °C for 5 min; (d) cooling to 20 °C at 5 °C/min. Measurements were repeated at least in triplicates for each independent sample.

### Protease Assay

A 0.25 mL aliquot of peptide solution in DMSO (10 mM) was prepared from each peptide in 15 mL Falcon tubes, and 4.75 mL of 50 mM sodium phosphate buffer (pH 7.4) with a large excess (5 mg) of recombinant proteinase K (Sigma-Aldrich) was added. The tubes were incubated at 37 °C, 60 rpm, and at the selected time points, 0.2 mL of 1 M NaOH was added to 0.5 mL of the solution to inhibit further protease activity prior to HPLC analysis. The experiment was carried out twice in triplicate.

### Molecular Models

Model structures of zwitterionic tripeptides were built upon the experimental structure (**3a**) or generated using the AmberTools19 package (D. A. Case *et al.* AMBER 2019 (University of California, San Francisco)) and the VMD1.9.3 software^65^ through in-house *tcl* scripts (**2a**). Multicopy MD simulations of the self-assembly process for 216 **2a** or **3a** tripeptides were performed as previously described^38^ (see Supporting Information Section S6). To further investigate the molecular determinants of **2a** fibrillization, an additional set of simulations was performed with 1,000 such peptides. The initial structures were generated by placing the center of mass of one peptide repeatedly on the points of a 10 × 10 × 10 (**2a**) or 6 × 6 × 6 (**2a**/**3a**) grid of 15 Å spaced points. Initial orientations of peptides were randomized, and the systems were solvated with water molecules (concentration ∼0.25 M). To investigate the molecular interactions occurring in solvent between **3a** and Aβ(1–42), we performed a MD simulation of Aβ(1–42)^66^ interacting with 20 peptides in water solution. See Supporting Information Section S6 for further details.

### Cell Culture

A 0.2 μm filtration setup was used to sterilize all solutions. MTT assays were performed as previously described.^26^ For live/dead assays, NIH3T3 fibroblasts (10,000 cells per well, 30 μL of DMEM + 10% fetal serum albumin, and 2% antimycotic and antibiotic from GIBCO) were added to the microwells of an uncoated μ-Slide for Angiogenesis (Ibidi, Germany) and cultured at 37 °C, 5% CO_2_ for 24 h, according to the manufacturers’ instructions. A stock solution of **3a** (4 mg/mL in 10 mM PBS, pH 7.4) was diluted to the desired concentration (0.1, 0.2, 0.5, or 1 mM) and added to the wells. After 24 h, cell viability was assayed with acridine orange (5 μL/well of a 20 μM solution in 50 mM PBS) and propidium iodide (4 μL/well of a 30 μM solution in 50 mM PBS). After 15 min at 37 °C, cells were imaged with a Leica microscope (DFC450C; *software LASV4.13*) with fluorescence green filter (excitation, 450–490 nm; emission > 520 nm) with 10× and 40× objectives. Each condition was repeated in triplicate and the experiment twice. The *t* test was performed with Excel by comparing each condition against its control without peptide.

## Supporting Information Available

Spectroscopic characterization, rheology, microscopy, DLS, XRD data, modeling details, and the ThT assay on all the peptides (PDF)
Movie S1 showing **2a** parallel stacks reminiscent of β-sheets (MP4)
