# Supplementary material for: Nanoscale Assembly of Functional Peptides with Divergent Programming Elements
Source: ACS Nano. 2021 Feb 12;15(2):3015–25. doi: 10.1021/acsnano.0c09386 (PMC8023796; doi:10.1021/acsnano.0c09386)
Supplement: Supplementary file 1 — nn0c09386_si_001.pdf [file nn0c09386_si_001.pdf]

Supporting Information for:

Nanoscale Assembly of Functional Peptides with Divergent Programming Elements

Ana M. Garcia,[‡] *Michele Melchionna,*[‡,†] *Ottavia Bellotto,*[‡] *Slavko Kralj,*[‡,¶] *Sabrina Semeraro,*[‡] *Evelina Parisi,*[‡] *Daniel Iglesias,*[‡] *Paola D'Andrea,*[‖] *Rita De Zorzi,*[‡] *Attilio V. Vargiu,*[⌈*] *and Silvia Marchesan.*[‡,†*]

‡ Chem. Pharm. Sc. Dept., University of Trieste, Via Giorgieri 1, 34127 Trieste, Italy
† INSTM, Unit of Trieste, Via Giorgieri 1, 34127 Trieste, Italy
¶ Materials Synthesis Dept., Jožef Stefan Institute, Jamova 39, 1000 Ljubljana, Slovenia
‖ Life Sciences Dept., University of Trieste, Via Giorgieri 1, 34127 Trieste, Italy
⌈ Physics Dept., University of Cagliari, s.p. 8, km. 0.700, 09042 Monserrato, Italy

*e-mail: vargiu@dsf.unica.it, smarchesan@units.it

S1. Spectroscopic data……………………..……………………………………. S2

S2. HPLC traces……………………..……………………………………………… S18

S3. Rheology data for **2a** and **2b** and photographs of **2b**………………………… S19

S4. TEM micrographs…………………………………………………………………. S19

S5. AFM images………………………………………………………………………. S24

S6. Full-atom MD simulations of tripeptides………………………………………. S28

S7. DLS data……………………………………………………………………….. S30

S8. FT-IR spectra……………………………………………………………………. S31

S9. XRD data………………………………………………………………………….. S31

S10. Aβ(1-42) inhibition data………………………………………………………. S38

S11. Thioflavin T fluorescence on tripeptides……………………………………… S39

S1. Spectroscopic data (NMR and ESI-MS)

1a: L-Pro-L-Phe-L-Phe

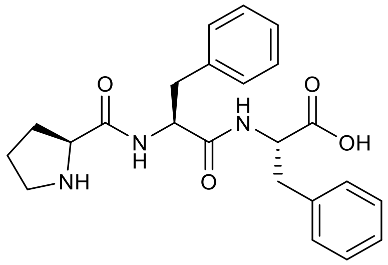

Fig. S1. Chemical structure of **1a**.

^{1}H NMR (500 MHz, DMSO-d_6) δ 8.68 (d, J = 8.5 Hz, 1H, NH), 8.46 (d, J = 7.8 Hz, 1H, NH), 7.36 – 6.93 (m, 10H, Ar), 4.58 (ddd, J = 9.7, 4.2 Hz, 1H, αCH), 4.44 (ddd, J = 8.5, 5.2 Hz, 1H, αCH), 4.09 – 3.94 (m, 1H, αCH), 3.21 – 2.97 (m, 4H, 2 x ProCH$_2$, 2 x βCH$_2$), 2.91 (dd, J = 13.9, 9.2 Hz, 1H, βCH$_2$), 2.72 (dd, J = 13.9, 10.2 Hz, 1H, βCH$_2$), 2.25 – 2.12 (m, 1H, ProCH$_2$), 1.92 – 1.64 (m, 3H, ProCH$_2$). **^{13}C NMR** (125 MHz, DMSO-d_6) δ 173.1, 171.1, 168.4 (3 x CO); 137.9, 137.8, 129.6, 129.5, 128.6, 128.5, 126.9 (Ar); 59.2, 54.6, 54.0 (3 x αC); 46.2 (C-Pro); 37.9, 37.1 (2 x βC), 30.0, 23.9 (2 x C-Pro). **MS (ESI)** m/z 410.1 $(M+H)^+$ $C_{23}H_{27}N_3O_4$ requires 409.2.

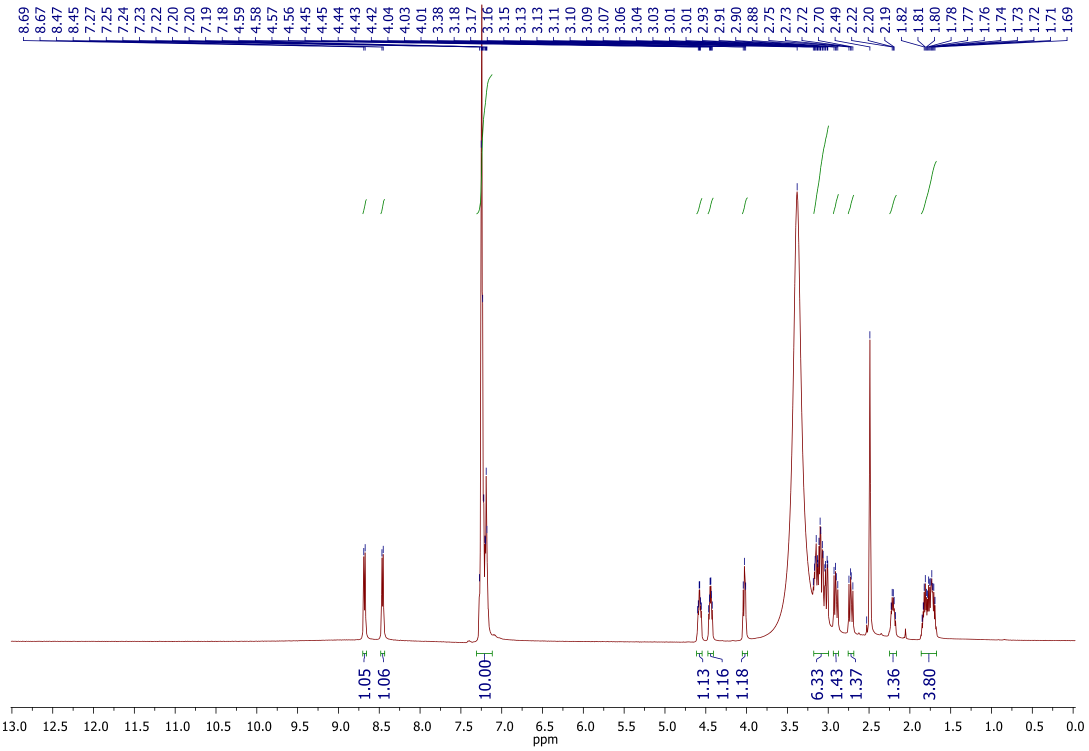

Fig. S2. ^{1}H-NMR spectrum of **1a**.

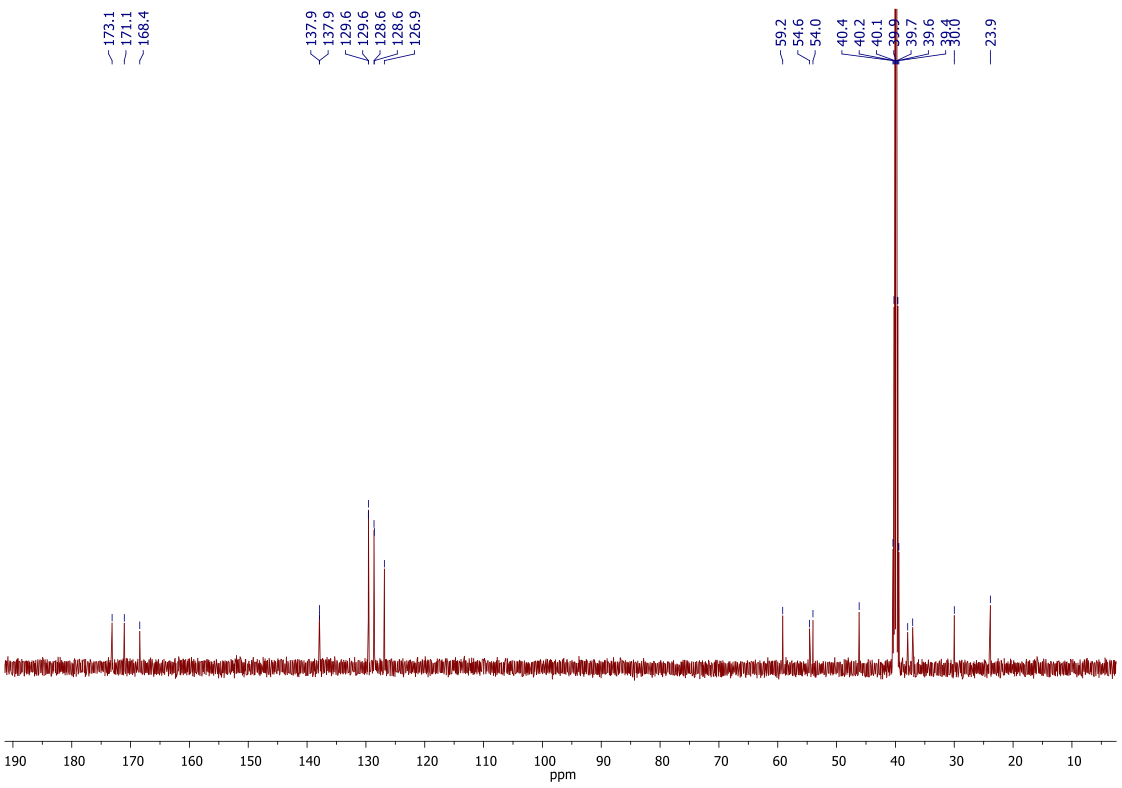

Fig. S3. ^{13}C-NMR spectrum of **1a**.

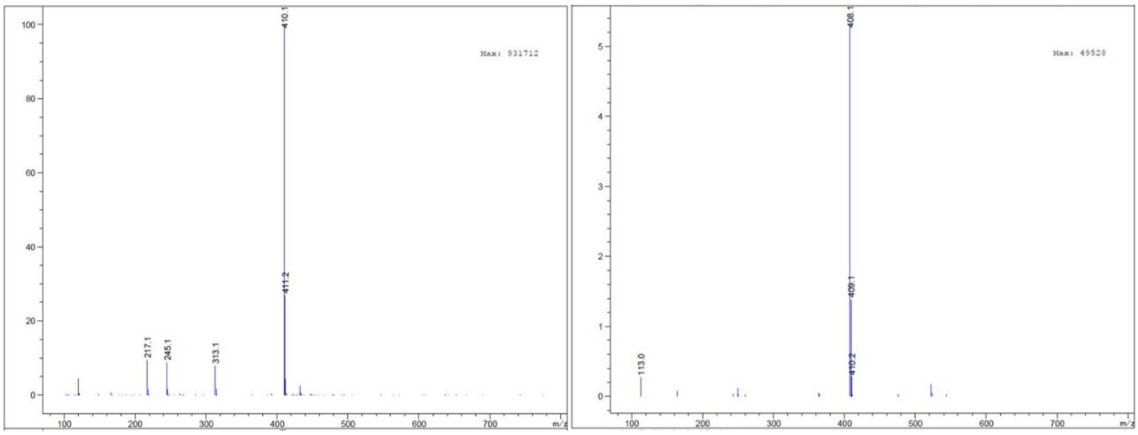

Fig. S4. ESI-MS spectra of **1a** in the positive (left) and negative (right) ion mode.

1b: D-Pro-D-Phe-D-Phe

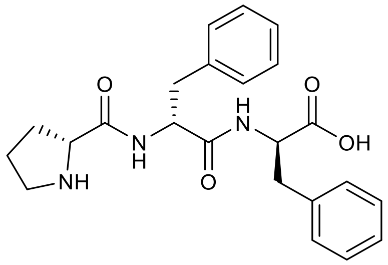

Fig. S5. Chemical structure of **1b**.

^{1}H NMR (500 MHz, DMSO-d_6) δ 8.70 (d, J = 8.5 Hz, 1H, NH), 8.47 (d, J = 7.9 Hz, 1H, NH), 7.38 – 7.02 (m, 10H, Ar), 4.72 – 4.51 (m, 1H, αCH), 4.45 (ddd, J = 8.9, 5.0 Hz, 1H, αCH), 4.04 (ddd, J = 8.5, 6.6 Hz, 1H, αCH), 3.21 – 2.98 (m, 3H, 2 x ProCH$_2$, 2 x βCH$_2$), 2.91 (dd, J = 13.9, 9.2 Hz, 1H, βCH$_2$), 2.73 (dd, J = 13.9, 10.1 Hz, 1H, βCH$_2$), 2.26 – 2.16 (m, 1H, ProCH$_2$), 1.90 – 1.66 (m, 3H, ProCH$_2$). **^{13}C NMR** (125 MHz, DMSO-d_6) δ 173.1, 171.1, 168.4 (3 x CO); 137.9, 137.9, 129.6, 129.5, 128.6, 128.5, 126.9 (Ar); 59.1, 54.6, 54.0 (3 x αC); 46.2 (C-Pro); 37.9, 37.1 (2 x βC); 30.0, 23.9 (2 x C-Pro). **MS (ESI)** m/z 410.2 (M+H)$^+$ $C_{23}H_{27}N_3O_4$ requires 409.2.

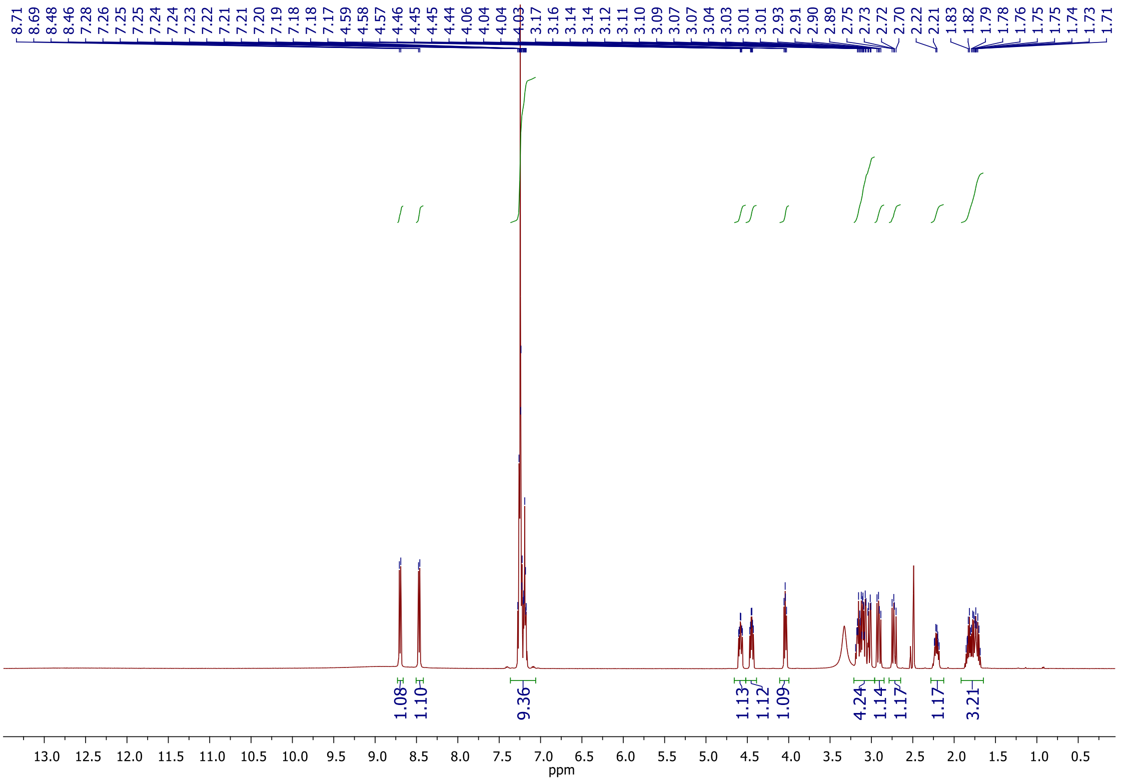

Fig. S6. ^{1}H-NMR spectrum of **1b**.

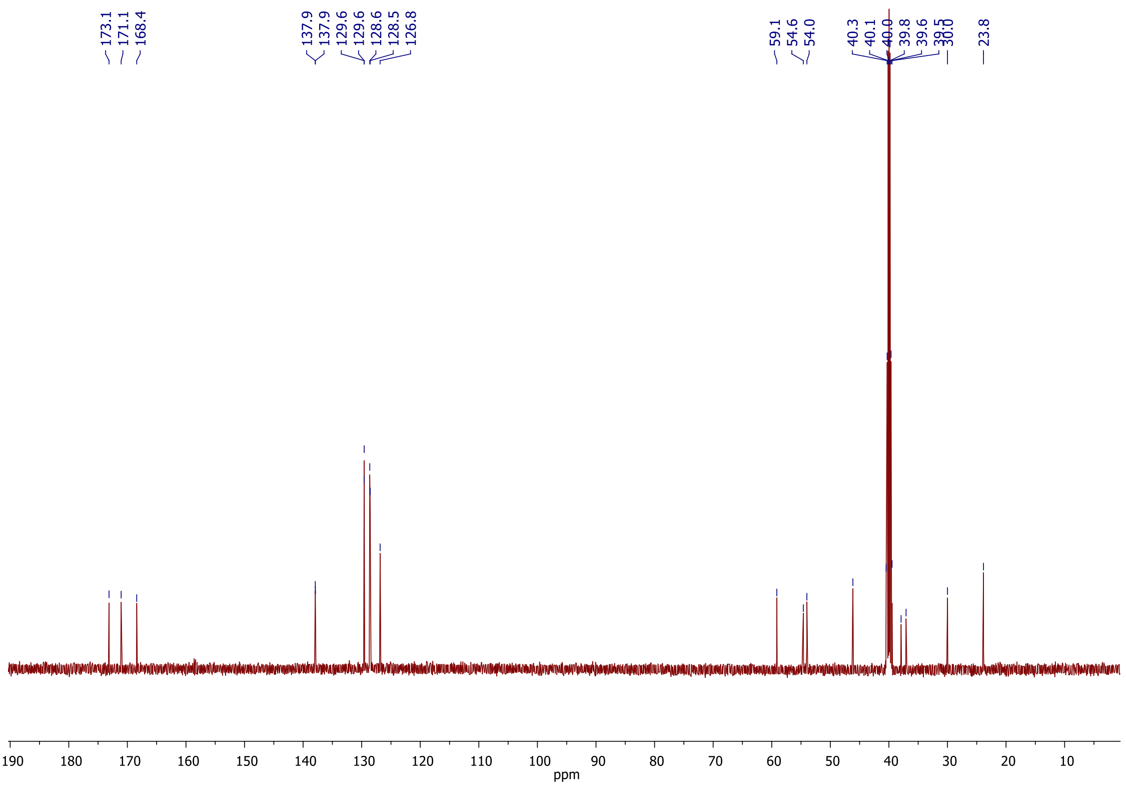

Fig. S7. ^{13}C-NMR spectrum of **1b**.

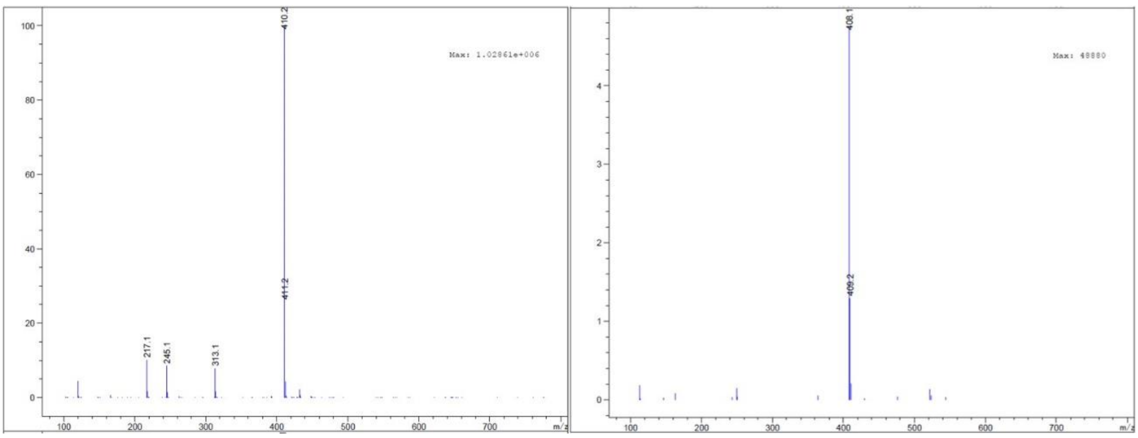

Fig. S8. ESI-MS spectra of **1b** in the positive (left) and negative (right) ion mode.

2a: D-Pro-L-Phe-L-Phe

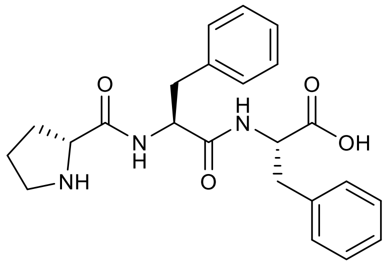

Fig. S9. Chemical structure of **2a**.

^{1}H NMR (500 MHz, DMSO-*d*$_6$) δ 8.72 (d, *J* = 9.0 Hz, 1H, NH), 8.58 (d, *J* = 7.9 Hz, 1H, NH), 7.37 – 7.11 (m, 10H, Ar), 4.70 (ddd, *J* = 10.9, 9.3, 3.7 Hz, 1H, αCH), 4.46 (ddd, *J* = 9.0, 5.0 Hz, 1H, αCH), 4.04 (ddd, *J* = 7.6 Hz, 1H, αCH), 3.14 – 3.00 (m, 4H, 2 x ProCH$_2$, 2 x βCH$_2$), 2.92 (dd, *J* = 14.0, 9.3 Hz, 1H, βCH$_2$), 2.65 (dd, *J* = 13.7, 11.0 Hz, 1H, βCH$_2$), 2.04 (ddd, *J* = 14.4, 7.9 Hz, 1H, ProCH$_2$), 1.71 (m, *J* = 14.3, 7.3 Hz, 1H, ProCH$_2$), 1.60 – 1.41 (m, 1H, ProCH$_2$), 1.21 (ddd, *J* = 14.6, 7.2 Hz, 1H, ProCH$_2$). **^{13}C NMR** (125 MHz, DMSO-*d*$_6$) δ 173.2, 171.2, 168.0 (3 x CO); 138.0, 137.8, 129.8, 129.5, 128.7, 128.4, 126.9, 126.8 (Ar); 59.4, 54.1, 53.9 (3 x αC); 45.9 (C-Pro); 38.6, 37.0 (2 x βC); 30.9, 23.6 (2 x C-Pro). **MS (ESI)** m/z 410.2 $(M+H)^{+}$ $C_{23}H_{27}N_3O_4$ requires 409.2.

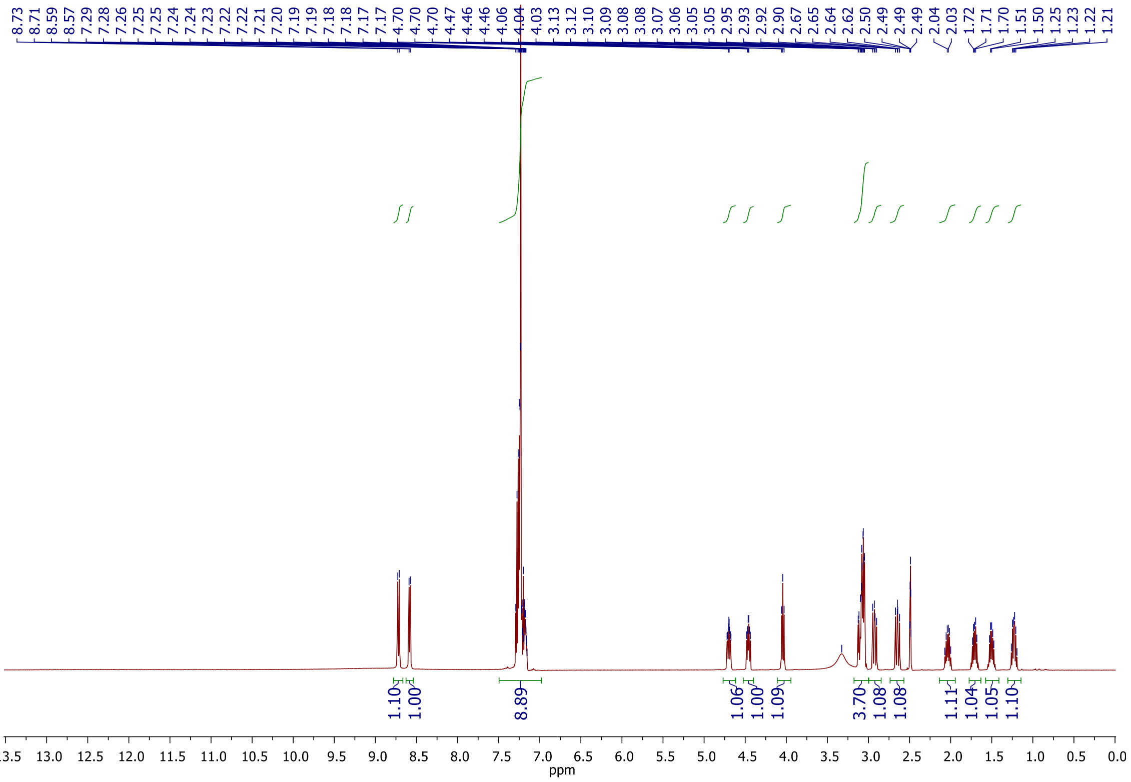

Fig. S10. ^{1}H-NMR spectrum of **2a**.

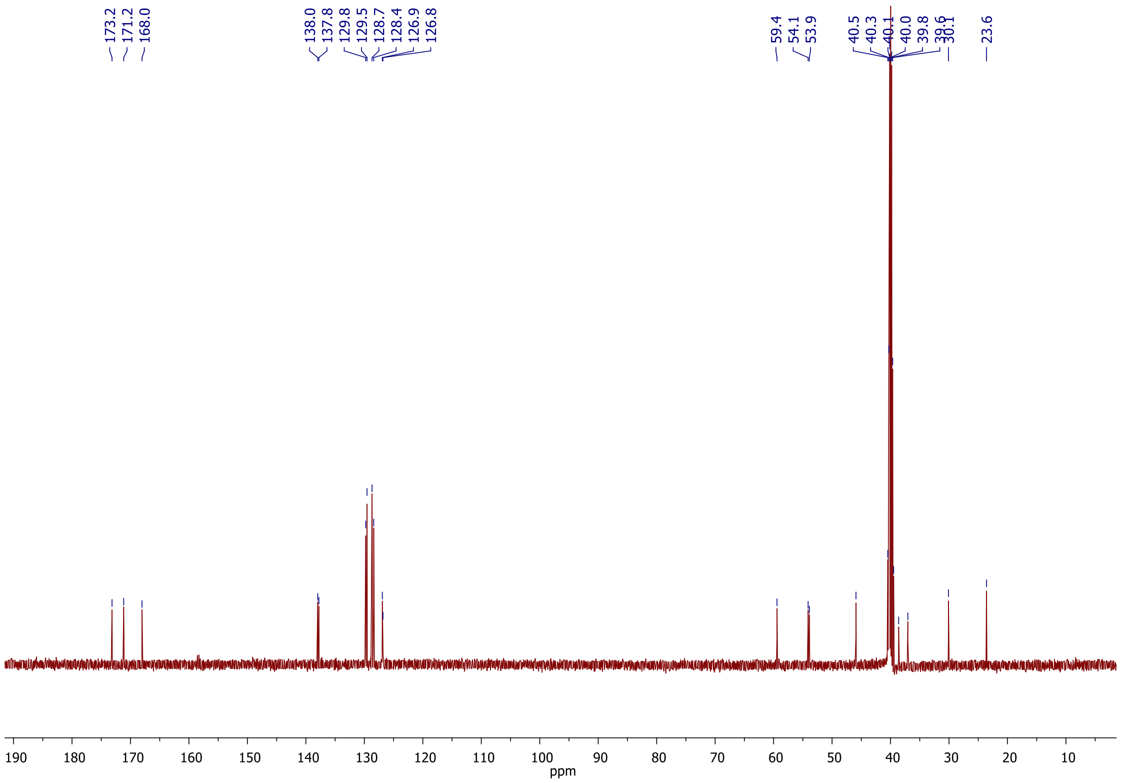

Fig. S11. ^{13}C-NMR spectrum of **2a**.

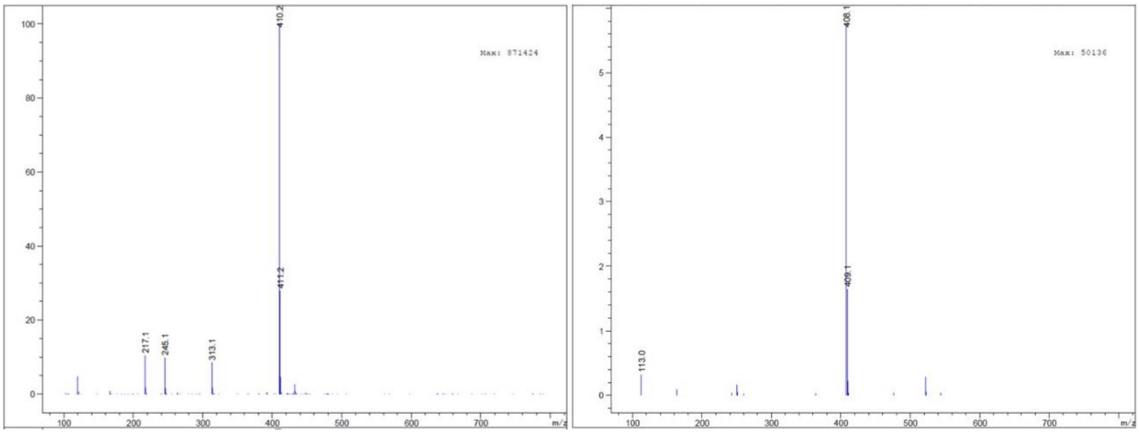

Fig. S12. ESI-MS spectra of **2a** in the positive (left) and negative (right) ion mode.

2b: L-Pro-D-Phe-D-Phe

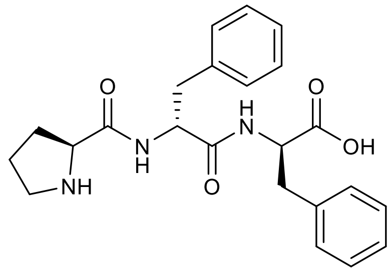

Fig. S13. Chemical structure of **2b**.

^{1}H NMR (500 MHz, DMSO-d_6) δ 12.87 (s, 1H, OH), 8.73 (d, J = 9.0 Hz, 1H, NH), 8.61 (d, J = 7.9 Hz, 1H, NH), 7.55 – 7.04 (m, 10H, Ar), 4.71 (ddd, J = 10.9, 9.0, 3.7 Hz, 1H, αCH), 4.48 (ddd, J = 9.2, 7.8, 5.0 Hz, 1H , αCH), 4.06 (dd, J = 7.6 Hz, 1H, αCH), 3.16 – 3.01 (m, 4H, 2 x βCH_2, ProCH_2) 2.94 (dd, J = 14.0, 9.3 Hz, 1H, βCH_2), 2.66 (dd, J = 13.7, 10.9 Hz, 1H, βCH_2), 2.05 (m, J = 12.9, 8.0, 6.3 Hz, 1H, ProCH_2), 1.83 – 1.63 (m, 1H, ProCH_2), 1.51 (m, J = 14.1, 7.3 Hz, 1H, ProCH_2), 1.24 (m, J = 13.3, 7.2 Hz, 1H, ProCH_2). **^{13}C NMR** (125 MHz, DMSO-d_6) δ 173.1, 171.2, 167.9 (3 x CO); 137.9, 137.8, 129.8, 129.5, 128.7, 128.4, 126.9, 126.8 (Ar); 59.4, 54.1, 53.9 (3 x αC); 45.9 (C-Pro); 38.6, 37.0 (2 x βC); 30.1, 23.5 (2 x C-Pro). **MS (ESI)** m/z 410.2 $(M+H)^+$ $C_{23}H_{27}N_3O_4$ requires 409.2.

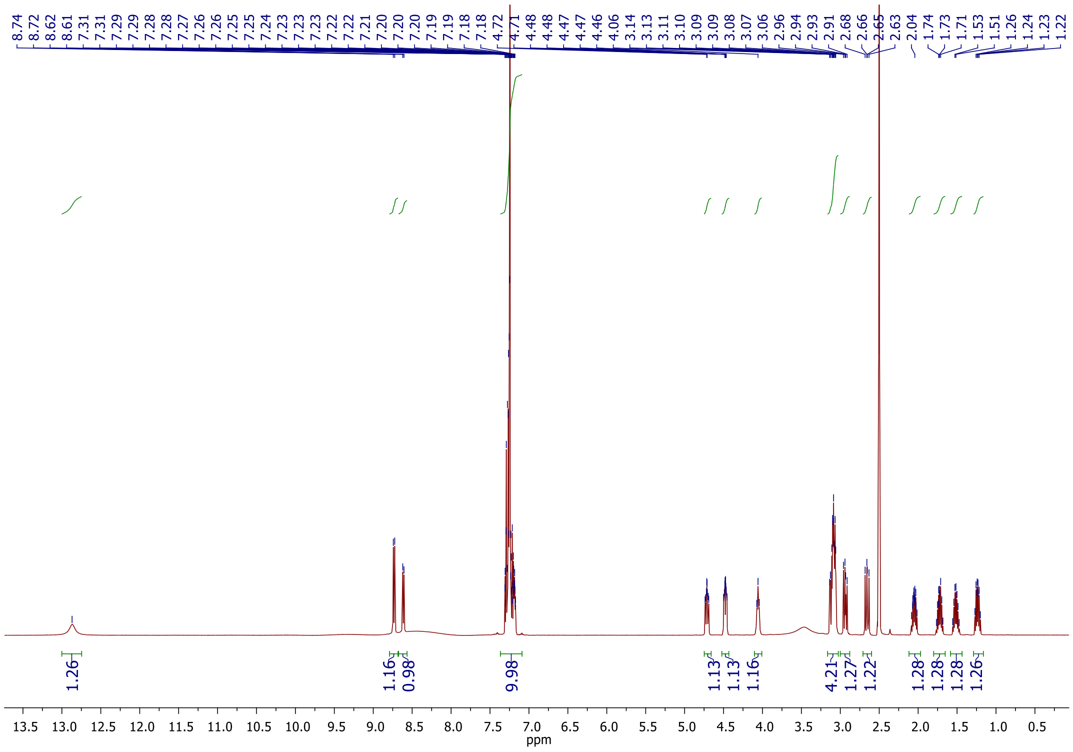

Fig. S14. ^{1}H-NMR spectrum of **2b**.

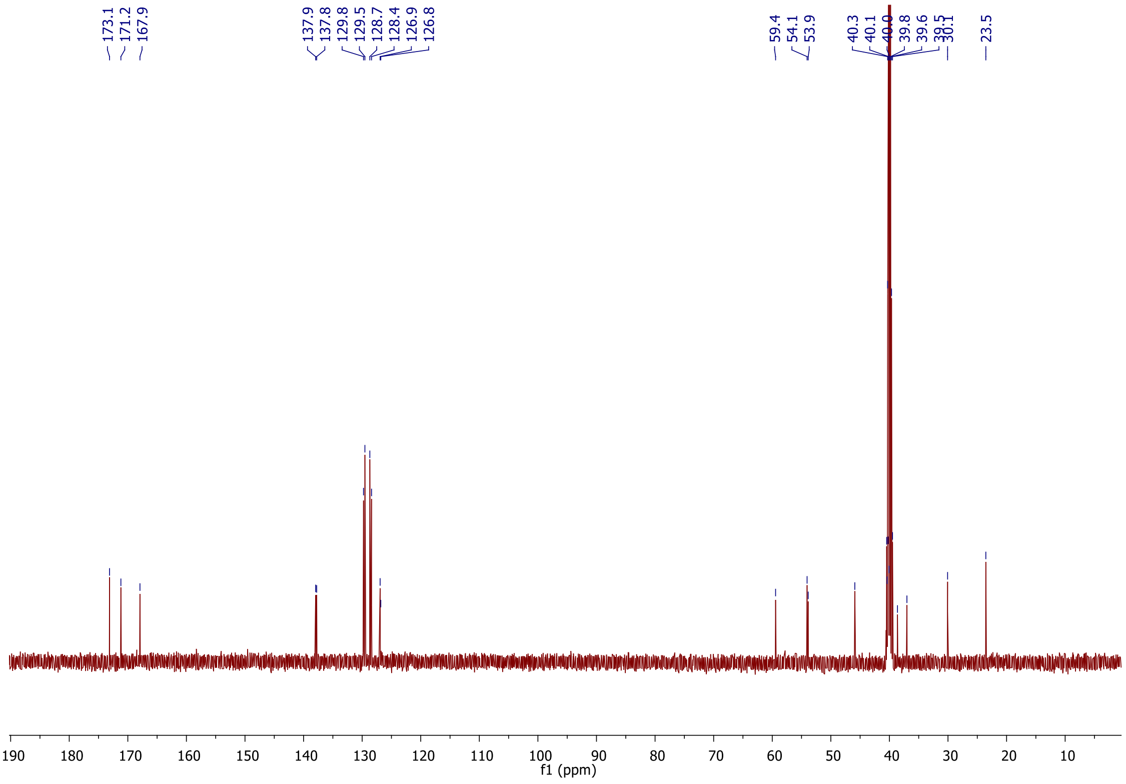

Fig. S15. ^{13}C-NMR spectrum of **2b**.

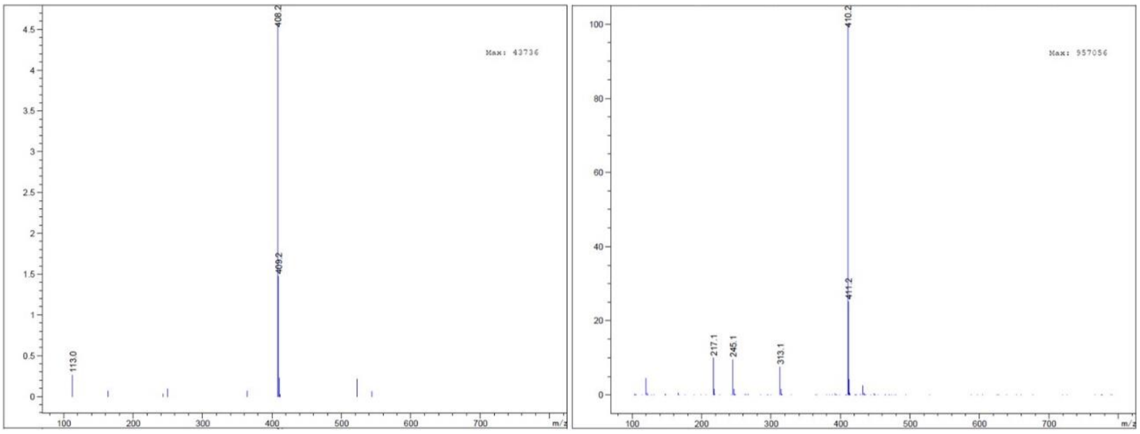

Fig. S16. ESI-MS spectra of **2b** in the positive (left) and negative (right) ion mode.

3a: L-Pro-D-Phe-L-Phe

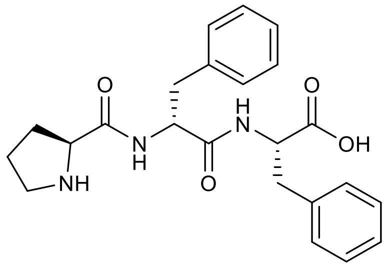

Fig. S17. Chemical structure of **3a**.

^{1}H NMR (500 MHz, DMSO-d_6) δ 8.66 (dd, J = 8.7 Hz, 2H, NH), 7.31-7.22 (m, 4H, Ar), 7.22-7.11 (m, 4H, Ar), 7.09-7.04 (m, 2H, Ar), 4.76-4.64 (m, 1H, αCH), 4.49 (ddd, J = 10.0, 8.6, 4.6 Hz, 1H, αCH), 4.04 (dd, J = 7.7 Hz, 1H, αCH), 3.12 (ddd, J = 11.5, 5.8 Hz, 1H, ProCH$_2$), 3.09 – 2.98 (m, 2H, ProCH$_2$, βCH$_2$), 2.92 – 2.77 (m, 1H, βCH$_2$), 2.71 (dd, J = 13.6, 3.7 Hz, 1H, βCH$_2$), 2.39 (dd, J = 13.5, 10.7 Hz, 1H, βCH$_2$), 2.13 – 1.93 (m, 1H, ProCH$_2$), 1.71 (m, J = 14.5, 7.3 Hz, 1H, ProCH$_2$), 1.61 – 1.44 (m, 1H, ProCH$_2$), 1.23 (m, J = 44.2, 22.1 Hz, 1H, ProCH$_2$). **^{13}C NMR** (125 MHz, DMSO-d_6) δ 173.4, 170.7, 168.2 (3 x CO); 138.0, 137.8, 129.8, 129.7, 128.6, 128.3, 126.9, 126.8 (Ar); 59.4, 54.0, 53.9 (3 x αC); 45.9 (C-Pro); 38.8, 37.6 (2 x βC); 30.0, 23.7 (2 x C-Pro). **MS (ESI)** m/z 410.1 $(M+H)^+$ $C_{23}H_{27}N_3O_4$ requires 409.2.

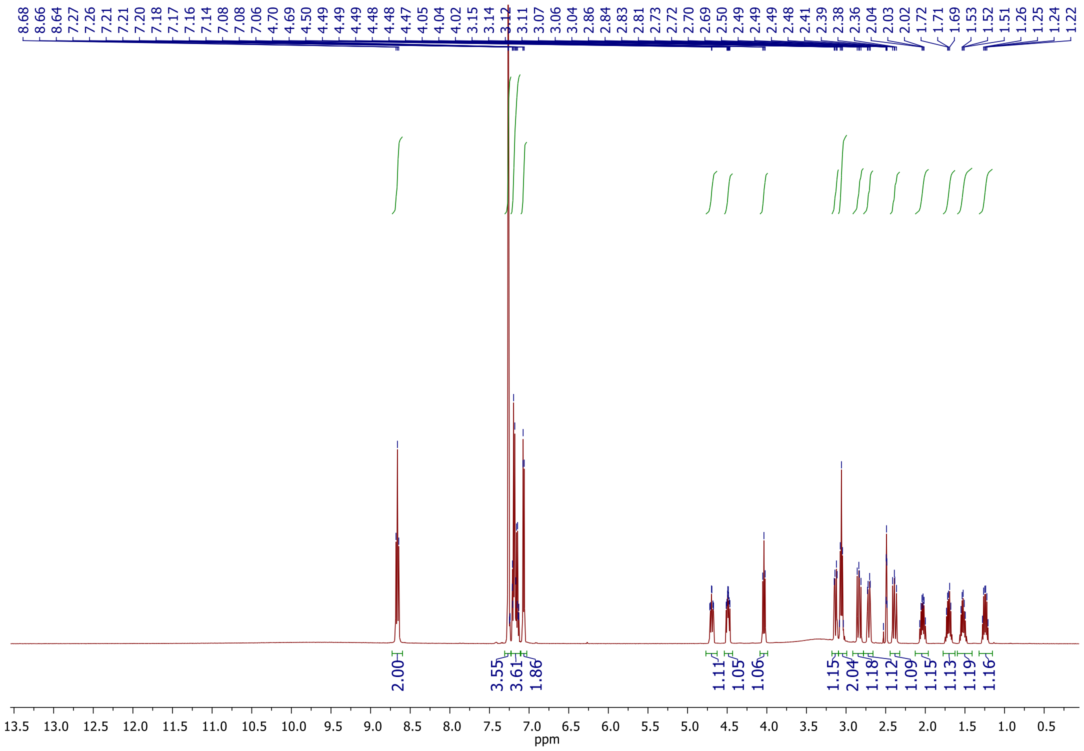

Fig. S18. ^{1}H-NMR spectrum of **3a**.

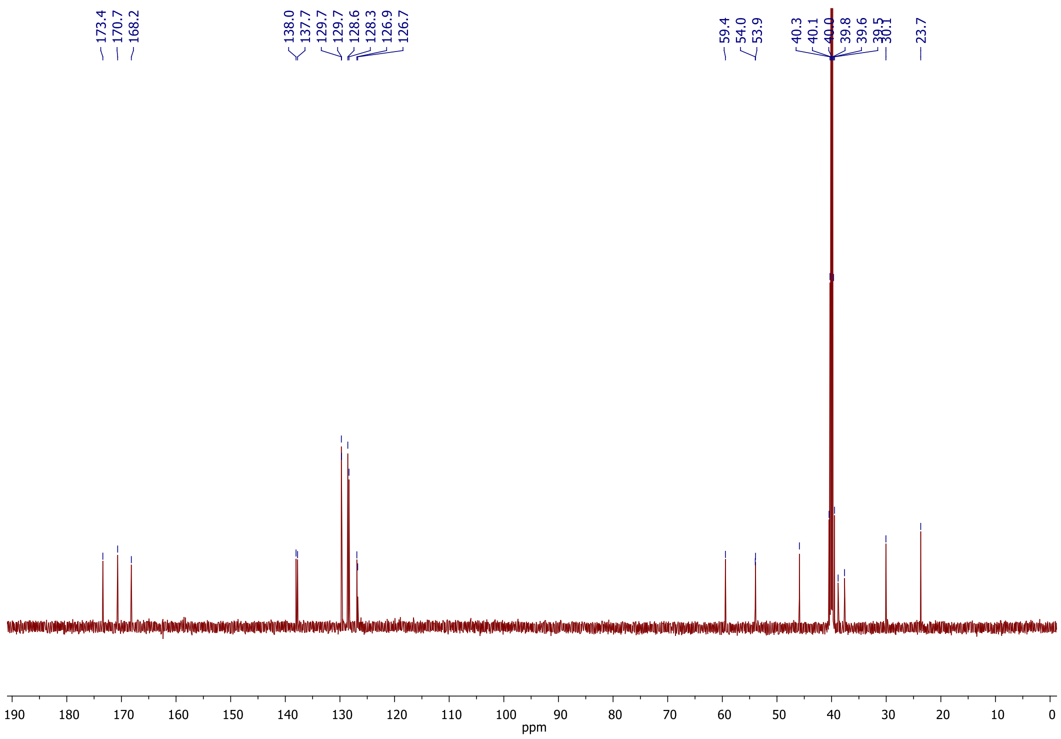

Fig. S19. ^{13}C-NMR spectrum of **3a**.

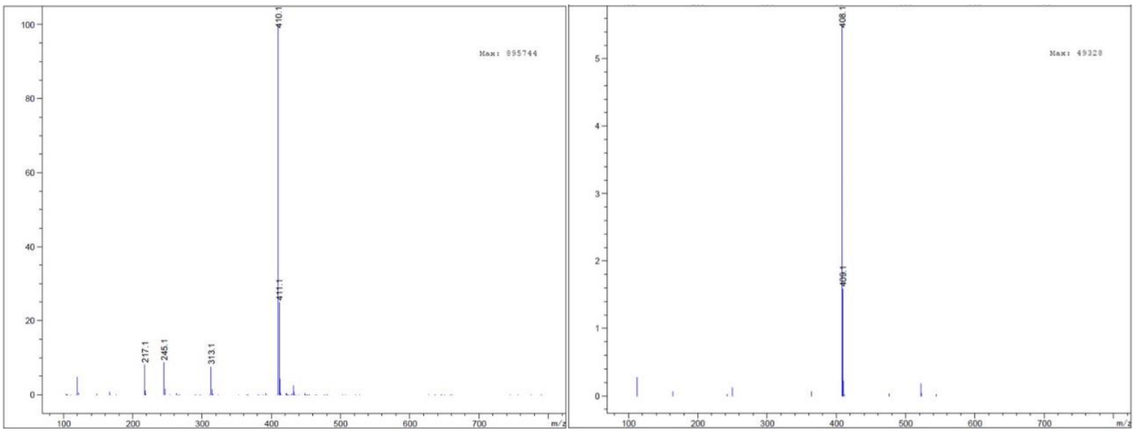

Fig. S20. ESI-MS spectra of **3a** in the positive (left) and negative (right) ion mode.

3b: D-Pro-L-Phe-D-Phe

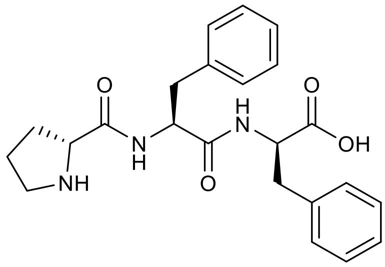

Fig. S21. Chemical structure of **3b**.

^{1}H NMR (500 MHz, DMSO-d_6) δ 8.70 d, J = 8.4 Hz, 1H, NH), 8.68 (d, J = 9.0 Hz, 1H, NH), 7.29 – 7.11 (m, 8H, Ar), 7.11 – 7.00 (m, 2H, Ar), 4.70 (ddd, J = 10.6, 9.4, 3.8 Hz, 1H, αCH), 4.50 (ddd, J = 10.2, 8.6, 4.6 Hz, 1H, αCH), 4.05 (dd, J = 7.7 Hz, 1H, αCH), 3.10 – 3.01 (m, 3H, 2 x ProCH$_2$, βCH$_2$), 2.84 (dd, J = 13.7, 10.2 Hz, 1H, βCH$_2$), 2.70 (dd, J = 13.6, 3.7 Hz, 1H, βCH$_2$), 2.38 (dd, J = 13.6, 10.8 Hz, 1H, βCH$_2$), 2.04 (ddd, J = 15.8, 14.2, 7.9 Hz, 1H, ProCH$_2$), 1.71 (m, J = 14.5, 7.4 Hz, 1H, ProCH$_2$), 1.59 – 1.47 (m, 1H, ProCH$_2$), 1.24 (m, J = 14.7, 7.3 Hz, 1H, ProCH$_2$). **^{13}C NMR** (125 MHz, DMSO-d_6) δ 173.3, 170.7, 168.0 (3 x CO); 137.9, 137.7, 129.7, 129.6, 128.6, 128.4, 127.0, 126.8 (Ar); 59.5, 54.0, 53.8 (3 x αC); 45.9 (C-Pro); 38.8, 37.6 (2 x βC); 30.0, 23.6 (2 x C-Pro). **MS (ESI)** m/z 410.2 $(M+H)^+$ $C_{23}H_{27}N_3O_4$ requires 409.2.

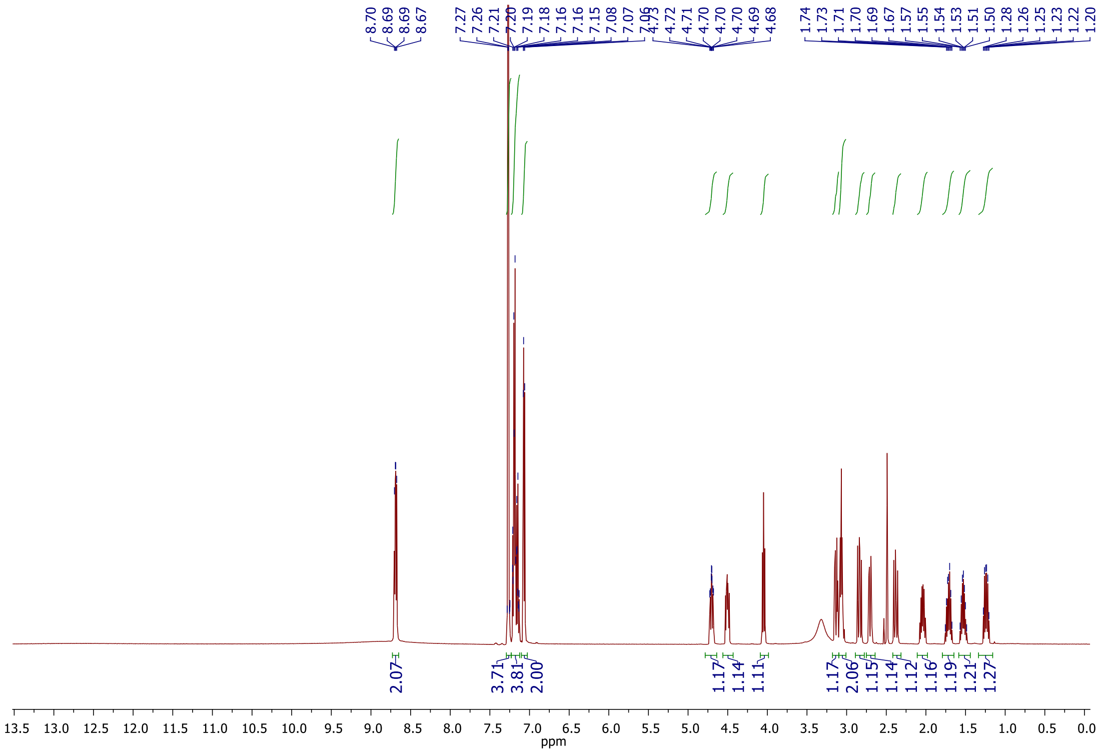

Fig. S22. ^{1}H-NMR spectrum of **3b**.

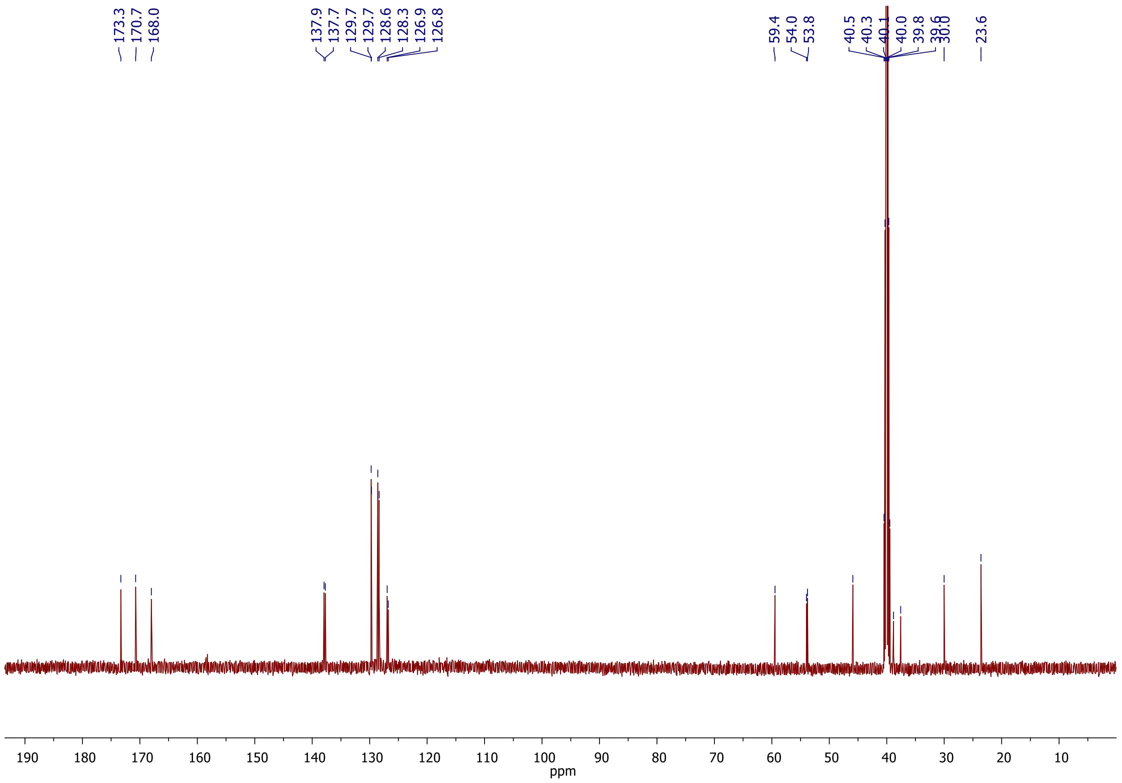

Fig. S23. ^{13}C-NMR spectrum of **3b**.

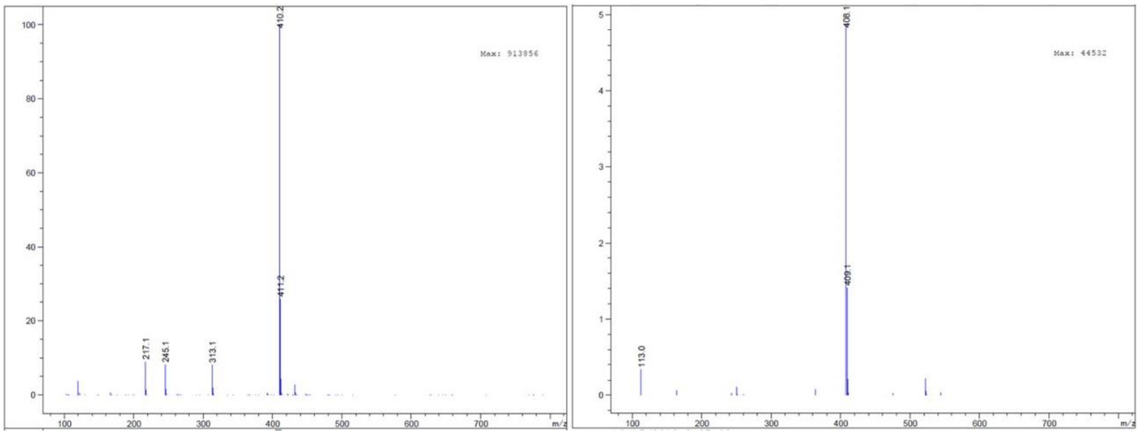

Fig. S24. ESI-MS spectra of **3b** in positive (left) and negative (right) ion mode.

4a: L-Pro-L-Phe-D-Phe

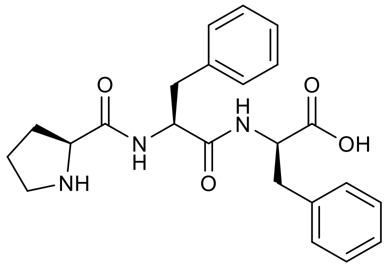

Fig. S25. Chemical structure of **4a**.

^{1}H NMR (500 MHz, DMSO-d_6) δ 12.88 (s, 1H, OH), 8.64 (d, J = 8.6 Hz, 1H, NH), 8.57 (d, J = 8.4 Hz, 1H, NH), 7.32 – 7.12 (m, 8 H, Ar), 7.11 – 7.07 (m, 2H, Ar), 4.59 (ddd, J = 9.7, 4.1 Hz, 1H, αCH), 4.48 (ddd, J = 10.0, 8.5, 4.6 Hz, 1H, αCH), 4.04 (dd, J = 8.4, 6.8 Hz, 1H, αCH), 3.21 – 3.04 (m, 3H, 2 x ProCH$_2$, βCH$_2$), 2.83 (dd, J = 13.8, 10.1 Hz, 1H, βCH$_2$), 2.72 (dd, J = 13.8, 4.0 Hz, 1H, βCH$_2$), 2.53 – 2.44 (m, 1H, βCH$_2$), 2.31 – 2.18 (m, 1H, ProCH$_2$), 1.92 – 1.67 (m, 3H, 3 x ProCH$_2$). **^{13}C NMR** (125 MHz, DMSO-d_6) δ 173.2, 170.7, 168.3 (3 x CO); 137.9, 137.8), 129.7, 129.6, 128.6, 128.5, 127.0 (Ar); 59.2, 54.6, 53.8 (3 x αC); 46.1 (C-Pro); 38.1, 37.5 (2 x βC); 30.0, 23.9 (2 x C-Pro). **MS (ESI)** m/z 410.2 $(M+H)^+$ $C_{23}H_{27}N_3O_4$ requires 409.2.

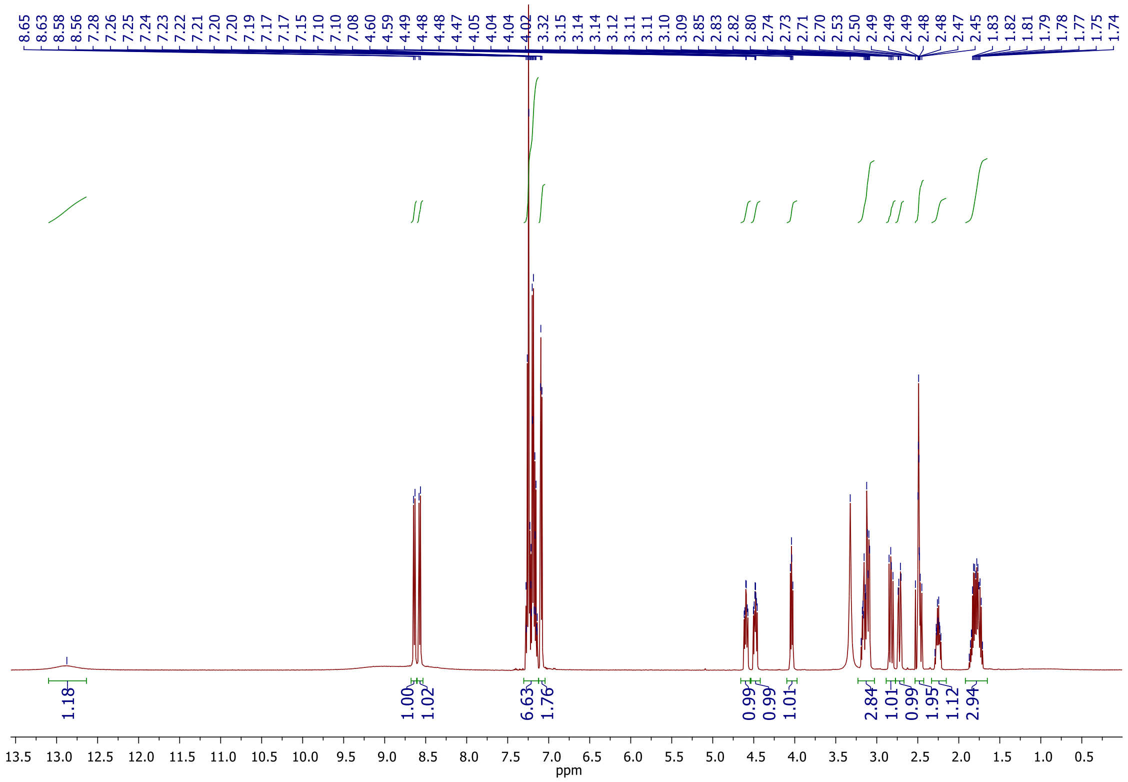

Fig. S26. ^{1}H-NMR spectrum of **4a**.

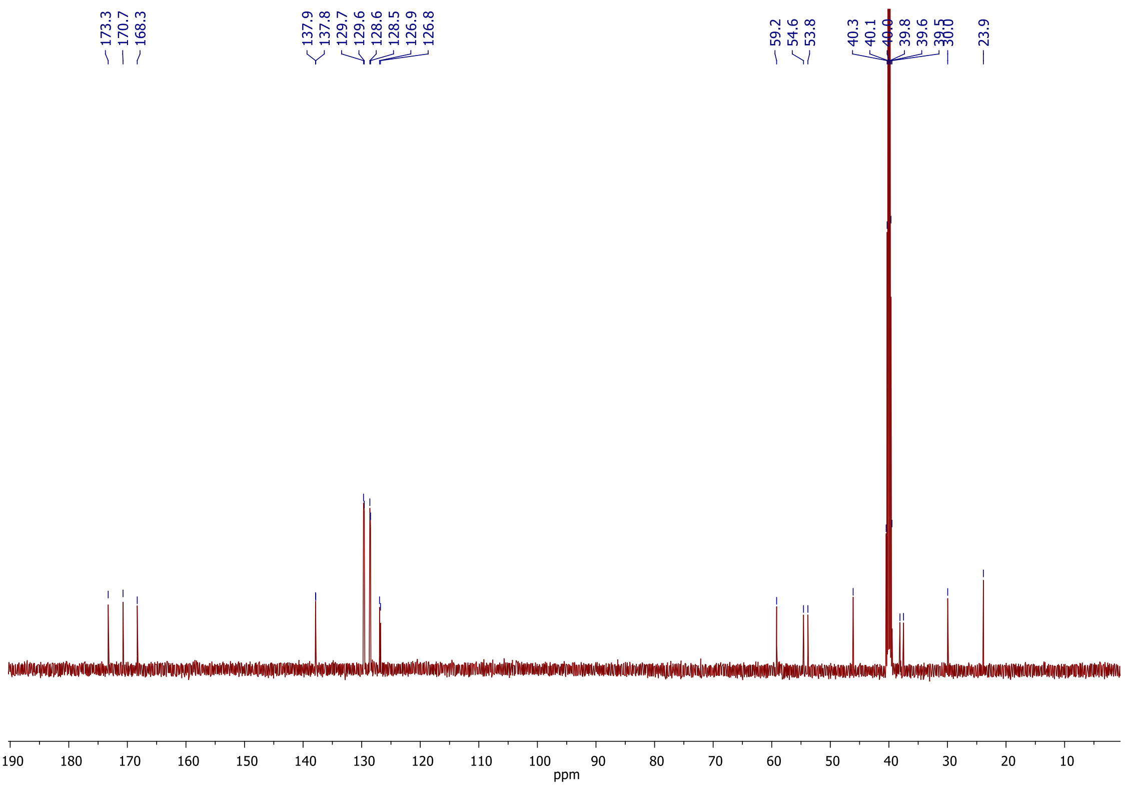

Fig. S27. ^{13}C-NMR spectrum of **4a**.

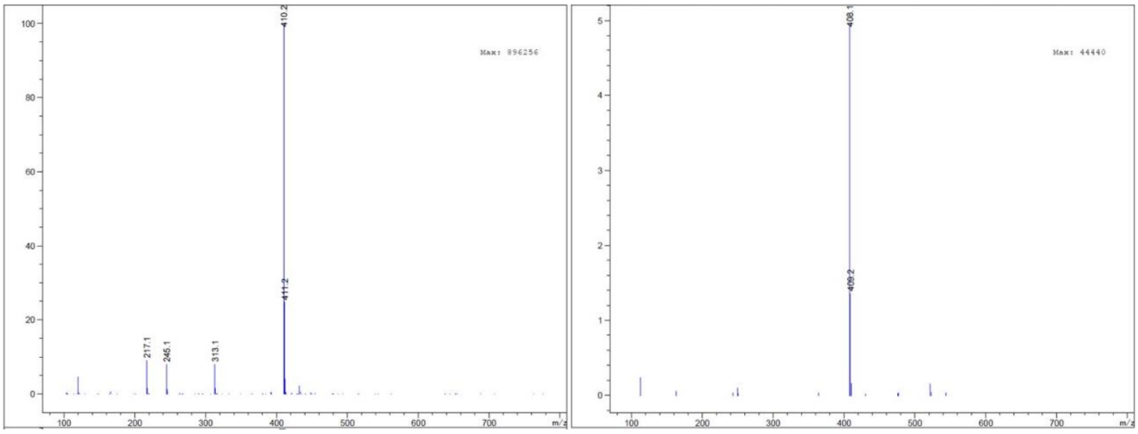

Fig. S28. ESI-MS spectra of **4a** in the positive (left) and negative (right) ion mode.

4b: D-Pro-D-Phe-L-Phe

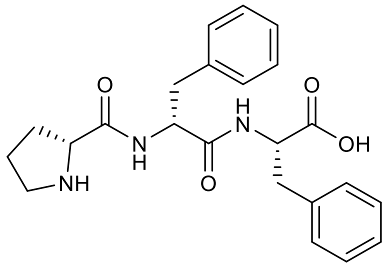

Fig. S29. Chemical structure of **4b**.

^{1}H NMR (500 MHz, DMSO-d_6) δ 12.92 (s, 1H, OH), 8.65 (d, J = 8.6 Hz, 1H, NH), 8.57 (d, J = 8.4 Hz, 1H, NH), 7.31 – 7.13 (m, 8H, Ar), 7.13 – 7.06 (m, 2H, Ar), 4.59 (ddd, J = 9.6, 4.1 Hz, 1H, αCH), 4.48 (ddd, J = 10.0, 8.5, 4.6 Hz, 1H, αCH), 4.05 (dd, J = 8.2, 6.9 Hz, 1H, αCH), 3.21 – 3.04 (m, 3H, 2 x ProCH$_2$, βCH$_2$), 2.83 (dd, J = 13.7, 10.1 Hz, 1H, βCH$_2$), 2.72 (dd, J = 13.8, 4.0 Hz, 1H, βCH$_2$), 2.49 (m, J = 13.8, 11.6 Hz, 1H, βCH$_2$), 2.31 – 2.19 (m, 1H, ProCH$_2$), 1.98 – 1.65 (m, 3H, ProCH$_2$). **^{13}C NMR** (125 MHz, DMSO-d_6) δ 173.3, 170.8, 168.3 (3 x CO); 137.9, 137.8, 129.7, 129.6, 128.6, 128.5, 126.9, 126.8 (Ar); 59.2, 54.6, 53.8 (3 x αC); 46.1 (C-Pro); 38.1, 37.5 (2 x βC); 30.0, 23.9 (2 x C-Pro). **MS (ESI)** m/z 410.1 $(M+H)^+$ $C_{23}H_{27}N_3O_4$ requires 409.2.

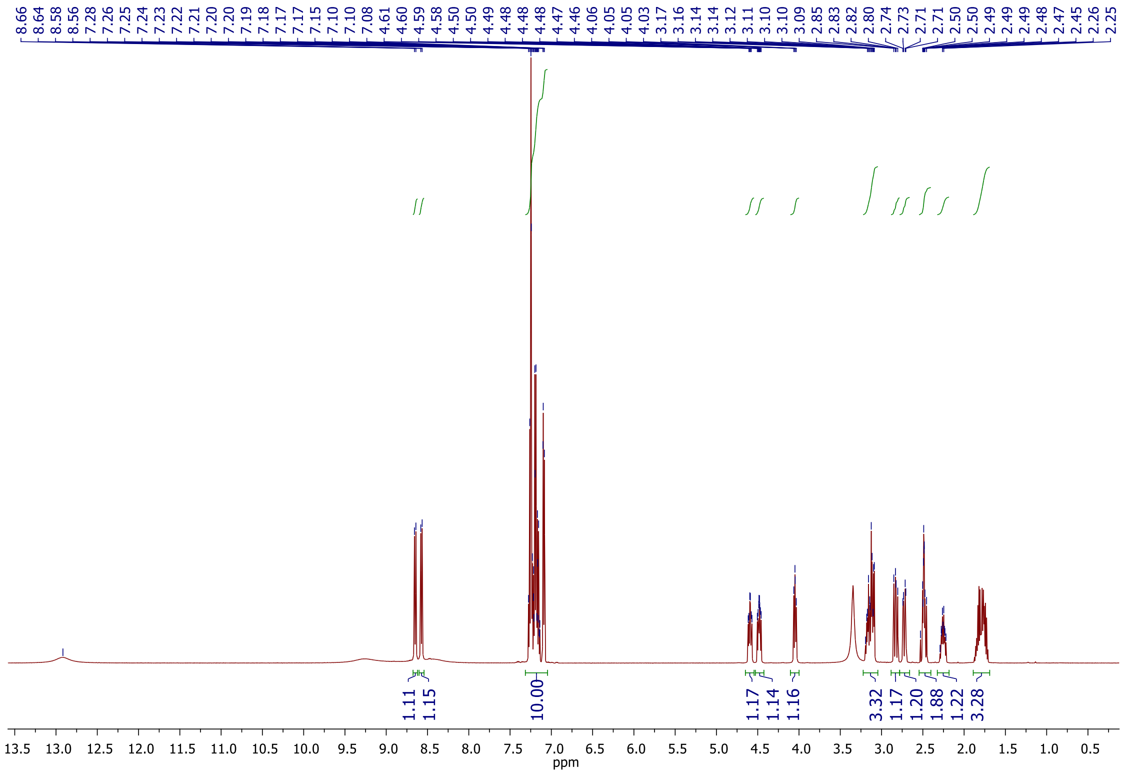

Fig. S30. ^{1}H-NMR spectrum of **4b**.

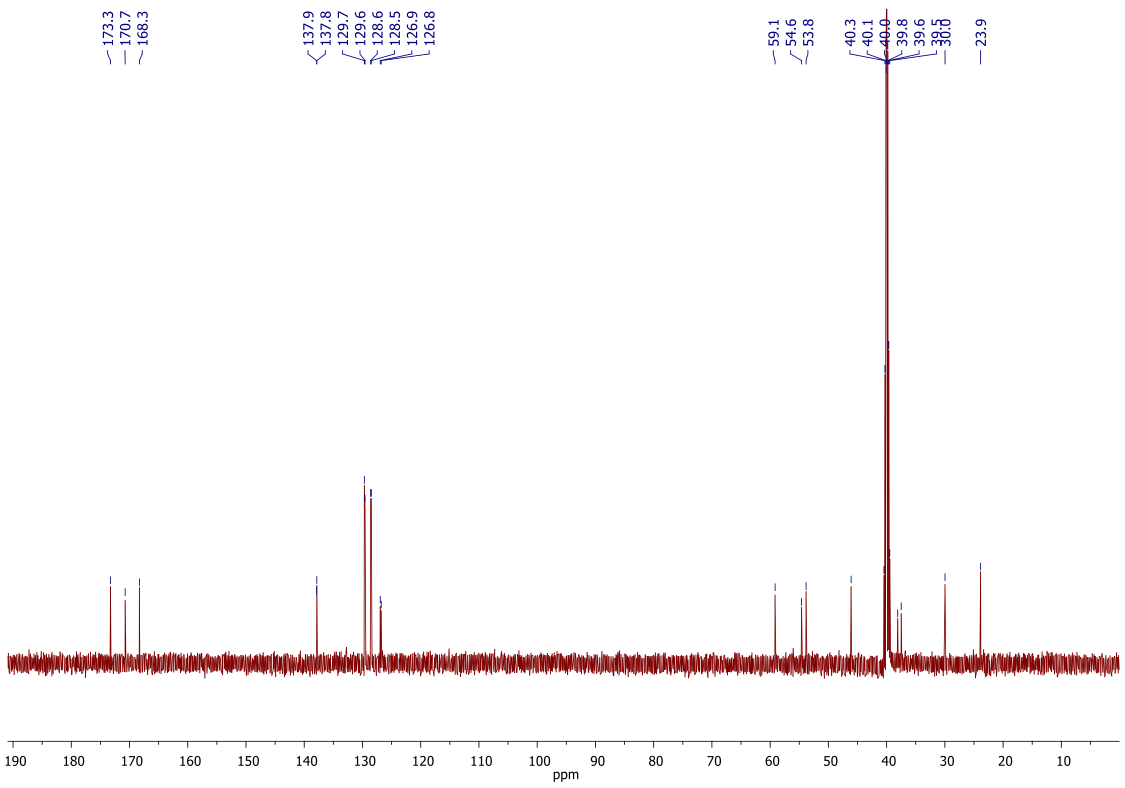

Fig. S31. ^{13}C-NMR spectrum of **4b**.

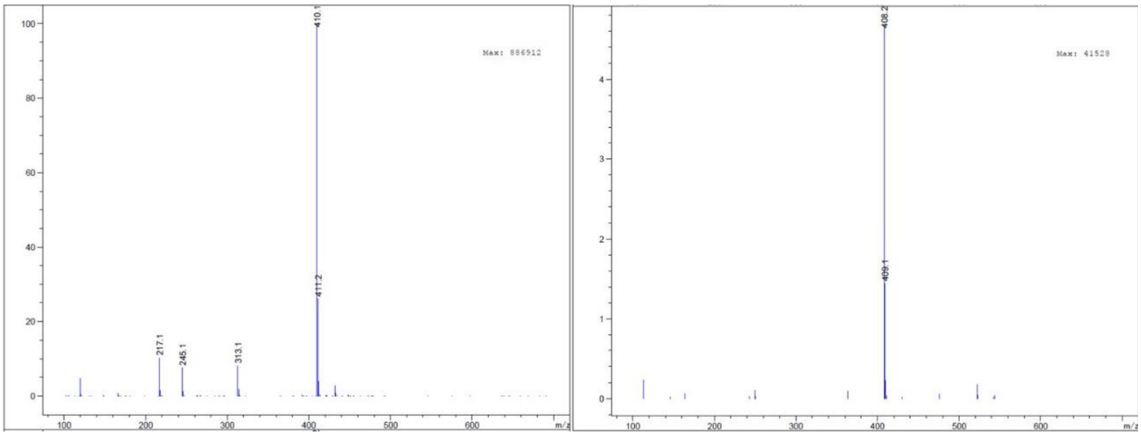

Fig S32. ESI-MS spectra of **4b** in the positive (left) and negative (right) ion mode.

S2. Analytical LC data for full series of Pro-Phe-Phe tripeptides

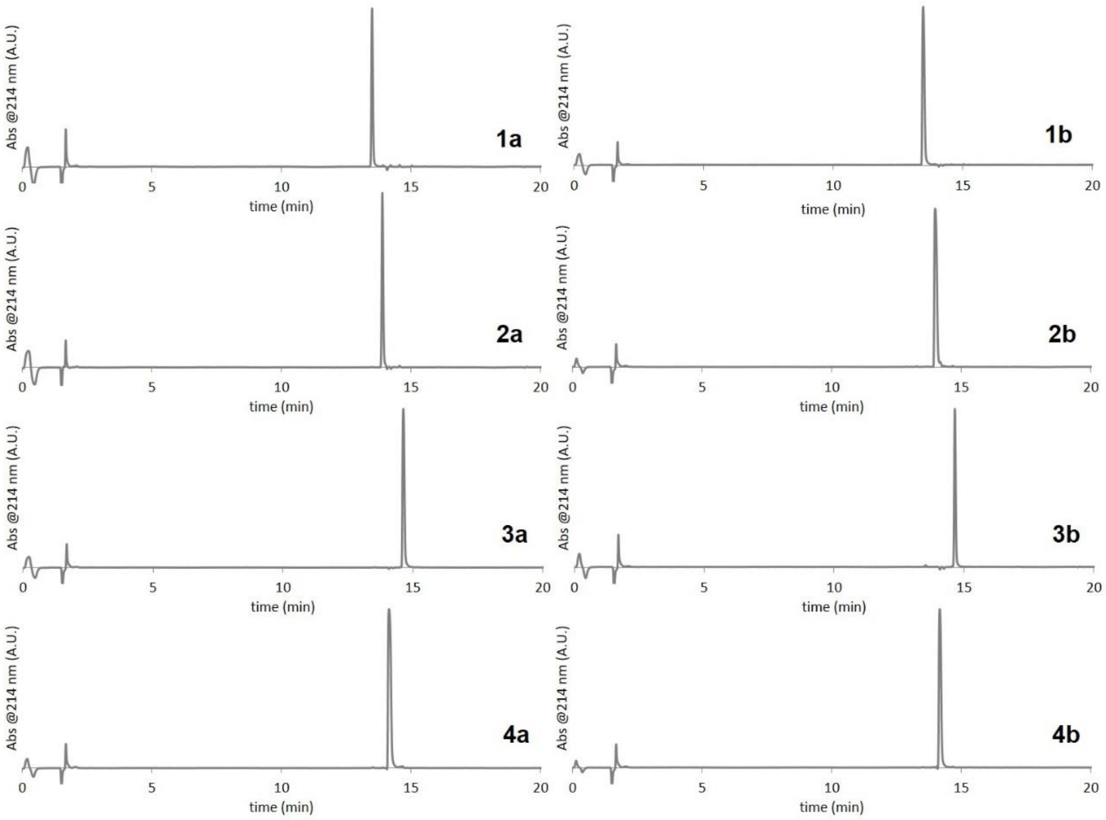

Fig S33. Analytical LC traces for each pair of enantiomers with Pro-Phe-Phe sequence. Retention times (Rt): 13.5 min (**1a/b**); 13.9 min (**2a/b**); 14.6 min. (**3a/b**); 14.2 min (**4a/b**).

S3. Rheological data for the hydrogel 2a and 2b.

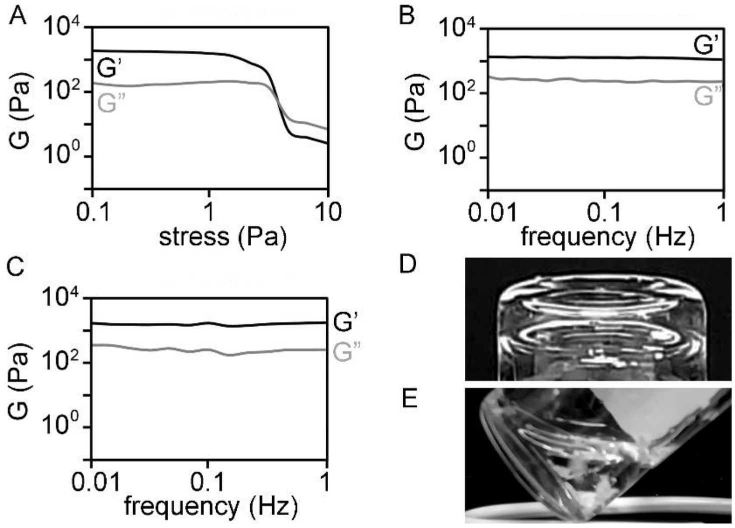

Fig. S34. A) Stress sweep for **2a** (24 mM). **B)** Frequency sweep for **2a** (24 mM). **C)** Frequency sweep for **2b** (24 mM). **D)** Photograph of **2b** hydrogel (24 mM). **E**) Photograph of **2b** insoluble aggregates after heating the hydrogel to 85 °C and cooling it down to RT. By contrast, if the hydrogel was heated to 40 °C, no aggregates were formed and the hydrogel disassembled into a clear solution reversibly, as indicated in the MS.

S4. TEM micrographs.

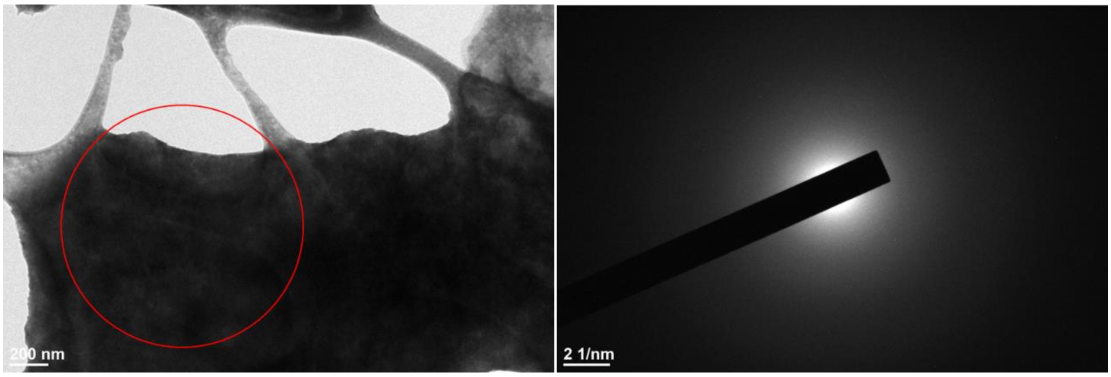

Fig. S35. TEM micrograph of **1a** amorphous aggregate (left) and selected area (red circle) electron diffraction (SAED, right) revealed it was amorphous.

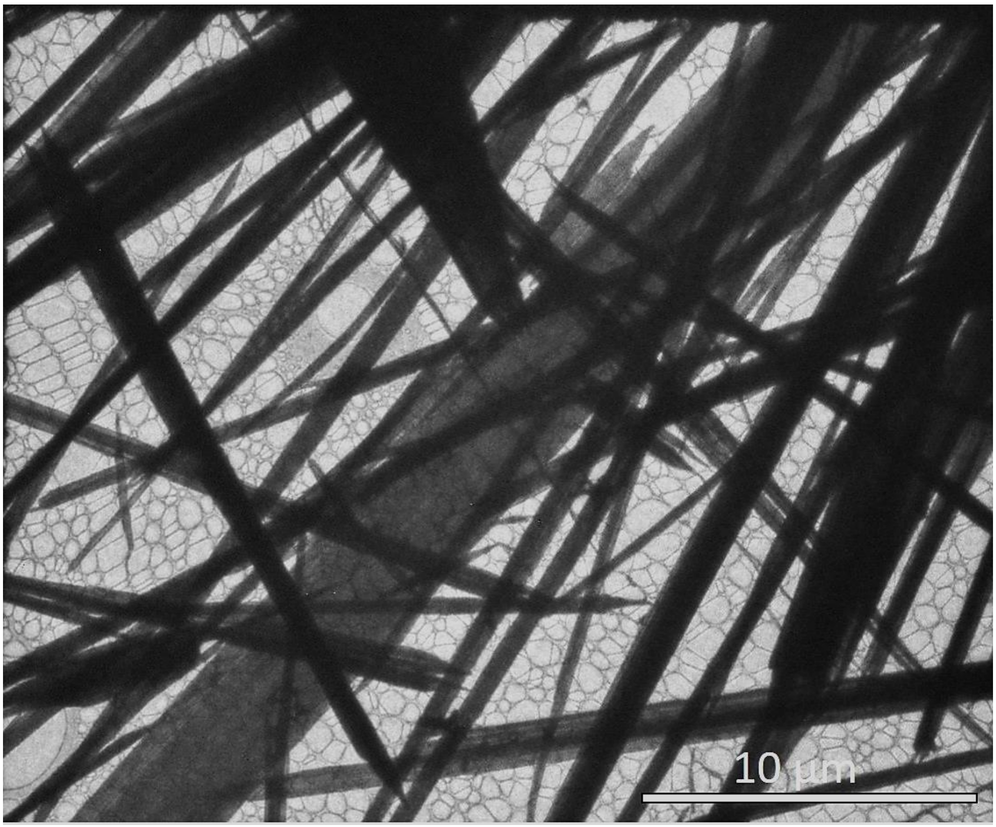

Fig. S36. TEM micrograph of **1a** microcrystals.

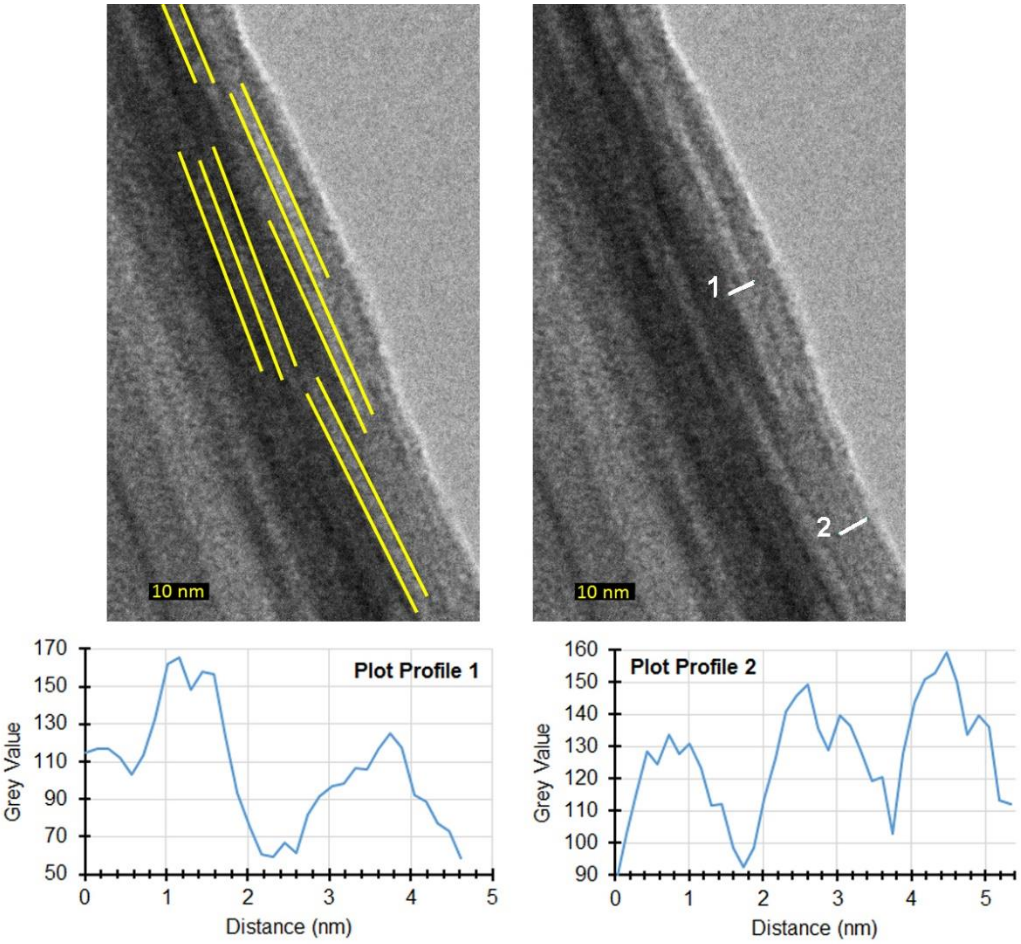

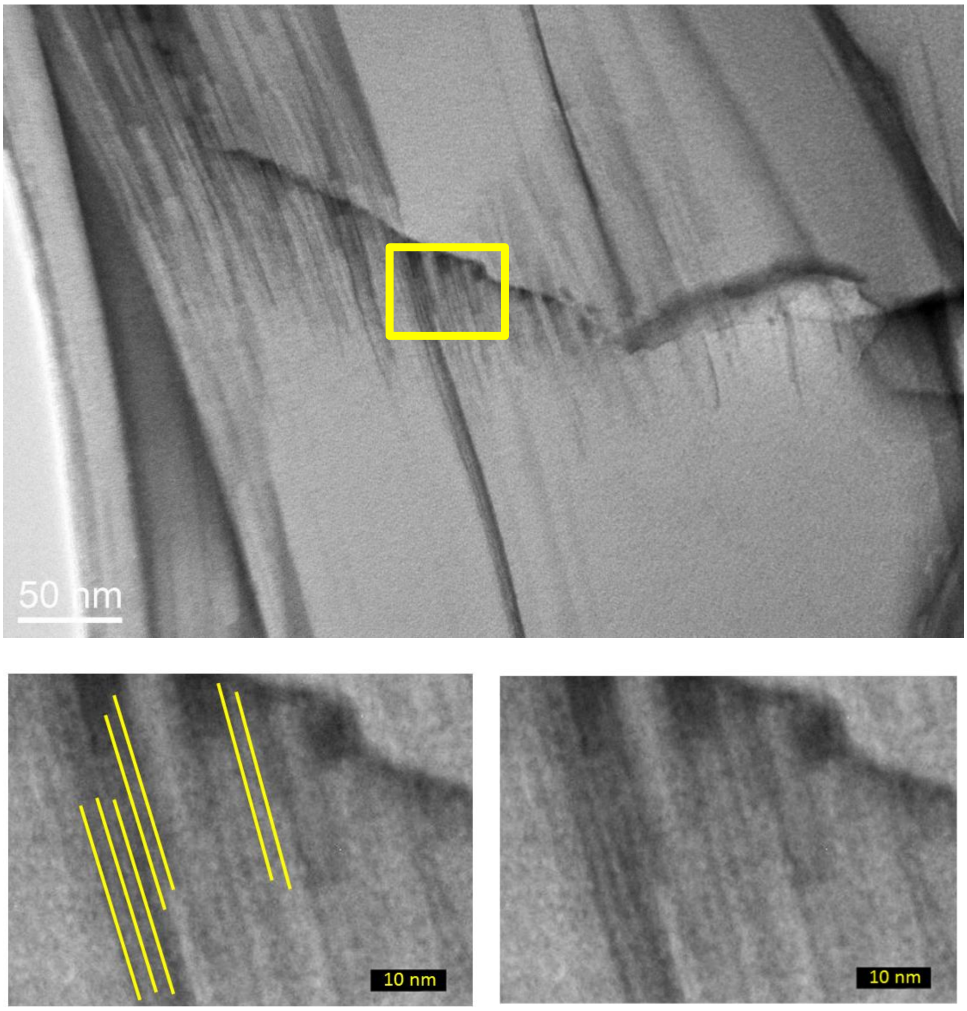

Fig. S37. TEM micrographs of **2a** fibrils forming nanotapes (detail) with plot profiles compatible with the presence of 1.6 ±0.2 nm-wide individual fibrils. The area in the yellow box is enlarged below: with (left) or without (right) yellow lines to guide the eye.

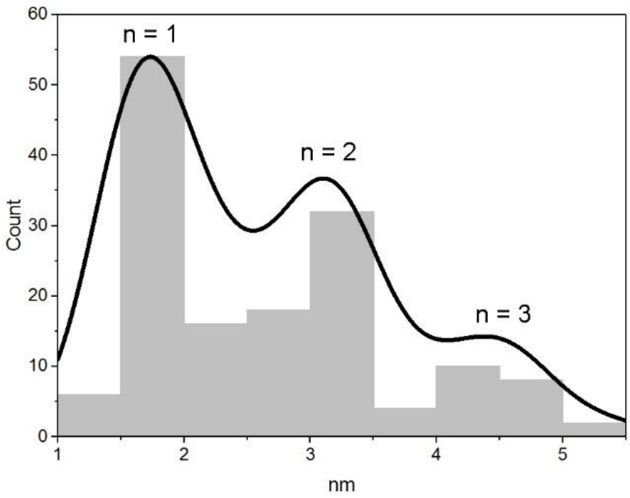

Chart S1. Fibril size distribution for **2a** (*counts* = 150) with mean diameter of individual (n=1) fibril corresponding to 1.6 ± 0.2 nm, two fibrils (n=2) corresponding to 3.2 ± 0.3 nm, and three fibrils (n=3) corresponding to 4.6 ± 0.4 nm.

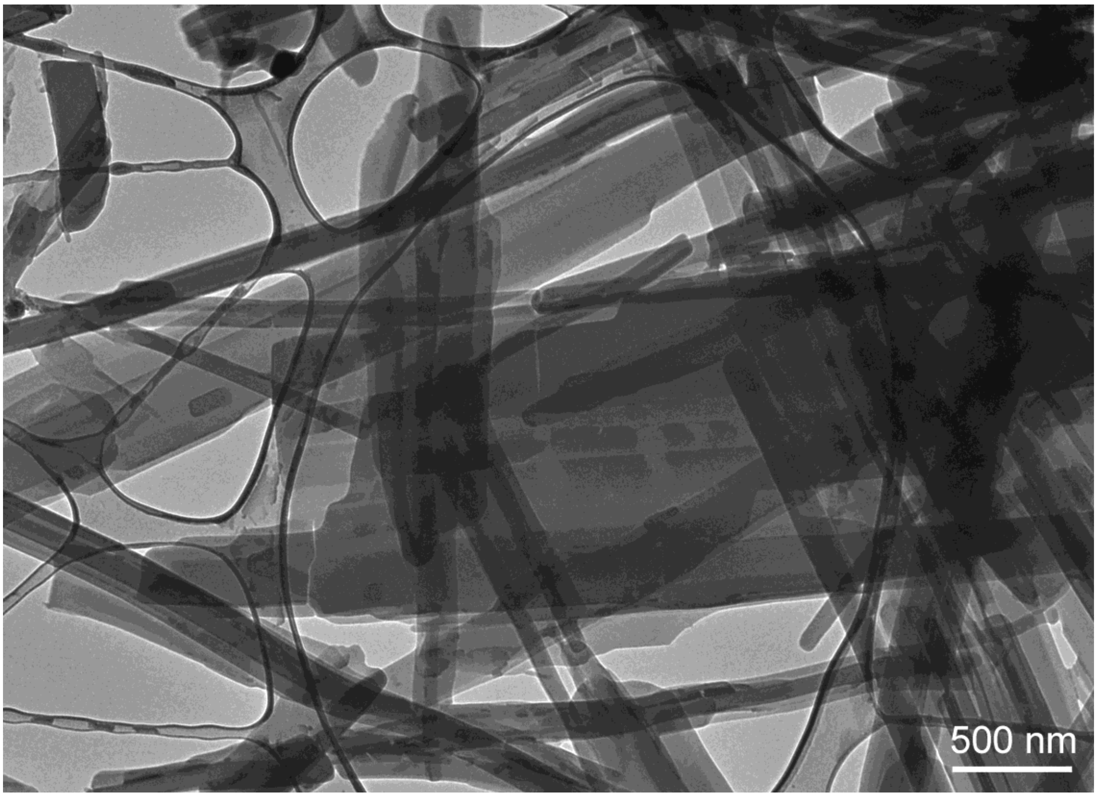

Fig. S38. TEM micrograph of **2a** nanotapes forming the gel.

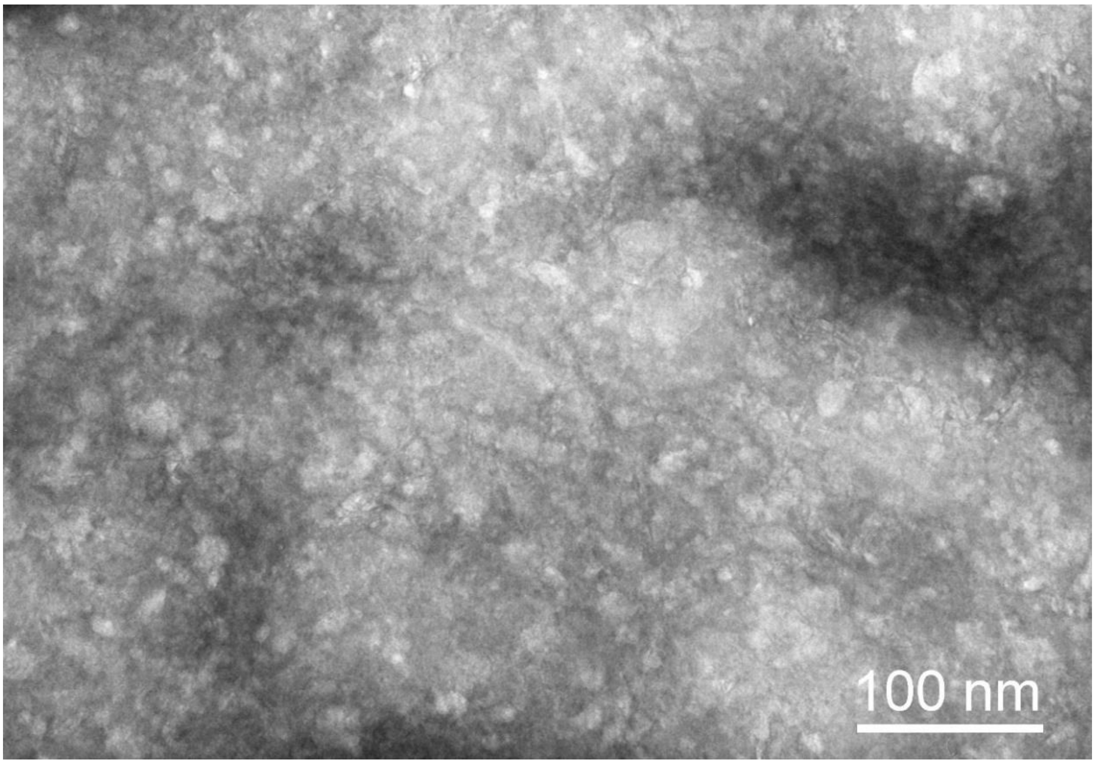

Fig. S39. TEM micrograph of **3a** nanoparticles.

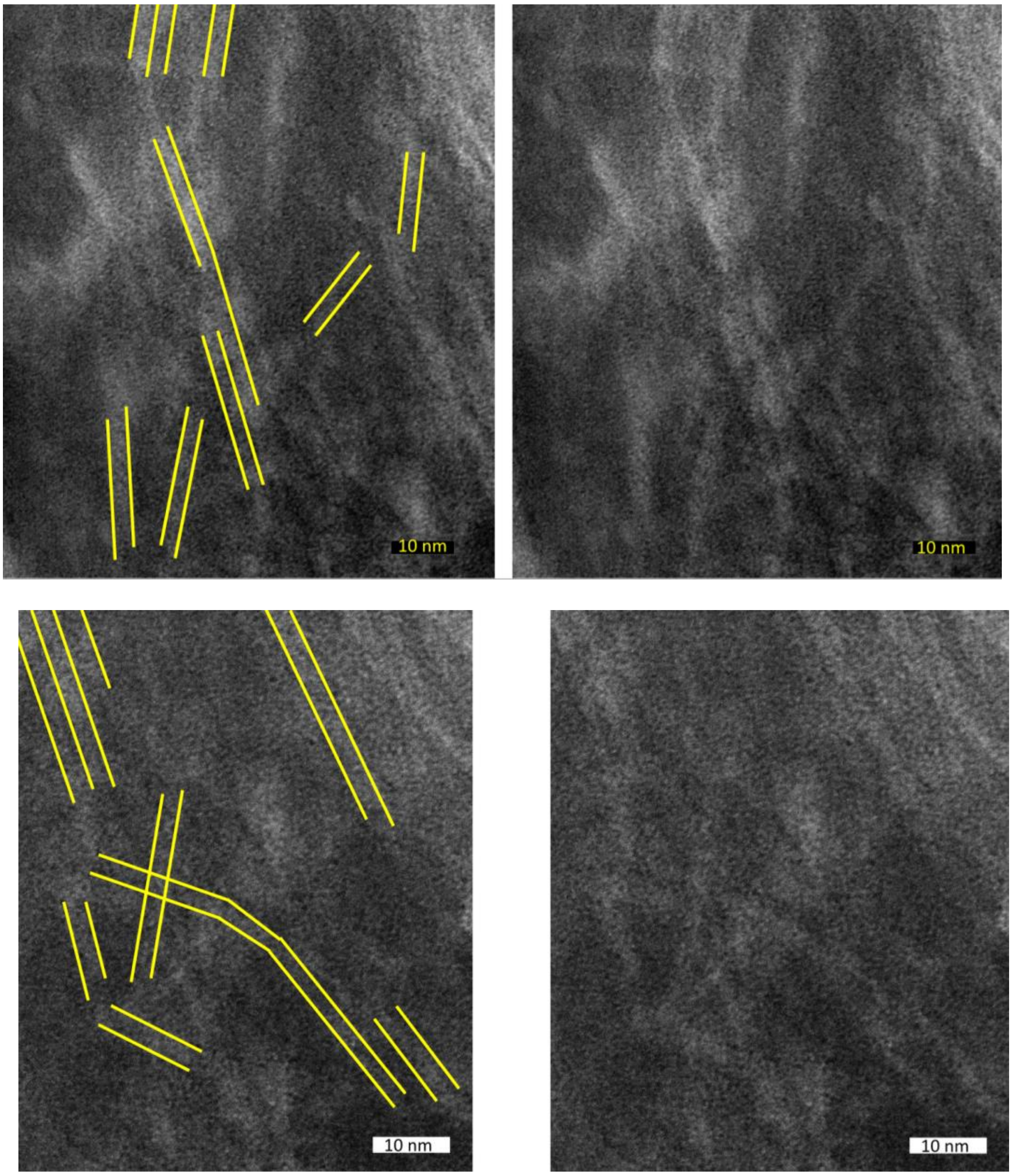

Fig. S40. TEM micrograph detail of **3a** rare instances of short, thin fibrils, with (left) or without (right) yellow lines to guide the eye.

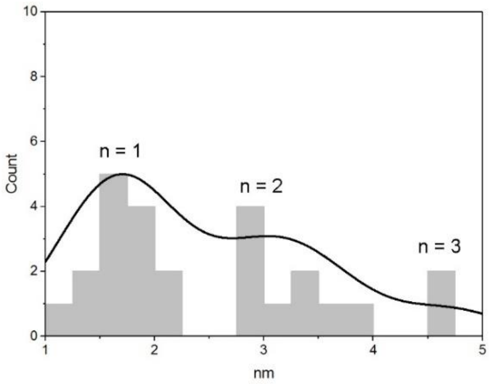

Chart S2. Fibril size distribution for **3a** (*counts* = 25, due to rare instances).

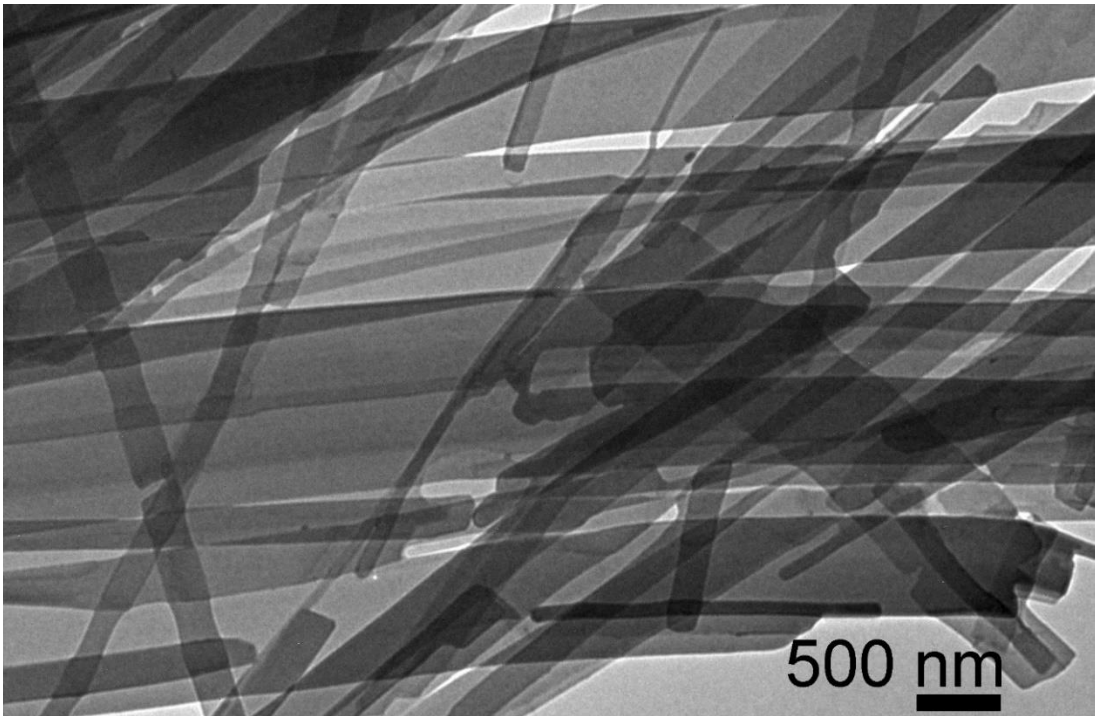

Fig. S41. TEM micrograph of **4a** nanotapes.

S5. AFM images

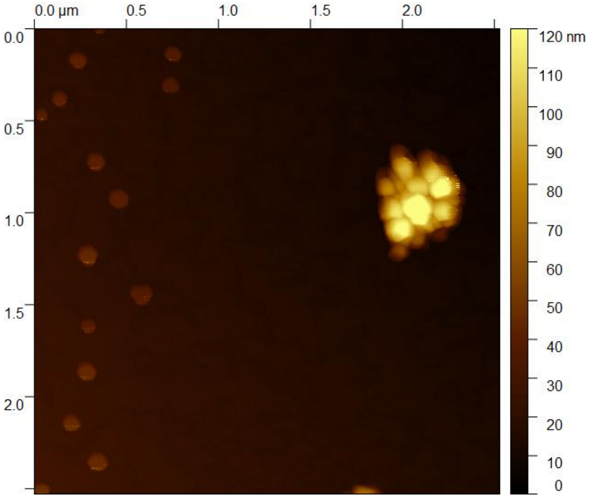

Fig. S42. AFM image of **1a** aggregates.

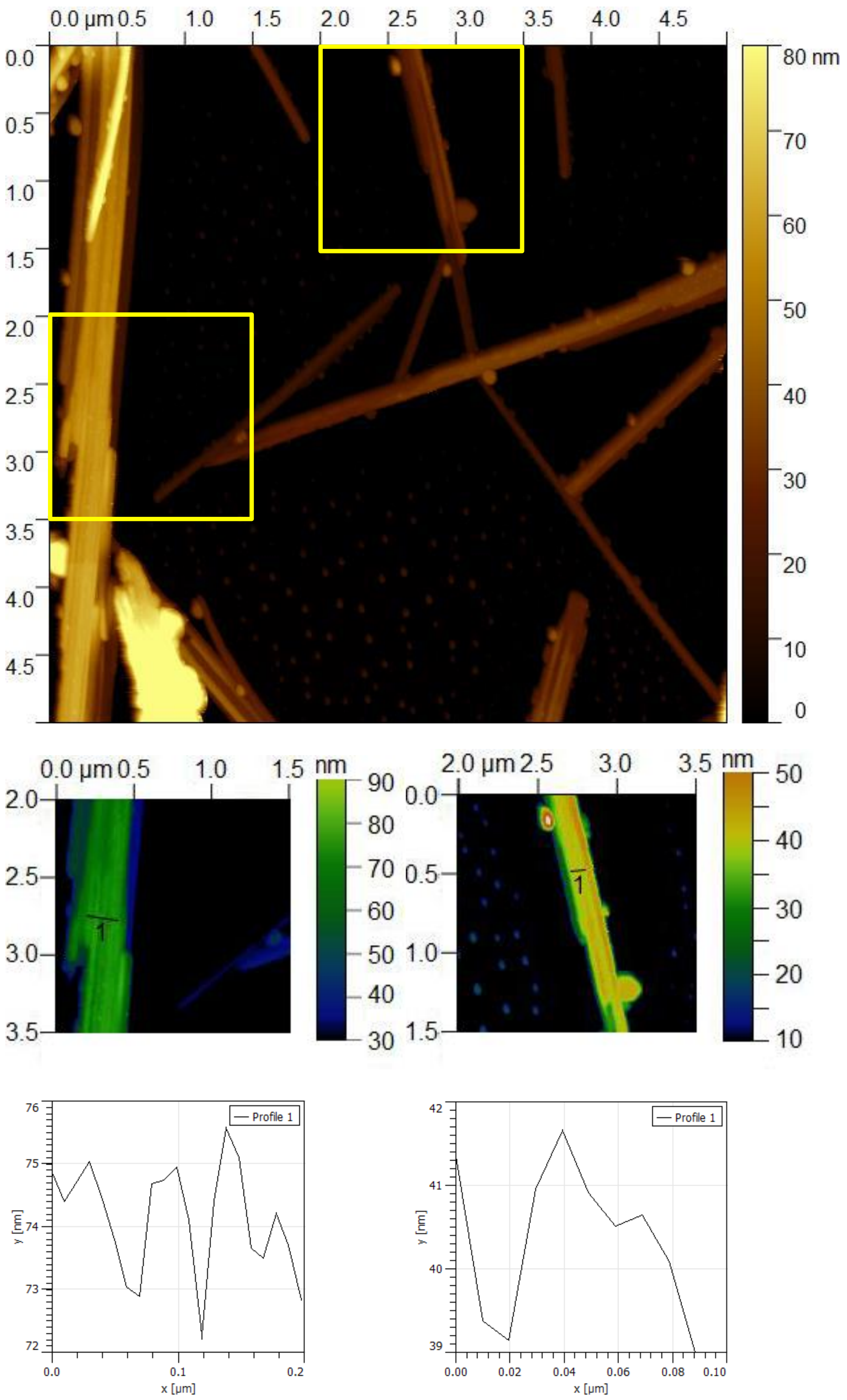

Fig. S43a. AFM image of **2a** nanotapes showing they arise from the lateral association of elongated structures with 1-2 nm step heights.

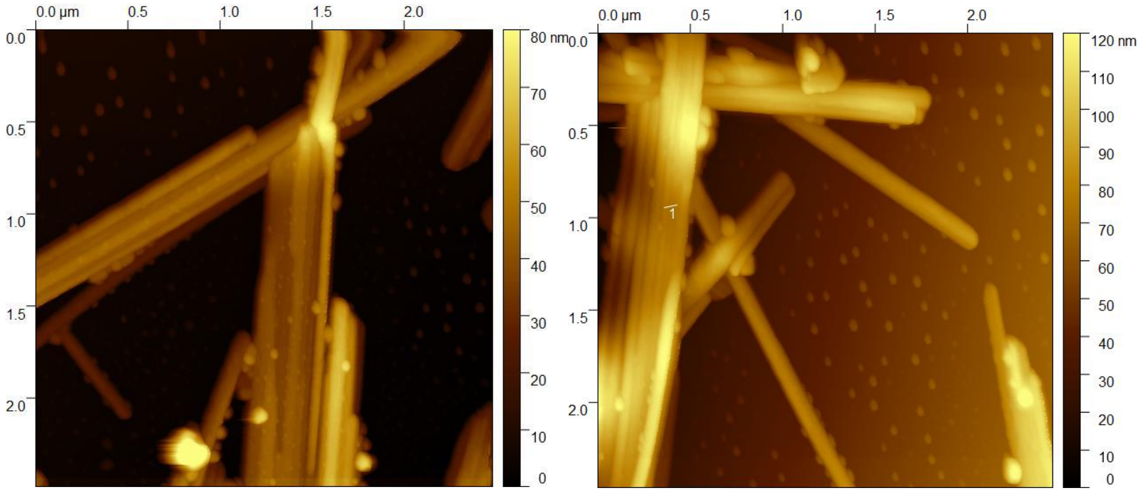

Fig. S43b. Additional AFM images of **2a** nanotapes.

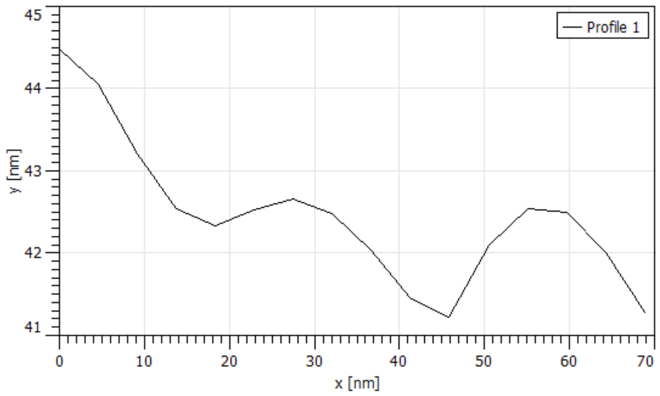

Chart S3. Height profile shown in Fig. S43b (right) confirmed 1-2 nm step heights.

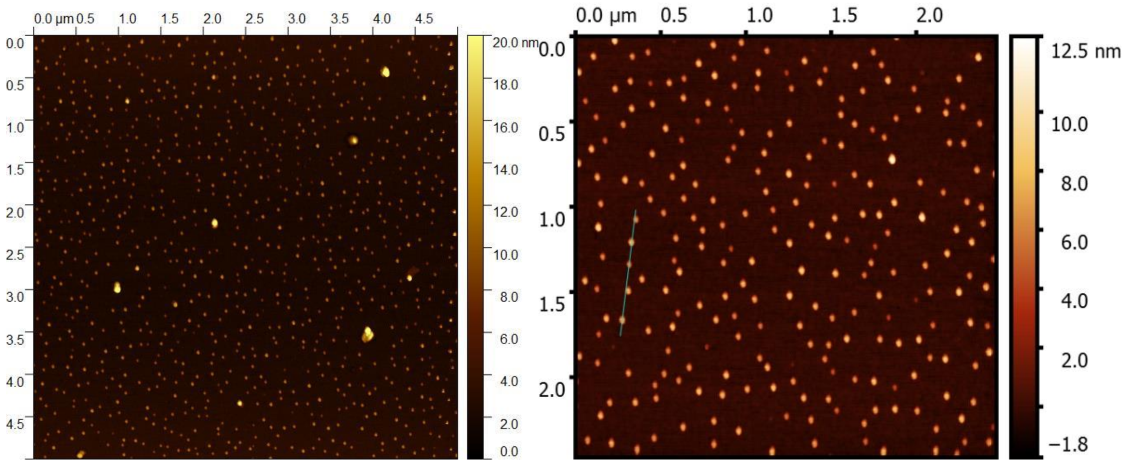

Fig. S44. AFM images of **3a** nanoparticles.

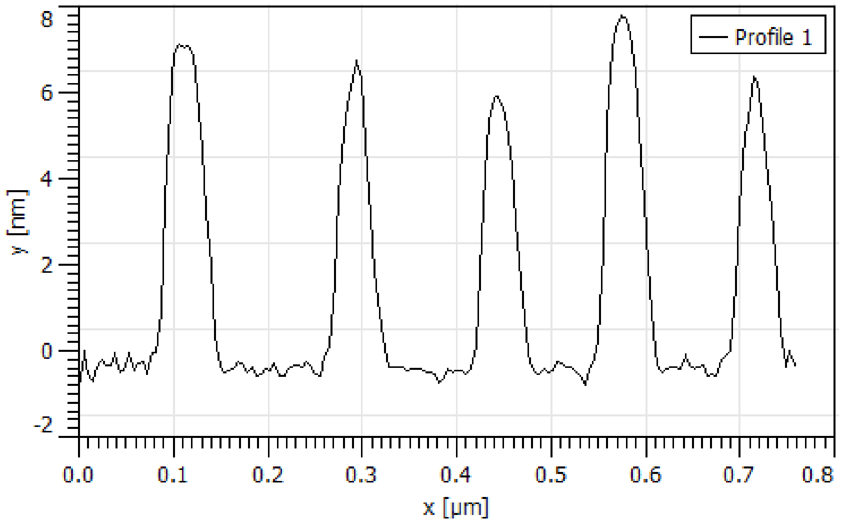

Chart S4. Height profile shown in Fig. S44 (right) showed homogeneous nanoparticle size (confirmed to be 8 ± 3 nm on 100 measures).

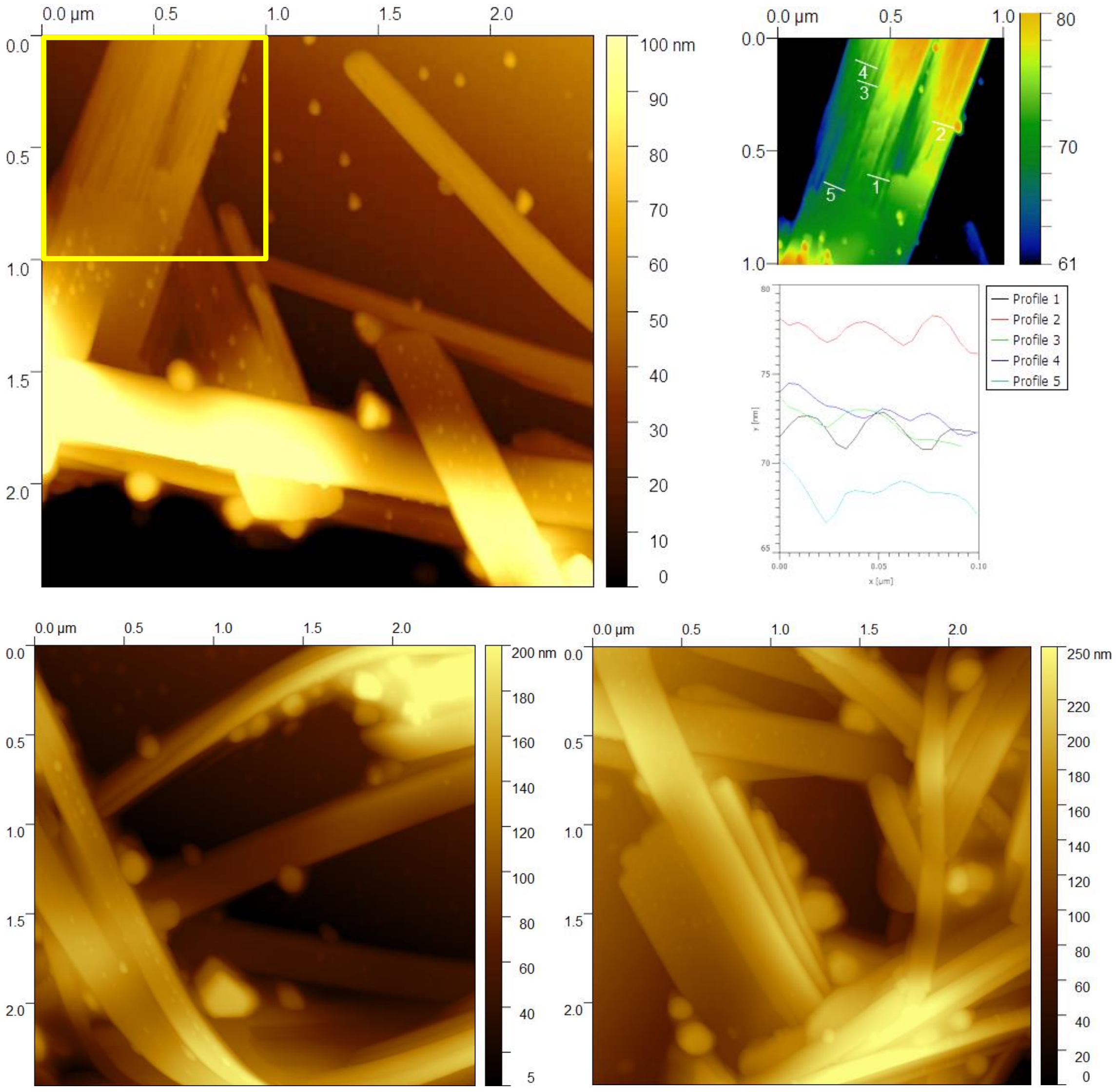

Fig. S45. AFM images of **4a** nanotapes, with height profiles showing 1-2 nm steps.

S6. Full-atom MD simulations of tripeptides.

Generation of tripeptide and Aβ structural models. Models of zwitterionic tripeptides were built upon the experimental structure (**2a**) or generated using the AmberTools19 package[1] and the VMD1.9.3 software[2] through in-house *tcl* scripts (**3a**). The initial coordinates of the protein were taken from the NMR structure with PDB ID 2NAO,[3] namely from the chain A of the corresponding *pdb* file.

MD simulations. Multi-copy MD simulations of the self-assembly process for 216 **2a** and **3a** tripeptides were performed as described previously.[4] To further investigate the molecular determinants of **2a** fibrillization, an additional set of simulations was performed with 1000 such peptides. The initial structures were generated by placing the center of mass of the peptide repeatedly on the 15 Å-spaced points of a 10x10x10 (**2a**) or 6x6x6 (**2a, 3a**) grid. Initial orientations of peptides were randomized, and the systems were solvated with water molecules.

To investigate the molecular interactions occurring in solvent between **3a** and Aβ, we performed a MD simulation of Aβ and 20 tripeptides in water solution. In this case, due to the net electrostatic charge carried by the protein, the system was neutralized by adding 13 K^+ and 10 Cl^- ions.

All the simulations were performed as follows. First, three consecutive restrained structural optimizations (up to 25,000 steps) were performed in the presence of harmonic restraints (k = 1 kcal mol^{-1} $Å^{-1}$) applied to: a) all non-hydrogenous atoms of the system; b) backbone atoms; c) C_α atoms. Reference structures at steps b) and c) were the final ones from the previous step. Next, up to 50,000 cycles of unrestrained optimization were performed. Each system was then heated to 310 K in 1 ns *via* constant-pressure-temperature (NTP) MD simulations, followed by an equilibration phase of 10 ns. Starting from the equilibrated structure, multiple MD simulations were performed for each system (see **Table S1**). Pressure and temperature were set to 1 atm and 310 K (after the equilibration phase) using the isotropic Berendsen barostat[5] and the Langevin thermostat,[6] respectively. A time step of 2 fs was used for all the simulation steps but the production runs, where it was set to 4 fs after hydrogen mass repartitioning.[7] Periodic boundary conditions were employed, and electrostatic interactions were estimated using the Particle Mesh Ewald scheme with a cutoff of 9.0 Å for the short-range evaluation in direct space and for Lennard-Jones interactions (with a continuum model correction for energy and pressure).

The AMBER force fields parm14SB[8] was employed to model the Aβ peptide and the tripeptides, while the TIP3P[9] model was used describe water molecules, and the parameters for the ions were taken from ref. 10. The TIP3P (transferable intermolecular potential with 3 points) model describes a water molecule as three interaction sites corresponding to the oxygen and the two hydrogen atoms, linked by rigid bonds. Oxygen and hydrogens bear fraction charges amounting to -0.834 and 0.417, respectively. Coulomb interactions are present between all intermolecular pairs of charges, while Van der Waals interactions are described by a single Lennard-Jones term between oxygens.

Despite its simplicity, this model is still largely employed in the simulation of biomolecules, in view of its computational efficiency and of the reasonable thermodynamic and structural description provided for liquid water without the need for three-body corrections.

Table S1. Details of the full-atom MD simulations performed for this work.

**Simulation**	N_{pep}	N_{waters}	$T_{simulation}$	c_{pep} [M]	$N_{replicas}$
**2a** self-assembly	216	~45,500	1 μs	0.25	4
**2a** self-assembly	1000	~201,000	1.5 μs	0.25	4
**3a** self-assembly	216	~45,500	1 μs	0.25	4
**3a/Aβ** interaction	20	~10,000	250 ns	n.c.	1

References

1. Case, D. A.; Ben-Shalom, I. Y.; Brozell, S. R.; Cerutti, D. S.; Cheatham, T. E.; Cruzeiro, V. W. D. III; Darden, T. A.; Duke, R. E.; Ghoreishi, D.; Giambasu, G.; Giese, T.; Gilson, M. K.; Gohlke, H.; Goetz, A. W.; Greene, D.; Harris, R.; Homeyer, N.; Huang, Y.; Izadi, S.; Kovalenko, A.; *et al.* Amber19, University of California: San Francisco, **2019**.
2. Humphrey, W.; Dalke, A.; Schulten, K., VMD: Visual Molecular Dynamics. *J. Mol. Graph.* **1996,** *14*, 33-38.
3. Wälti, M. A.; Ravotti, F.; Arai, H.; Glabe, C. G.; Wall, J. S.; Böckmann, A.; Güntert, P.; Meier, B. H.; Riek, R., Atomic-Resolution Structure of a Disease-Relevant Aβ(1–42) Amyloid Fibril. *Proc. Natl. Acad. Sci. U. S. A.* **2016,** *113*, E4976.
4. Garcia, A. M.; Iglesias, D.; Parisi, E.; Styan, K. E.; Waddington, L. J.; Deganutti, C.; De Zorzi, R.; Grassi, M.; Melchionna, M.; Vargiu, A. V.; Marchesan, S. Chirality Effects on Peptide Self-Assembly Unraveled from Molecules to Materials. *Chem* **2018,** *4*, 1862-1876.
5. Berendsen, H. J. C.; Postma, J. P. M.; Van Gunsteren, W. F.; Dinola, A.; Haak, J. R. Molecular-Dynamics with Coupling to an External Bath. *J. Chem. Phys.* **1984**; 81: 3684-3690.
6. Feller, S. E.; Zhang, Y.; Pastor, R. W.; Brooks, B. R. Constant Pressure Molecular Dynamics Simulation: The Langevin Piston Method. *Time* **1995**, *103*, 4613-4621.
7. Hopkins, C. W.; Le Grand, S.; Walker, R. C.; Roitberg, A. E. Long-Time-Step Molecular Dynamics through Hydrogen Mass Repartitioning. *J. Chem. Theory Comput.* **2015**, 11, 4, 1864–1874.
8. Maier, J. A.; Martinez, C.; Kasavajhala, K.; Wickstrom, L.; Hauser, K. E.; Simmerling, C. ff14SB: Improving the Accuracy of Protein Side Chain and Backbone Parameters from ff99SB. *J. Chem. Theory Comput.* **2015**, *11*, 3696–3713.

9. Jorgensen, W. L.; Chandrasekhar, J.; Madura, J. D. Comparison of Simple Potential Functions for Simulating Liquid Water. *J. Chem. Phys.* **1983**, *79*, 926.
10. Joung, I. S.; Cheatham, T. E. III. Determination of Alkali and Halide Monovalent Ion Parameters for Use in Explicitly Solvated Biomolecular Simulations. *J. Phys. Chem. B* **2008**, *112*, 9020–9041.

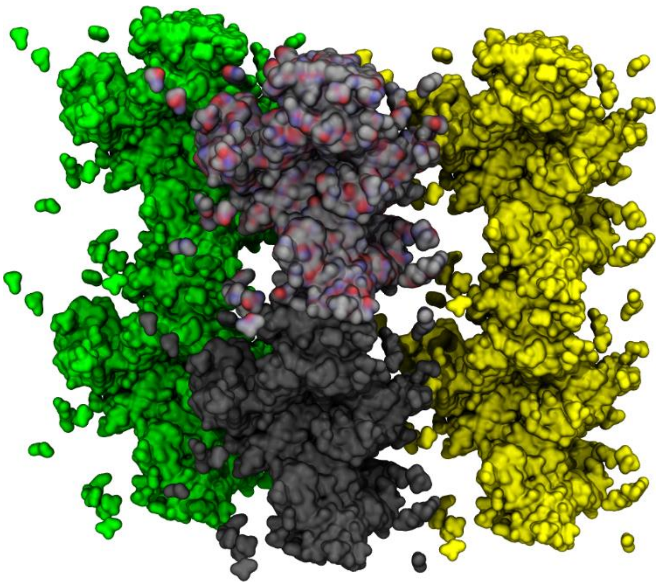

Fig. S46. Assembly of 1,000 DLL **2a** peptides as resulting from 1.5 microsecond long MD simulations in water solution. Six replicas were run. The surface colored by atom type (C, N, O in grey, blue and red, respectively) is the one in the primary simulation box. That in grey extends along the x direction, while the two other rod-like assemblies represent adjacent images.

S7. DLS data

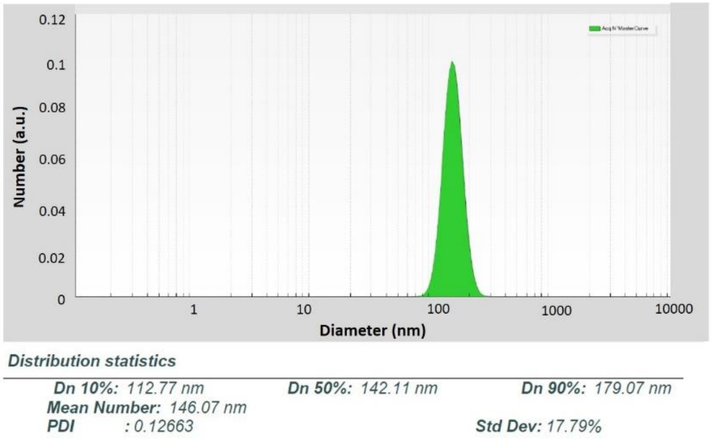

Fig. S47. Representative DLS data of **3a** samples.

S8. FT-IR spectra.

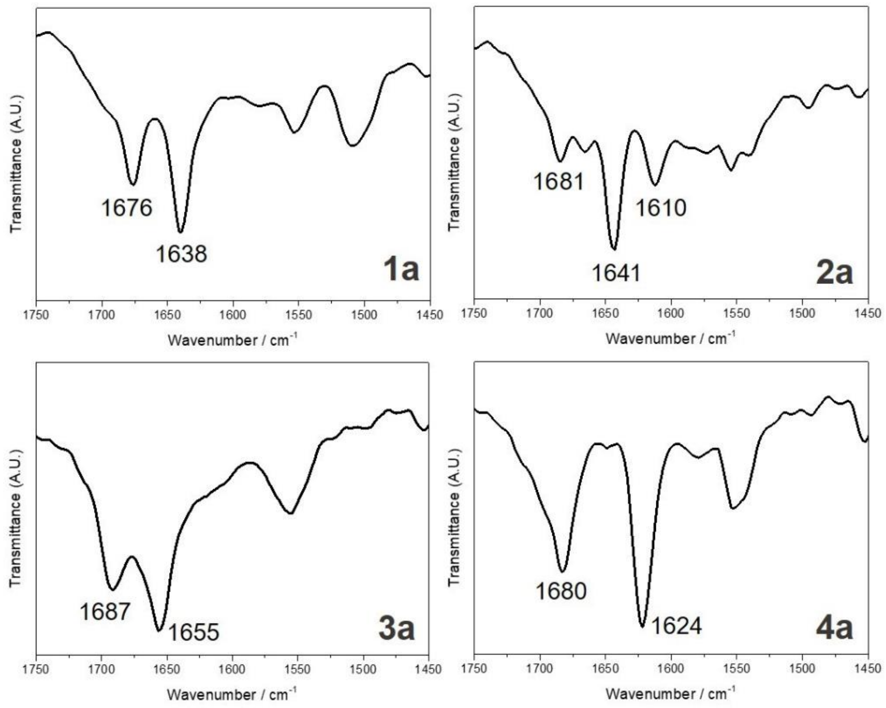

Fig S48. ATR-FT-IR spectra of tripeptide enantiomers **1a**-**4a**. Spectra were recorded using 24-hours aged samples. Numbers indicated the most relevant signals in the amide I region.

S9. XRD data

Compound 1a (CCDC 2021319)

Needle-shaped single crystals of the peptide were collected with a loop, cryoprotected by dipping the crystals polyethylene glycol with average molecular weight 200 g/mol, PEG 200, and stored frozen in liquid nitrogen. Crystal were mounted on the diffractometer at the synchrotron Elettra, Trieste (Italy), beamline XRD1, using the robot present at the facility. Temperature was kept at 100 K by a stream of nitrogen on the crystal. Diffraction data were collected by the rotating crystal method using synchrotron radiation, wavelength 0.70 Å, rotation interval 2°/image, crystal-to-detector distance of 85 mm. A total of 90 images were collected. Reflections were indexed and integrated using the software Mosflm,[1] space group $P2_1$ was determined using POINTLESS.[2] The resulting data set was scaled using AIMLESS.[3] Phase information were obtained by direct methods using the software SHELX-T.[4] Refinements cycles were conducted with SHELXL-18,[5] operating through the WinGX GUI,[6] by full-matrix least-squares methods on F^2.

Congruence of structure model and calculated electron density was inspected using the software Coot.[7] The asymmetric unit contains a single molecule of the peptide. Hydrogen atoms were added at geometrically calculated positions and refined isotropically. All non-hydrogen atoms within the asymmetric unit have been refined with anisotropic thermal parameters. Unit cell parameters, scaling statistics, and refinement statistics are reported in **Table S2**.

Compound 3a (CCDC2026055)

A stick-shaped single crystal of the peptide was collected with a loop, cryoprotected by dipping the crystal in polyethylene glycol with average molecular weight 200 g/mol, PEG 200, and stored frozen in liquid nitrogen. The crystal was mounted on the diffractometer at the synchrotron Elettra, Trieste (Italy), beamline XRD1, using the robot present at the facility. Temperature was kept at 100 K by a stream of nitrogen on the crystal. Diffraction data were collected by the rotating crystal method using synchrotron radiation, wavelength 0.70 Å, rotation interval 1°/image, crystal-to-detector distance of 85 mm. A total of 180 images were collected. Reflections were indexed and integrated using the software Mosflm,[1] space group P21 was determined using POINTLESS.[2] The resulting data set was scaled using AIMLESS.[3] Phase information were obtained by direct methods using the software SIR 2014.[8] Refinements cycles were conducted with SHELXL-18,[5] operating through the WinGX GUI,[6] by full-matrix least-squares methods on F2. Congruence of structure model and calculated electron density was inspected using the software Coot.[7] The asymmetric unit contains 10 crystallographically independent molecules of the peptide. In the residual electron density of the asymmetric unit, 12 water molecules were located, 7 of which in positions at full occupancy, 5 in positions statistically occupied in 50% of the unit cells. Restraints were applied to bond lengths and angles of the proline moiety of two crystallographically independent peptides, using the cards DFIX and DANG of the SHELXL-18 software,[5] in particular when a statistical occupancy of two close positions was observed. In addition, restrains were applied to keeps similar values for anisotropic displacement parameters for adjacent atoms in the direction of bonds. Hydrogen atoms of the peptide molecules were added at geometrically calculated positions and refined isotropically. When a clear electron density could be observed, hydrogen atoms of the water molecules at full occupancy were added considering the hydrogen bonding pattern, and refined with restrains on bond lengths and angles, using the cards DFIX and DANG of the SHELXL-18 software.[5] All the atoms,

except the hydrogen atoms, within the asymmetric unit have been refined with anisotropic thermal parameters. Unit cell parameters, scaling statistics, and refinement statistics are reported in **Table S2**.

Compound 4b (CCDC2021318)

A stick-shaped single crystal of the peptide was collected with a loop, cryoprotected by dipping the crystal in glycerol and stored frozen in liquid nitrogen. The crystal was mounted on the diffractometer at the synchrotron Elettra, Trieste (Italy), beamline XRD1, using the robot present at the facility. Temperature was kept at 100 K by a stream of nitrogen on the crystal. Diffraction data were collected by the rotating crystal method using synchrotron radiation, wavelength 0.70 Å, rotation interval 1°/image, crystal-to-detector distance of 85 mm. A total of 180 images were collected. Reflections were indexed and integrated using the XDS package,[9] space group C2 was determined using POINTLESS,[2] and the resulting data set was scaled using AIMLESS.[3] Phase information were obtained by direct methods using the software SHELXS.[10] Refinement cycles were conducted with SHELXL-14,[5] operating through the WinGX GUI,[6] by full-matrix least-squares methods on F2. Congruence of structure model and calculated electron density was inspected using the software Coot.[7] The asymmetric unit contains a single molecule of the peptide and a molecule of water in a special position, with the oxygen atom located along the 2-fold symmetry axis, resulting in an occupancy of 50% in the asymmetric unit. During refinement, no restraints were applied on distances, angles or thermal parameters of the peptide or the water molecule. Hydrogen atoms of the peptide were added at geometrically calculated positions and refined isotropically, with thermal parameters dependent on those of the attached atom. All non-hydrogen atoms were refined with anisotropic thermal parameters. Unit cell parameters, scaling statistics, and refinement statistics are reported in **Table S2**.

Table S2: Crystallographic data.

	**Compound 1a** CCDC2021319	**Compound 3a** CCDC2026055	**Compound 4b** CCDC2021318
Formula	$C_{23}H_{27}N_3O_4$	$10C_{23}H_{27}N_3O_4 \cdot 9.5H_2O$	$C_{23}H_{27}N_3O_4 \cdot 0.5H_2O$
Temperature (K)	100	100	100
Wavelength (Å)	0.7	0.7	0.7
Crystal system	Monoclinic	Monoclinic	Monoclinic
Space group	$P\ 2_1$	$P\ 2_1$	$C\ 2$
a (Å)	5.321(1)	13.280(2)	27.040(5)
b (Å)	11.574(2)	22.700(4)	5.550(1)
c (Å)	17.058(3)	39.530(5)	19.320(4)
α (°)	90	90	90
β (°)	97.22(3)	98.220(8)	134.45(3)
γ (°)	90	90	90
V ($Å^3$)	1042.2(4)	11794(3)	2070(1)
Z, ρcalc (g/cm^3)	2, 1.305	2, 1.201	4, 1.343
μ (mm^{-1})	0.086	0.053	0.090
F (000)	436	4550	892
Data collection θ range	2.1 - 28.326	1.021 - 28.394	1.454 - 28.633
Refl. Collected / unique	6991 / 2504	65886 / 31222	14963 / 3014
Rint	0.062	0.075	0.065
Completeness (%)	89.4	98.5	98.9
Data/Restraints/Parameters	2504 / 1 / 272	31222 / 216 / 2890	3014 / 1 / 297
GooF	1.052	1.001	1.078
R1, wR2 [I>2σ(I)]	0.0454 / 0.1176	0.0818 / 0.2111	0.0412 / 0.1046
R1, wR2 all data	0.0483 / 0.1196	0.0889 / 0.2197	0.0451 / 0.1075

References:

1. Battye, T. G.; Kontogiannis, L.; Johnson, O.; Powell, H. R.; Leslie, A. G. iMOSFLM: A New Graphical Interface for Diffraction-Image Processing with MOSFLM. *Acta Cryst.* **2011,** *D67*, 271-281.

2. Evans, P. R. Scaling and Assessment of Data Quality. *Acta Cryst.* **2006**, *D62*, 72−82.

3. Evans, P. R., Murshudov, G. N. How Good Are My Data and What Is the Resolution? *Acta Cryst.* **2013**, *D69*, 1204−1214.

4. Sheldrick G. M. SHELXT - Integrated Space-Group and Crystal-Structure Determination *Acta Cryst.* **2015**, *A71*, 3-8.

5. Sheldrick, G. M. SHELXT – Integrated Space-Group and Crystal-Structure Determination *Acta Cryst.* **2015**, *C71*, 3–8.

6. Farrugia, L.J. WinGX and ORTEP for Windows: An Update. *J. Appl. Cryst.* **2012**, *45*, 849−854.

7. Emsley, P.; Cowtan, K. Coot: Model-Building Tools for Molecular Graphics. *Acta Cryst*. **2004**, *D60*, 2126-2132.

8. Burla, M. C.; Caliandro, R.; Carrozzini, B.; Cascarano, G. L.; Cuocci, C.; Giacovazzo, C.; Mallamo, M.; Mazzone, A.; Polidori, G. Crystal Structure Determination and Refinement *via* SIR2014. *J. Appl. Cryst.* **2015**, *48*, 306-309.

9. Kabsch, W. XDS. *Acta Cryst.* **2010**, *D66*, 125−132.

10. Sheldrick, G. M. A Short History of SHELX. *Acta Cryst*. **2008**, *A64*, 112-122.

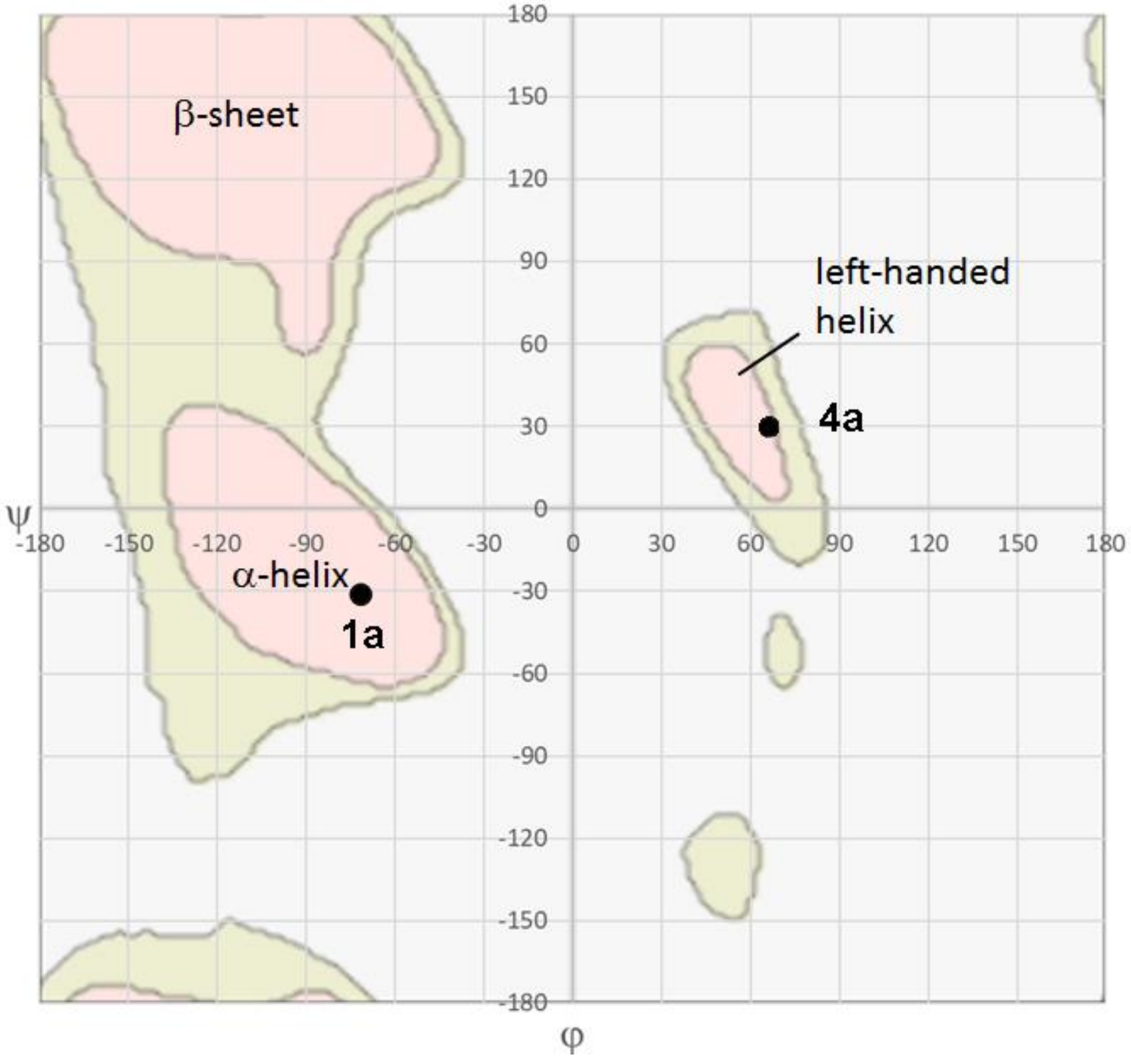

Fig. S49. Ramachandran plot for the crystal structure of **1a** and **4a** (black circles).

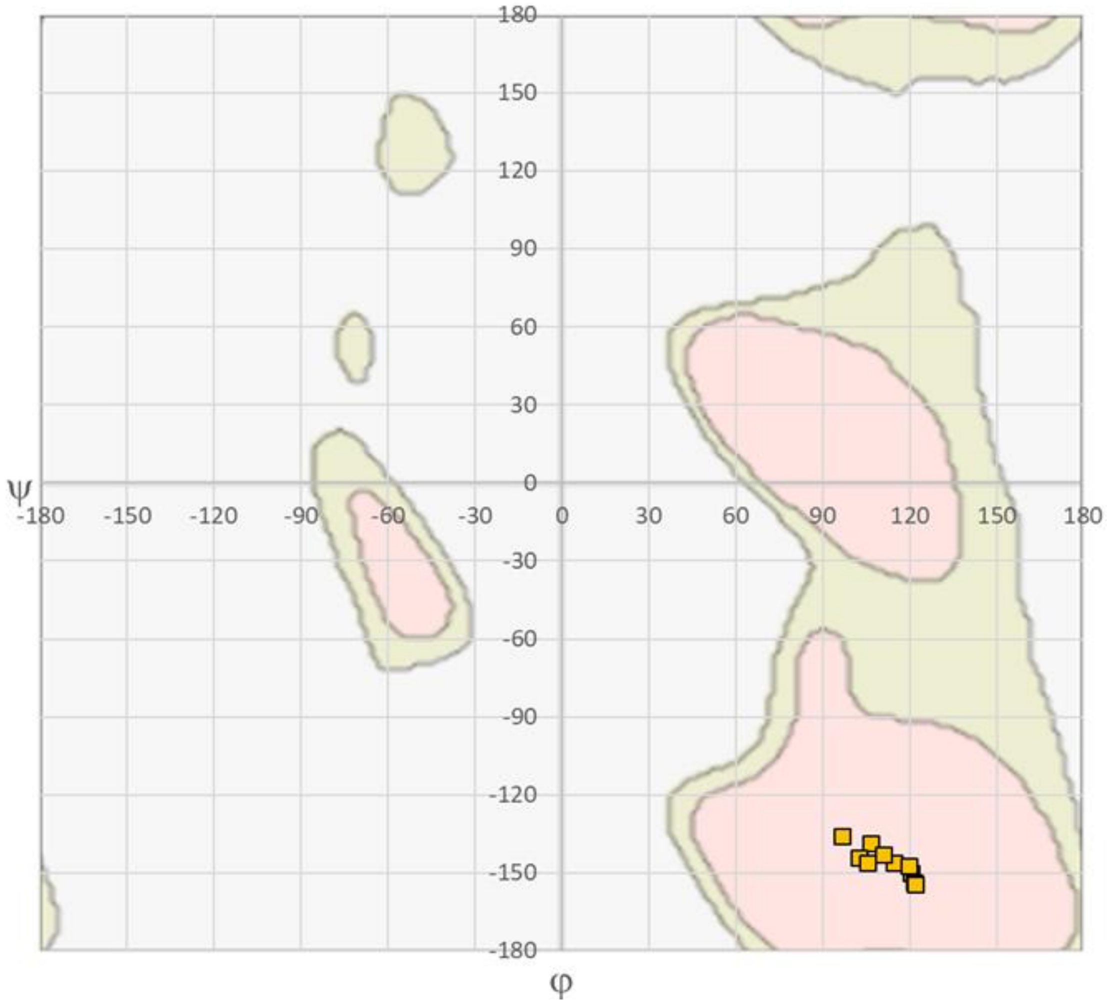

Fig. S50. Inverted Ramachandran plot for the crystal structure of LDL **3a** (orange squares refer to the 10 independent molecules).

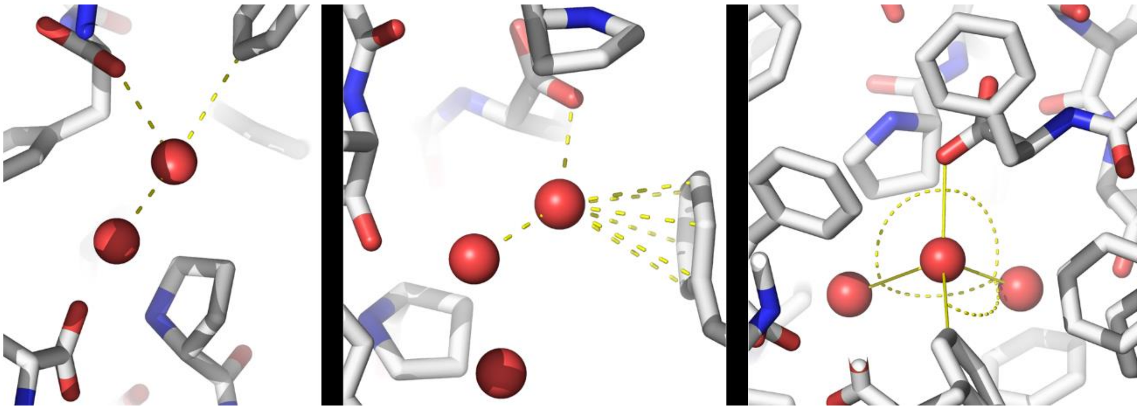

Fig. S51. Crystal structure of **3a**. Three snapshots show the position of water molecules that were superimposable in the structures crystallized from methanol/water or from phosphate buffer.

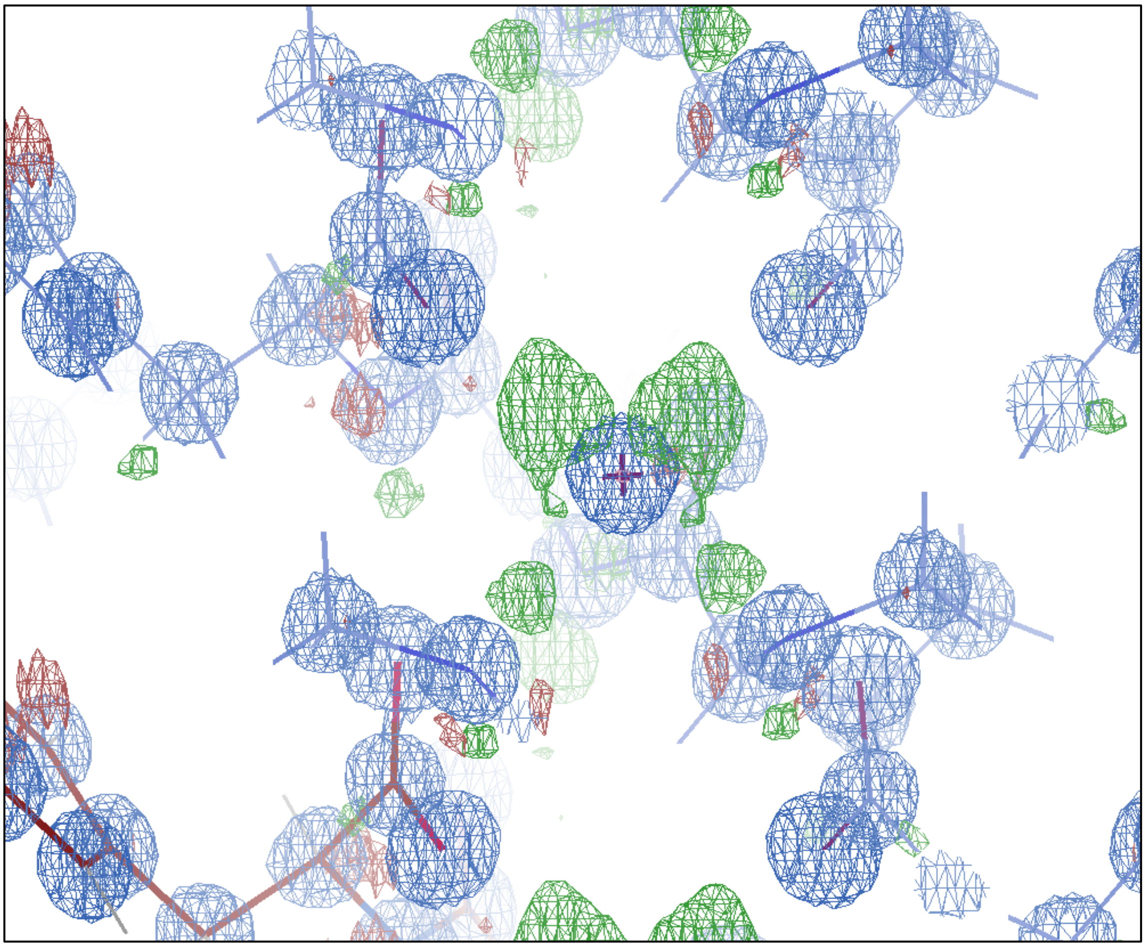

Fig.S52. Crystal structure of **4b** (CCDC2021318). Strong electron density for the water molecule proved the very stable H-bonds that keep it between the peptide chains. Electron density was clearly visible also for the hydrogen atoms of the water and of the N-terminus.

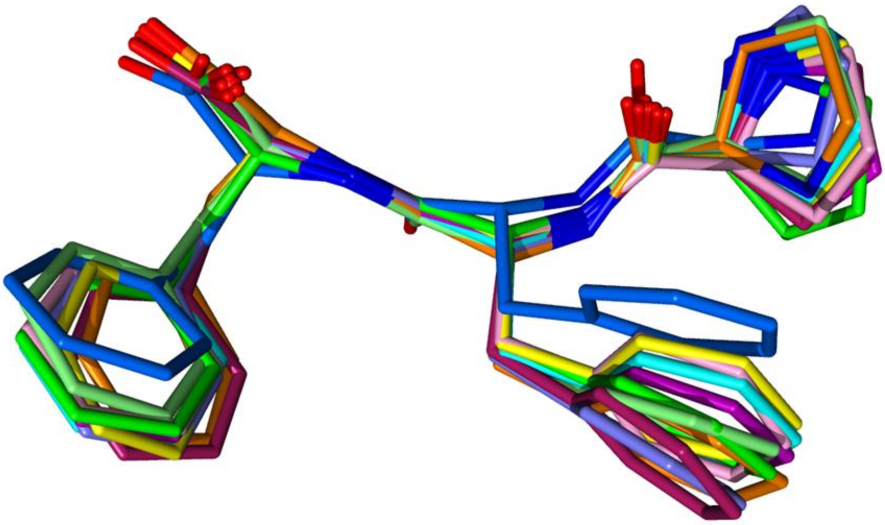

Fig. S53. Superimposition of the 10 independent molecules of the crystal structure of **3a** revealed the same conformation.

S10. Aβ(1-42) inhibition data.

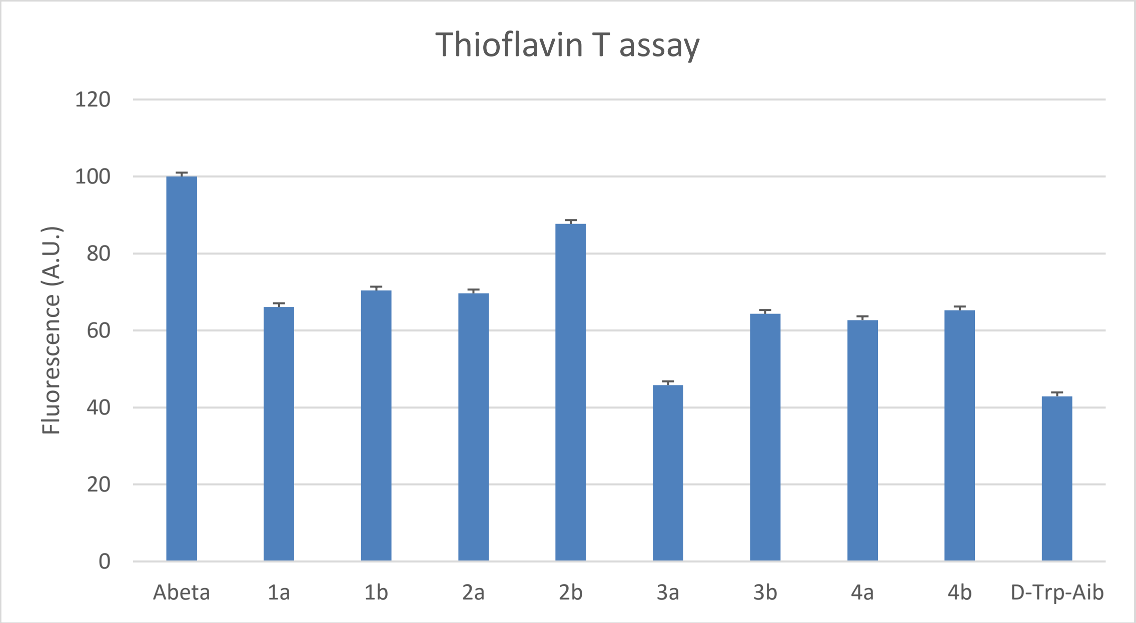

Fig. S54. Thioflavin T assay at 48 hours revealed **3a** was the most promising inhibitor, with analogous activity as the positive control D-Trp-Aib.

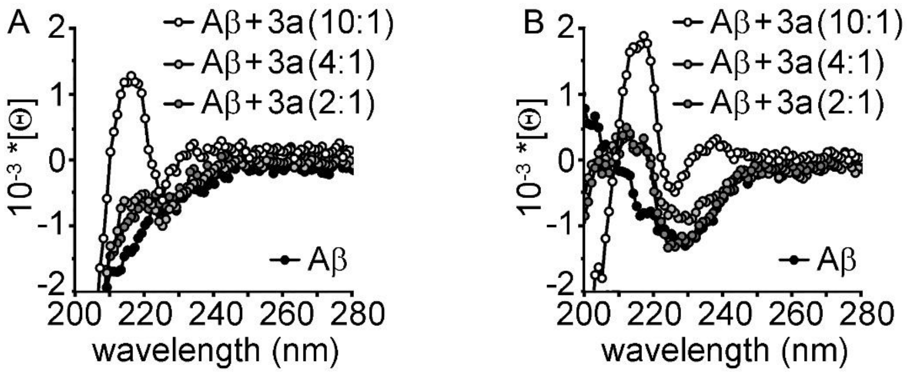

Fig S55. CD spectra of 15 μM Aβ(1-42) in the presence of **3a** at the indicated concentrations (A) fresh and (B) after incubation for 24 hours at 37 ºC.

S11. Thioflavin T fluorescence on tripeptides

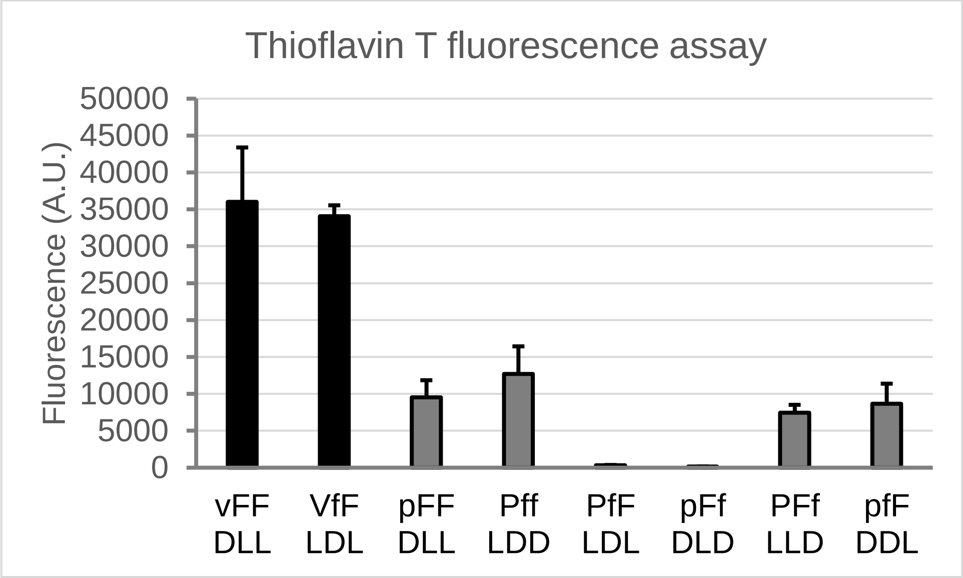

Fig. S56. Thioflavin T fluorescence assay on peptides at 20 mM, except for **2a** and **2b** that were probed at the mgc of 24 mM (at 20 mM the fluorescence was negligible). For reference, the Val-Phe-Phe sequence DLL and LDL peptides (black columns) were previously reported as the only gelators of the four stereoisomers (LLL, DLL, LDL, LLD) in S. Marchesan *et al. J. Mater. Chem. B* **2015**, *3*, 8123. Homochiral Pro-Phe-Phe (LLL or DDD) is not reported because of the presence of heterogeneous macroscopic aggregates at 20 mM that did not allow reproducible measurements.
